# Mouse *Dspp* frameshift model of human *dentinogenesis imperfecta*

**DOI:** 10.1038/s41598-021-00219-4

**Published:** 2021-10-19

**Authors:** Tian Liang, Yuanyuan Hu, Hong Zhang, Qian Xu, Charles E. Smith, Chuhua Zhang, Jung-Wook Kim, Shih-Kai Wang, Thomas L. Saunders, Yongbo Lu, Jan C.-C. Hu, James P. Simmer

**Affiliations:** 1 Department of Biologic and Materials Sciences, University of Michigan School of Dentistry, 1011 North University, Ann Arbor, MI 48108 USA; 2 Department of Anatomy and Cell Biology and Faculty of Dentistry, Facility for Electron Microscopy Research, McGill University, Montreal, QC H3A 2B2 Canada; 3 Department of Biomedical Sciences and Center for Craniofacial Research and Diagnosis, Texas A&M University College of Dentistry, 3302 Gaston Ave., Dallas, TX 75246 USA; 4 Department of Molecular Genetics, School of Dentistry & Dental Research Institute, Seoul National University, Seoul, Korea; 5 Department of Pediatric Dentistry, School of Dentistry & Dental Research Institute, Seoul National University, Seoul, Korea; 6 Department of Dentistry, National Taiwan University School of Dentistry, No.1, Changde St., Jhongjheng District, Taipei City 100, Taiwan; 7 Department of Internal Medicine, University of Michigan Medical School, 1150 West Medical Center Drive, MSRBII Room 2570, Ann Arbor, MI 41809 USA

**Keywords:** Developmental biology, Genetics, Structural biology, Diseases

## Abstract

Non-syndromic inherited defects of tooth dentin are caused by two classes of dominant negative/gain-of-function mutations in dentin sialophosphoprotein (*DSPP*): 5′ mutations affecting an N-terminal targeting sequence and 3′ mutations that shift translation into the − 1 reading frame. *DSPP* defects cause an overlapping spectrum of phenotypes classified as *dentin dysplasia* type II and *dentinogenesis imperfecta* types II and III. Using CRISPR/Cas9, we generated a *Dspp*^−1fs^ mouse model by introducing a FLAG-tag followed by a single nucleotide deletion that translated 493 extraneous amino acids before termination. Developing incisors and/or molars from this mouse and a *Dspp*^P19L^ mouse were characterized by morphological assessment, bSEM, nanohardness testing, histological analysis, in situ hybridization and immunohistochemistry. *Dspp*^P19L^ dentin contained dentinal tubules but grew slowly and was softer and less mineralized than the wild-type. *Dspp*^P19L^ incisor enamel was softer than normal, while molar enamel showed reduced rod/interrod definition. *Dspp*^−1fs^ dentin formation was analogous to reparative dentin: it lacked dentinal tubules, contained cellular debris, and was significantly softer and thinner than *Dspp*^+/+^ and *Dspp*^P19L^ dentin. The *Dspp*^−1fs^ incisor enamel appeared normal and was comparable to the wild-type in hardness. We conclude that 5′ and 3′ *Dspp* mutations cause dental malformations through different pathological mechanisms and can be regarded as distinct disorders.

## Introduction

The principal organic components of tooth dentin are type I collagen and dentin sialophosphoprotein (DSPP)^1^. Genetic studies have demonstrated the importance of the genes encoding type I collagen (*COL1A1*; *COL1A2*)^2^ and *DSPP*^3^ in the etiologies of autosomal dominant inherited dentin defects. Defects in type I collagen cause syndromes, such as osteogenesis imperfecta (OI) and Ehlers-Danlos syndrome (EDS)^4,5^. Nonsyndromic inherited dentin defects are distinguished clinically as dentin dysplasia type II (DD-II), dentinogenesis imperfecta type II (DGI-II) and dentinogenesis imperfecta type III (DGI-III)^6^ and are caused by mutations in *DSPP*. Sharing a common causative gene suggested that the three clinical conditions simply reflect variations in severity of a single disease^7^ and are caused by the same pathology^8^. In fact, it’s more complicated.

Isolated dentin defects described as “dentinogenesis imperfect” are transmitted as an autosomal dominant trait that occurs once in 6000 to 8000 children in the general population^9^. There are no reports of recessive dentin defects caused by mutations in both alleles of *DSPP*. However, homozygous *Dspp* knockout mice show severe dentin defects^10^, so the recessive condition in humans must exist, but be rare and not yet described. About 50 different pathogenic mutations in the *DSPP* gene have been reported^11^. Analyses of the types of *DSPP* mutations that cause inherited dentin defects demonstrate that they essentially fall into two distinct groups: 5′ mutations that alter the first three amino acids following the signal peptide and 3′ indels in exon 5 (the last coding exon) that cause a shift into the − 1 reading frame^12,13^. The pattern of mutations strongly supports the conclusion that simple loss-of-function mutations do not cause dominant dentin malformations in humans^14^. In mice, *Dspp* heterozygotes show either no dentin phenotype^10^, or mild dentin defects not evident until old age^15^.

Recently a known pathogenic *DSPP* 5′ mutation (p.Pro17Leu)^16–18^ was introduced into mice (p.Pro19Leu, accordingly) and characterized^19^. An in vitro splicing assay had previously shown that this mutation did not alter pre-mRNA splicing but caused the mutant protein to be retained in the endoplasmic reticulum (ER)^17^. Similar dental malformations were observed in both heterozygous (*Dspp*^+/P19L^) and homozygous (*Dspp*^P19L/P19L^) mice. *Dspp*^P19L^ (a designation that includes both the heterozygous and homozygous mice) dentin grew more slowly than the wild-type, so the dentin layer was thinner, and the dental pulp enlarged. The dentin contained dentinal tubules with enlarged peritubular dentin. Secretion of the mutant *Dspp*^P19L^ protein was impaired and it accumulated within the ER. The enamel underwent accelerated attrition.

Here we report the generation and characterization of a genetically modified *Dspp*^−1fs^ mouse with a FLAG epitope within the DSP-DPP cleavage sequence (for sensitive and specific immunodetection) followed by a single nucleotide deletion that introduced a − 1 frameshift that makes it analogous to the group of 3′ mutations in human exon 5. We compare its dental phenotype to that of *Dspp*^P19L^ mice.

## Results

### Confirmation of the ***Dspp***^−1fs^ mouse model

The CRISPR/Cas9 gene-editing and a synthetic donor DNA introduced a DYKDDDK flag-tag coding sequence immediately preceding the dentin phosphoprotein (DPP) N-terminal amino acid sequence (Asp-Asp-Pro-Lys; DDPK), and a single nucleotide deletion (c.1365delG) immediately downstream to the DDPK sequence (Fig. S1). The predicted effect of the single base deletion was translation of 493 extraneous amino acids following the frameshift (Figs. S1B, S2). From the screening of 48 alleles, the correctly targeted allele was identified in two independent founders (Fig. S3A), and simple methods of PCR genotyping were developed (Fig. S3B,C). Both founders were preserved as separate lines to produce offspring. G2 offspring were evaluated using Sanger sequencing and confirmed to have no untargeted sequence variations in the *Dspp* coding exons and intron–exon borders. To diminish the possibility of off-target effects and phenotypic variations caused by a mixed genomic background, we backcrossed the G2 with C57BL/6N for an additional 5 generations (G7).

During the initial screening, a mouse expressing an untargeted spontaneous *Dspp* mutation (c.1380_1381insT) was detected that terminated translation after the first 9 amino acids (DDPKSSDES*) of the DPP domain, which we designated *Dspp*^−DPP^ (Fig. S2C). The inserted T introduced an *MseI* (5′-TTAA-3) restriction site that was exploited to develop a convenient genotyping strategy (Fig. S3C). This mouse is being characterized but is not discussed further here.

### Phenotype assessment by dissection microscopy

**Figure 1:**
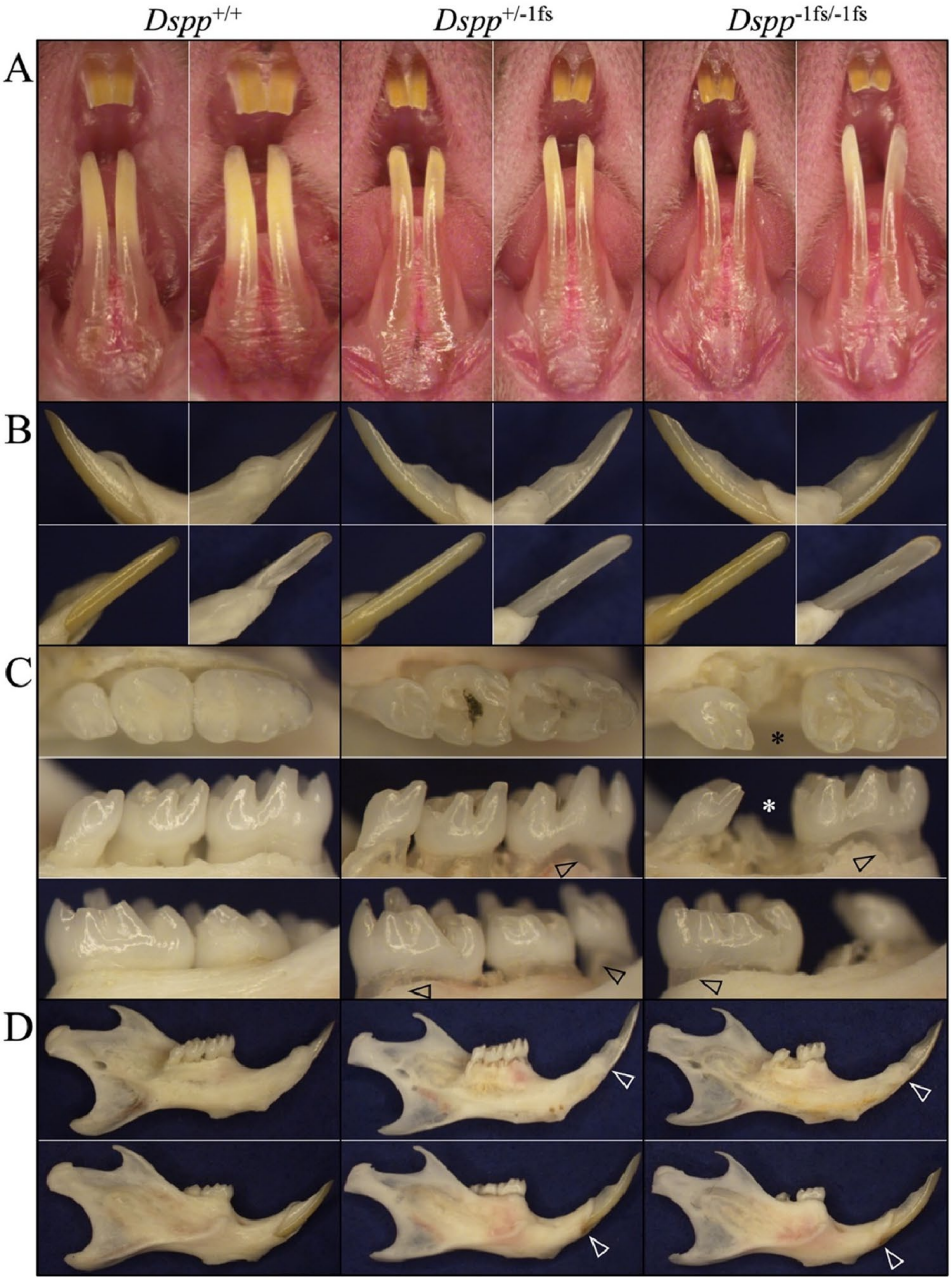
Dissection microscopy photographs comparing 7-week *Dspp*^+*/*+^, *Dspp*^+*/*−1fs^, and *Dspp*^−1fs*/*−1fs^ Mice. (**A**) Frontal photos demonstrate that *Dspp*^+*/*−1fs^ and *Dspp*^−1fs*/*−1fs^ mouse incisors are thinner (mesial-distally) than the wild-type (*Dspp*^+*/*+^). The *Dspp*^+*/*−1fs^ and *Dspp*^−1fs*/*−1fs^ mouse incisors also show an extend apical region of increased translucency much further incisally, apparently due to a reduction or delay in dentin mineralization. (**B**) Close up photographs of the erupted mandibular incisor tip. Clockwise from the upper right: mesial, distal, labial, lingual. Hyper-eruption of the *Dspp*^+*/*−1fs^, and *Dspp*^−1fs*/*−1fs^ incisors or diminished alveolar bone causes their longer apparent length outside the bone. (**C**) Occlusal (top), lingual, and buccal (bottom) views of the mandibular molars. Overall enamel crown morphology is normal in the *Dspp*^+*/*−1fs^ and *Dspp*^−1fs*/*−1fs^ mouse incisors. The root dentin is translucent (arrowheads) and the height of the alveolar crest is lower relative to the cemento-enamel junction in the *Dspp*^+*/*−1fs^, and *Dspp*^−1fs*/*−1fs^ molars. (**D**) Lingual (top) and lateral (bottom) views of the mandibles. Increased length of the erupted portion of the incisor beyond the buccal alveolar crest (arrowheads). The mandible is transparent in some areas (showing through the color of the dark blue background), suggesting decreased mineralization.

At 7-weeks, the previously described *Dspp*^P19L^ mouse mandibular incisors showed reduced iron staining and increased attrition, while the molars showed increased enamel attrition and surface roughness (Fig. S4). The *Dspp*^+/−1fs^ and *Dspp*^−1fs/−1fs^ (together designated as *Dspp*^−1fs^) mice presented with the dentin being visibly more translucent in the incisors (Fig. 1A,B) and in the roots of the molars (Fig. 1C). Catastrophic fracturing of a first or second molar crown was often observed in *Dspp*^−1fs^ mice (Fig. 1C). In contrast, no whole crown failures or change in dentin translucency was observed in *Dspp*^P19L^ mice^19^. The height of the alveolar crest of bone in both the molars (Fig. 1C) and incisors (Fig. 1D) appeared to be lower than in the wild-type. The mandibular incisors looked longer than normal, suggesting possible hypereruption or alveolar bone recession that was not investigated further.

### Assessment of mandibular molars and incisors by bSEM

The characterization of continuously growing mouse mandibular incisors at 1 mm increments by bSEM is a standard means of assessing the progress of dentin and enamel mineralization throughout tooth development^20,21^. We analyzed 7-week mandibular incisor formation by bSEM at 1 mm increments in *Dspp*^+/+^ (Fig. S5), *Dspp*^+/P19L^ (Fig. S6A,B), *Dspp*^P19L/P19L^ (Fig. S7A,B), *Dspp*^+/−1fs^ (Fig. S8A,B), and *Dspp*^−1fs/−1fs^ (Fig. S9A,B) mice. These studies allowed us to observe the thickness of the dentin and enamel layers, and to assess the formation of dentinal tubules and enamel rod structures in the mandibular incisors.

Mandibular first molars typically erupt on day 14 (D14). We used bSEM to image mandibular molar surfaces in *Dspp*^+/+^, *Dspp*^+/−1fs^, *Dspp*^−1fs/−1fs^, *Dspp*^+/P19L^, and *Dspp*^P19L/P19L^ mice at 14 days (D14), following completion of the first molar crowns, but immediately prior to their eruption into the oral cavity (Fig. S10). We also imaged the D14 first molars using bSEM after they had been cross-sectioned to assess the formation of dentinal tubules and enamel rod/interrod structures in *Dspp*^+/+^ (Fig. S11), *Dspp*^+/P19L^ (Fig. S12), *Dspp*^P19L/P19L^ (Fig. S13), *Dspp*^+/−1fs^ (Fig. S14A,B), and *Dspp*^−1fs/−1fs^ (Fig. S15A–D) mice. Finally, we imaged erupted mandibular first molars at 7-weeks, to assess attritions levels following 5-weeks of function in the oral cavity (Fig. S16).

**Figure 2:**
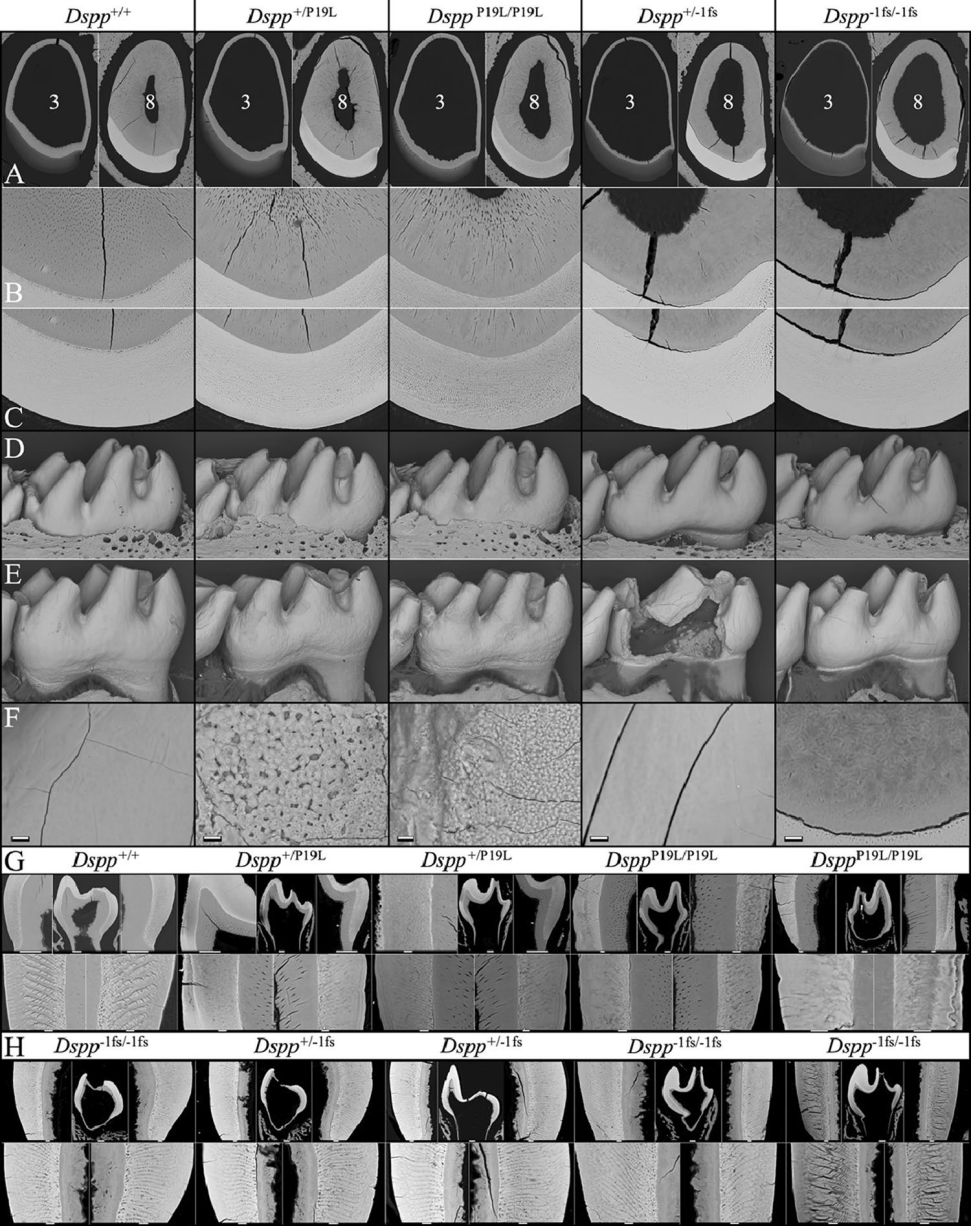
bSEM imaging of tooth defects in *Dspp*^+*/*P19L^, *Dspp*^P19L*/*P19L^, *Dspp*^+*/*−1fs^, and *Dspp*^−1fs*/*−1fs^ Mice. (**A**) 7-week-old incisor cross-section (75×) from level 3 (left) and level 8 (right). Enamel is of normal thickness and contour in all genotypes. The dentin layer (pulp to DEJ) is somewhat thinner in *Dspp*^P19L^, and thinner in *Dspp*^−1fs^ incisors relative to the WT. (**B**) Level 8 images (400×) of dentin show complete absence of dentinal tubules in *Dspp*^−1fs^ mouse incisors. (**C**) Level 8 images (400×) of enamel show normal thickness and rod patterns, although the rod patterns stand out more in the *Dspp*^P19L^ mice, suggesting hypomineralization. (**D**) Erupting D14 mandibular first molars (70×) show normal shape and contour, although the *Dspp*^P19L^ distal-buccal cusps appear thinner and exhibit enamel surface roughness. (**E**) At 7 weeks, the erupted *Dspp*^P19L^ molars show enamel surface roughness and accelerated attrition, whereas the *Dspp*^−1fs^ molars show normal surface texture, accelerated attrition, and sometimes crown failures. (**F**) 7-week (1000×; bar = 10 µm) molar surface images showing poorly mineralized enamel surface in *Dspp*^P19L^ molars, normal enamel surface in the *Dspp*^+/−1fs^ molar. Also shown is a higher magnification (1000×) view of irregular growth lines roughly parallel to the DEJ in *Dspp*^−1fs*/*−1fs^ incisor dentin at level 8, which is also typical of *Dspp*^+/−1fs^ dentin (not shown). (**G,H**) bSEM images of D14 molar cross-sections. Scale bars: molar cross-sections, 100 µm; all others, 10 µm. The *Dspp*^P19L^ molar enamel is unevenly mineralized and lacks the clearly defined rod/interrod structures of wild-type enamel, while the dentin contains well-defined tubular structures. (**H**) bSEM images of D14 molar cross-sections. The *Dspp*^−1fs^ mouse molar enamel show clearly defined rod/interrod structures, while the dentin is extremely thin and lacks dentinal tubules.

The seminal findings of these bSEM analyses are shown in Fig. 2. Continuously growing 7-week mandibular incisor sections at levels 3 and 8 (3 or 8 mm from the apical loop, respectively) show the incisor at two developmental locations: (1) where the enamel layer has completed secretory stage and is entering the maturation stage (level 3), and (2) where the enamel layer has completed maturation stage (level 8, approximately at the buccal alveolar crest) but has not yet erupted into the oral cavity (Fig. 2A). All the *Dspp* modified mouse incisors exhibited a dentin layer that was thinner than the wild-type and consequently, the dental pulp was enlarged (Fig. 2A). However, dentin was thinnest in the *Dspp*^−1fs^ incisors, which also lacked dentinal tubules (Fig. 2A,B, and F right). Although the dentin was thinner than normal in the *Dspp*^P19L^ incisors, it was not as thin as the dentin in the *Dspp*^−1fs^ mice, and dentinal tubules were evident in *Dspp*^P19L^ but not in *Dspp*^−1fs^ incisors (Fig. 2A,B). The internal surface of the dentin (junction between dentin matrix and odontoblast) was rough and bumpy in *Dspp*^P19L^ mice and especially so in *Dspp*^−1fs^ mice. The incisor enamel from all genotypes achieved its normal shape and contour (Fig. 2A,C), but the outlines of enamel rods were more conspicuous in the *Dspp*^P19L^ enamel (Fig. 2C), suggesting enamel hypomineralization.

The D14 *Dspp*^P19L^ erupting first molars showed relatively normal crown morphology but exhibited a roughened enamel surface texture and the distal buccal cusp was perceptibly thinner (Fig. 2D). The enamel surfaces of D14 *Dspp*^−1fs^ erupting first molars were indistinguishable from the wild-type, showing normal shape, contour, and surface properties (Fig. 2D). At 7 weeks, following 5 weeks of function in the oral cavity, *Dspp*^−1fs^ and *Dspp*^P19L^ mandibular first molars all showed evidence of fractured cusp tips and accelerated attrition, but first or second molar whole crown failures were only observed in *Dspp*^−1fs^ mice (Figs. 1C, 2E). Crown failures were not observed in *Dspp*^P19L^ mandibular molars at 7 weeks, but rough patches of poorly mineralized enamel on the crown surfaces were evident (Fig. 2C,E). Alveolar bone recession was apparent at 7 weeks in *Dspp*^−1fs^ molars and incisors (Fig. 1, Suppl Fig. S16), and sometimes in *Dspp*^P19L^ molars (Fig. 2E). Alveolar bone recession has been reported in patients with p.(Prol17Leu) defects^18^.

The internal structures of the cross-sectioned D14 first molar dentin and enamel layers were distinctively different in *Dspp*^P19L^ and *Dspp*^−1fs^ mice (Fig. 2G,H). As in incisors, dentinal tubules were observed oly in wild-type and *Dspp*^P19L^ molars (Fig. 2G), but not in *Dspp*^−1fs^ molars (Fig. 2H). The *Dspp*^P19L^ molar enamel lacked a distinctive rod-interrod architecture and showed patches of hypomineralization and locations where the outer enamel was incompletely formed (Fig. 2G). In contrast, *Dspp*^−1fs^ molar enamel more closely resembled that of the wild-type, while *Dspp*^−1fs^ molar dentin completely lacked dentinal tubules (Fig. 2H). Overall, the bSEM analyses showed that *Dspp*^P19L^ mouse teeth exhibited both dentin and enamel malformations (with minimal differences between *Dspp*^+/P19L^ and *Dspp*^P19L/P19L^ mice), while *Dspp*^−1fs^ teeth exhibited more severe dentin malformations compared to *Dspp*^P19L^ teeth (typically with minimal differences between *Dspp*^+/−1fs^ and *Dspp*^−1fs/−1fs^ mice), while the *Dspp*^−1fs^ molar enamel exhibited rod/interrod architecture and lacked the localized surface roughness observed in *Dspp*^P19L^ molars.

### Nanohardness of incisor enamel, dentin and nearby bone

**Figure 3:**
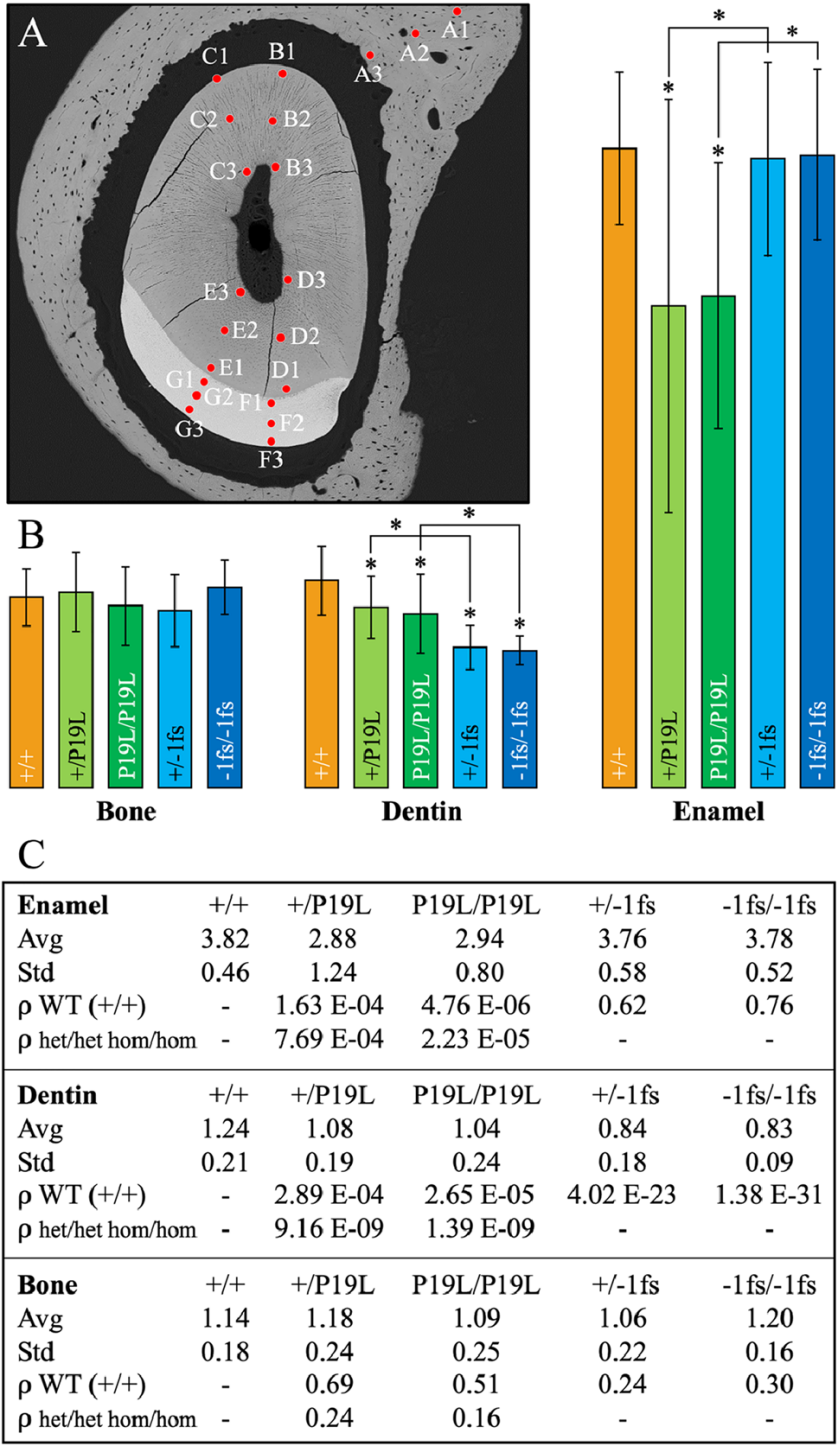
Nanohardness of incisor enamel, dentin and nearby bone in wild-type, *Dspp*^P19L^, and *Dspp*^−1fs^ Mice. (**A**) Red dots show that each sample is indented at 3 positions (A1–A3) in bone, 12 positions (B1–E3) in dentin, and 6 positions (F1–G3) in enamel. (**B**) Bar graph showing the results of nanohardness testing. Asterisks indicate statistical difference (α = 0.05). (**C**) Table showing the results of nanohardness testing in GPa. WT, wild-type; het, heterozygote; hom, homozygote; Avg, average; Std, standard deviation; ⍴(WT), ⍴-value as compared to wild-type; ⍴(het/het or hom/hom), p-value as comparing *Dspp*^+*/*P19L^ with *Dspp*^+*/*−1fs^ (het/het), or *Dspp*^P19L*/*P19L^ with *Dspp*^−1fs*/*−1fs^ (hom/hom).

We performed nanohardness testing on 7-week hemi-mandibles cross-sectioned at level 8 in wild-type, *Dspp*^P19L^, and *Dspp*^−1fs^ mice. Each sample was indented at 6 positions in enamel, 12 positions in dentin, and 3 in bone (Fig. 3A). The raw hardness data is provided in the supplemental data (enamel, Table S1; dentin, Table S2; bone, Table S3). No statistically significant nanohardness differences were observed among *Dspp*^+*/*+^ (GPa 3.82), *Dspp*^+*/*−1fs^ (GPa 3.76), and *Dspp*^−1fs*/*−1fs^ (GPa 3.78) incisor enamel (Fig. 3). However, the nanohardness of *Dspp*^+*/*P19L^ (GPa 2.88) and *Dspp*^P19L*/*P19L^ (GPa 2.94) enamel was significantly less than both the wild-type enamel and the corresponding *Dspp*^+*/*−1fs^ or *Dspp*^−1fs*/*−1fs^ enamel. Thus, enamel hardness was reduced in the *Dspp*^P19L^, but not in the *Dspp*^−1fs^ incisors, while the enamel nanohardness measurements of the *Dspp*^+*/*P19L^ (Std 1.24) and *Dspp*^P19L*/*P19L^ (Std 0.80) incisors were more variable than those of the wild-type (Std 0.46). The hardness data was consistent with the bSEM analyses, that the pathosis caused by expression of the *Dspp*^−1fs^ allele predominantly affected dentin, while expression of the *Dspp*^P19L^ allele was more damaging to the forming enamel.

Statistically significant nanohardness differences in dentin were observed between *Dspp*^+*/*+^ (GPa 1.24), and all the *Dspp* modified mice: *Dspp*^+*/*P19L^ (GPa 1.08), *Dspp*^P19L*/*P19L^ (GPa 1.04), *Dspp*^+*/*−1fs^ (GPa 0.84), and *Dspp*^−1fs*/*−1fs^ (GPa 0.8) mice (Fig. 3). However, the dentin nanohardness of the *Dspp*^*−*1fs^ incisors was also significantly reduced relative to the dentin nanohardness of the corresponding heterozygous or homozygous *Dspp*^P19L^ incisors. Thus, dentin nanohardness was reduced in the *Dspp*^P19L^ and *Dspp*^−1fs^ incisors, but the reduction was significantly greater in the *Dspp*^−1fs^ incisors. The bone hardness in the region near the incisor where it was measured showed no significant differences in any of the mice.

### Histology of developing incisors and molars

**Figure 4:**
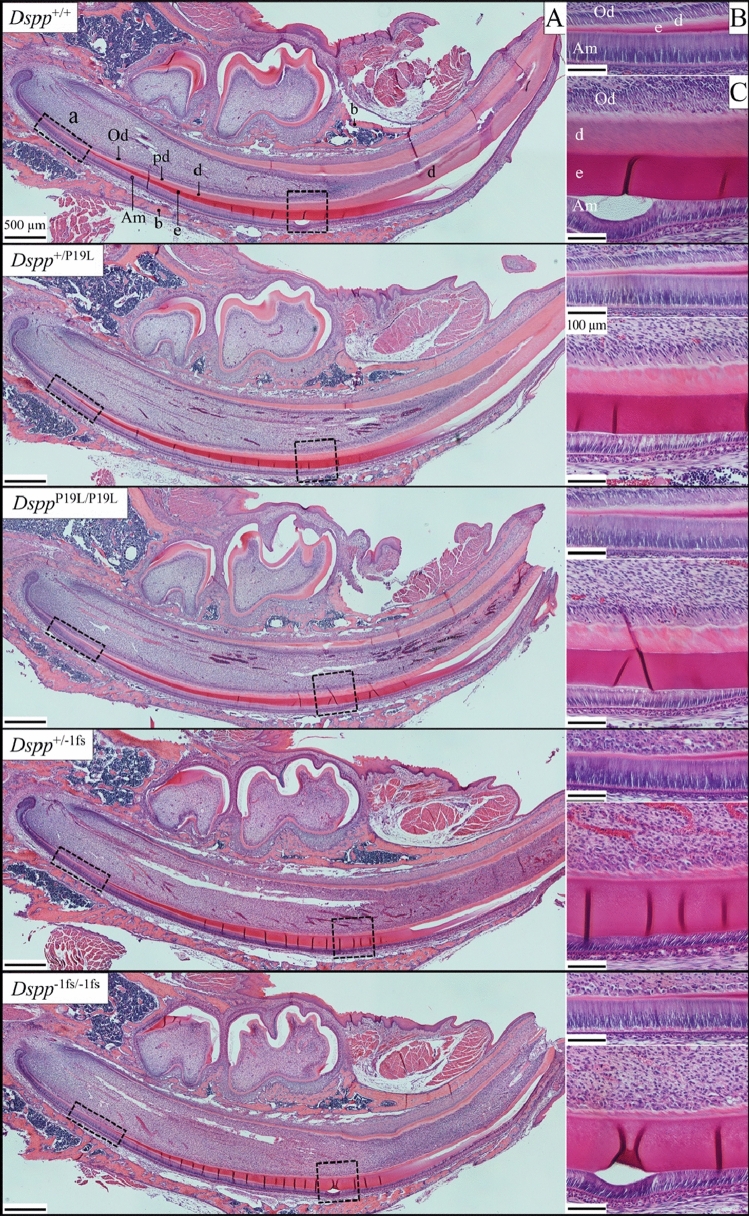
H&E staining of D14 Mandibular Incisors from *Dspp*^+/+^, *Dspp*^+/P19L^, *Dspp*^P19L/P19L^, *Dspp*^+/−1fs^ and *Dspp*^−1fs/−1fs^ Mice. (**A**) Longitudinal hemi-mandible sections with boxes showing the positions of higher magnification images in (**B**) and (**C**). (**B,C**) Note the irregular dentin mineralization front (at the predentin-dentin border) and uneven staining of the modestly thinner dentin layer in *Dspp*^P19L^ incisors, and odontoblast dysplasia, irregular dentin mineralization front, and very thin dentin in the *Dspp*^−1fs^ incisors. Key: *Am* ameloblasts, *b* bone, *d* dentin, *e* enamel, *Od* odontoblasts, *p* predentin. Scale bars in (**A**) 500 µm, in (**B,C**) 100 µm.

We first examined the histology of developing wild-type, *Dspp*^P19L^, and *Dspp*^−1fs^ incisors (Fig. 4). The *Dspp*^P19L^ incisors generally showed normal odontoblast and ameloblast morphology, but the dentin mineralization front (at the predentin-dentin border) was irregular. The dentin contained dentinal tubules but stained unevenly and was modestly thinner than wild-type dentin (Fig. 4). The *Dspp*^−1fs^ incisors showed normal ameloblast morphology, but odontoblast dysplasia, an irregular dentin mineralization front, and very thin dentin layer lacking dentinal tubules (Fig. 4, Suppl Fig. S17).

**Figure 5:**
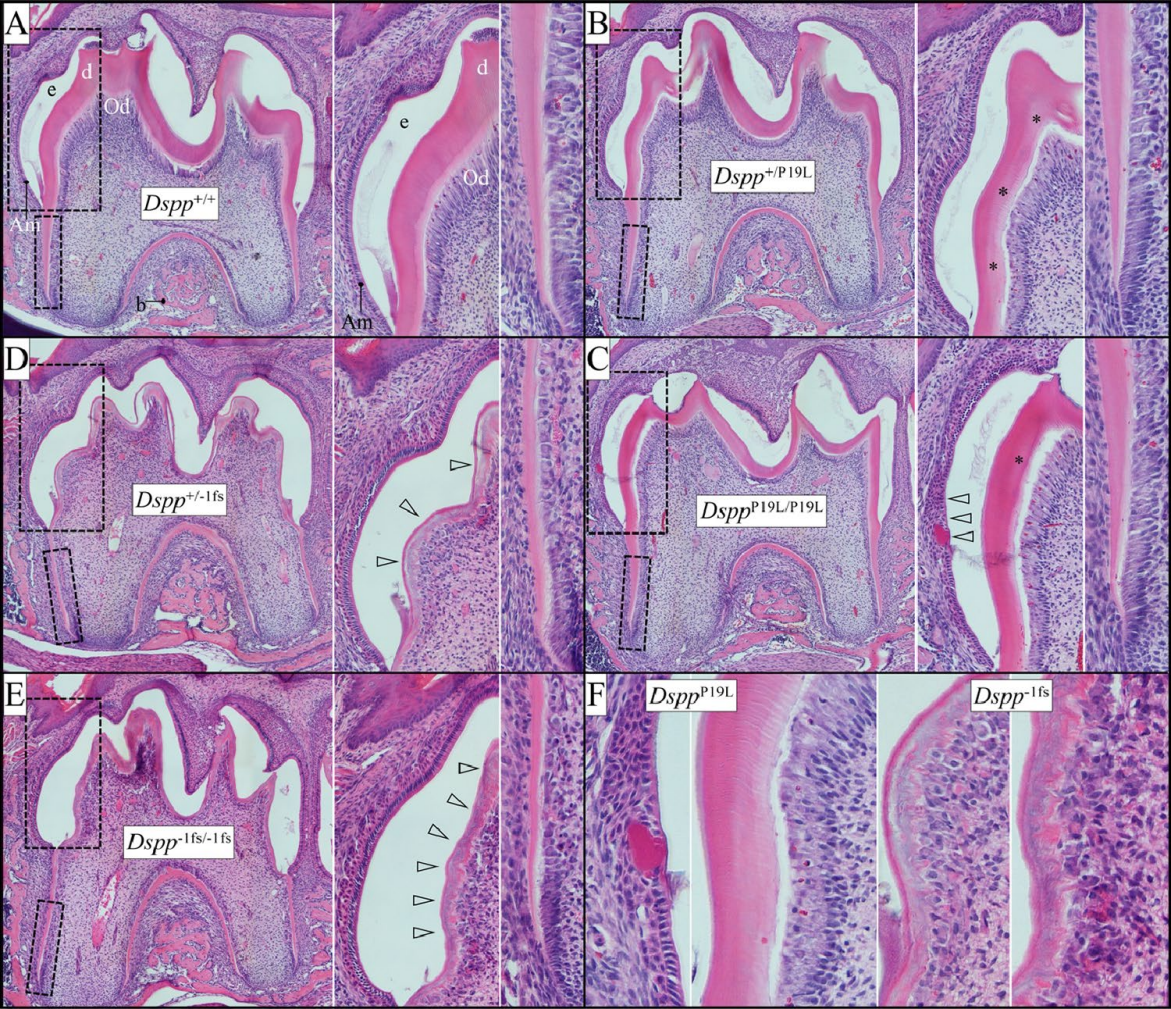
H&E staining of D14 Mandibular First Molars. (**A–E**) Longitudinal sections of first molars as they are about to erupt into the oral cavity are shown from *Dspp*^+/+^, *Dspp*^+/P19L^, *Dspp*^P19L/P19L^, *Dspp*^+/−1fs^ and *Dspp*^−1fs/−1fs^ mice, with boxes showing the positions of higher magnification images on the right. *Dspp*^+/P19L^ and *Dspp*^P19L/P19L^ molars show polarized columnar odontoblasts that form dentin with dentinal tubules, but the dentin stains unevenly (asterisks). Ameloblasts sometimes show patches of dysplasia associated with densely stained material (leftward pointing arrowheads). Odontoblasts in *Dspp*^+/−1fs^ and *Dspp*^−1fs/−1fs^ molars are dysplastic and their dentin, which totally lacks dentinal tubules, is very thin, stains unevenly (rightward pointing arrowheads), and contains islands of cellular debris. (**F**) Higher magnification views of *Dspp*^P19L^ ameloblast dysplasia and unevenly stained dentin with dentinal tubules formed by polarized odontoblasts (left) and *Dspp*^+/−1fs^ and *Dspp*^−1fs/−1fs^ odonotoblast dysplasia associated with a thin dentin layer lacking any dentinal tubules but containing cellular debris. Key: *Am* ameloblasts, *b* bone, *d* dentin, *e* enamel, *Od* odontoblasts, *p* predentin.

Next we examined the histology of developing wild-type, *Dspp*^P19L^, and *Dspp*^−1fs^ and D14 first molars (Fig. 5). As in the incisors, *Dspp*^P19L^ dentin contained dentinal tubules, but stained unevenly, suggesting variations in its degree of mineralization, and was modestly thinner (DEJ to pulp) relative to the wild-type. Unlike the incisors, the *Dspp*^P19L^ molars sometimes showed patches of ameloblast dysplasia that were associated with densely stained material.

**Figure 6:**
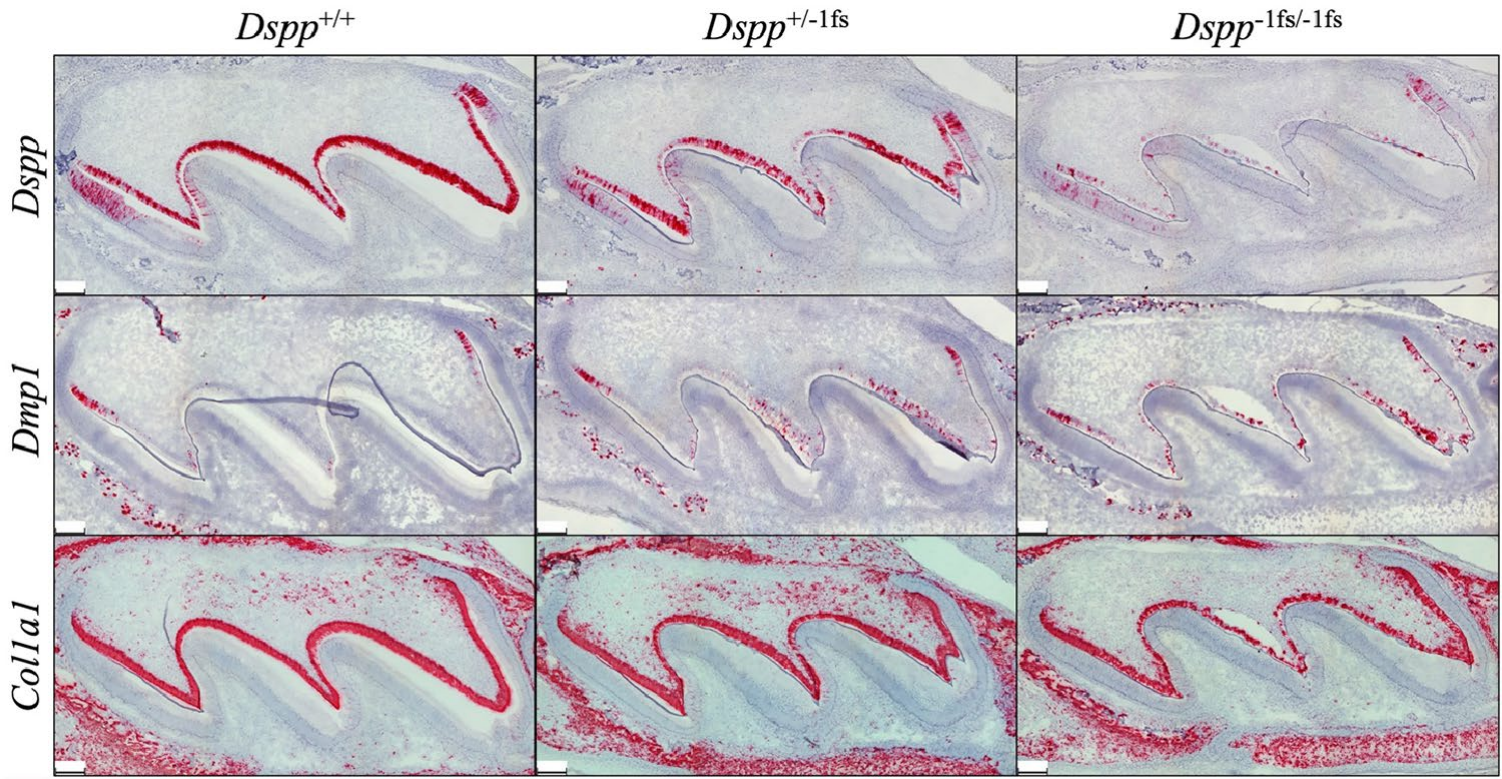
Expression of *Dspp, Dmp1,* and *Col1a1* mRNA in the wild-type, *Dspp*^+*/*−1fs^, and *Dspp*^−1fs*/*−1fs^ Mice. In situ hybridization/RNAscope is performed on 3-day-old *Dspp*^−1fs^ mouse maxillary 1^st^ molars to show mRNA in red. *Upper row* In the wild-type mice, *Dspp* mRNA is observed in the odontoblasts and presecretory and early secretory ameloblasts. *Dspp* mRNA in both *Dspp*^+/−1fs^ and *Dspp*^−1fs/−1fs^ mice are reduced in both odontoblasts and ameloblasts. The reduction is more apparent in the *Dspp*^−1fs/−1fs^ mice. *Middle row Dmp1* is mainly expressed at the early stage of dentin formation, and its expression is at a very low level soon after the onset of dentin mineralization. In the odontoblasts of the *Dspp*^+/−1fs^ and *Dspp*^−1fs/−1fs^ mice, *Dmp1* expression after the onset of dentin mineralization is elevated. *Lower row Col1a1* expression by the odontoblasts is mildly weaker in the *Dspp*^+/−1fs^ and *Dspp*^−1fs/−1fs^ mice. Scale bar: 100 μm.

We characterized the expression of *Dspp,* dentin matrix protein 1 (*Dmp1*)*,* and type I collagen (*Col1a1*) mRNA in the *Dspp*^+*/*+^, *Dspp*^+*/*−1fs^, and *Dspp*^−1fs*/*−1fs^ in day 3 (D3) mouse maxillary first molars by in situ hybridization (Fig. 6). In the wild-type, *Dspp* mRNA staining was observed in all odontoblasts outside of the cervical loop, but only in preameloblasts until the approximate onset of dentin mineralization^22^. *Dmp1* mRNA staining was observed only transiently, in early odontoblasts opposite the preameloblasts expressing *Dspp*, and was essentially silent during the bulk of dentin mineralization. *Dmp1* mRNA staining was never observed in ameloblasts. *Col1a1* mRNA staining was observed in all odontoblasts starting in the cervical loop and was never observed in ameloblasts.

The signal in odontoblasts from mRNA expressed by the *Dspp*^−1fs^ allele was less than that of the wild-type (*Dspp*^+*/*+^) allele, as *Dspp* mRNA staining was progressively reduced going from the *Dspp*^+*/*+^ to *Dspp*^+*/*−1fs^ to *Dspp*^−1fs*/*−1fs^ conditions (Fig. 6), suggesting that the *Dspp*^−1fs^ mRNA was selectively degraded or the cells expressing it deteriorated. The latter explanation is consistent with the absence of dentinal tubules. *Dmp1* mRNA in *Dspp*^−1fs^ mice was observed in early odontoblasts outside the cervical loop (as in the wild-type) but also in islands of odontoblasts where dentin was undergoing mineralization. *Col1a1* mRNA staining in odontoblasts was largely unchanged in *Dspp*^−1fs^ mice relative to the wild-type, except for some sporadic gaps in the odontoblast layer.

**Figure 7:**
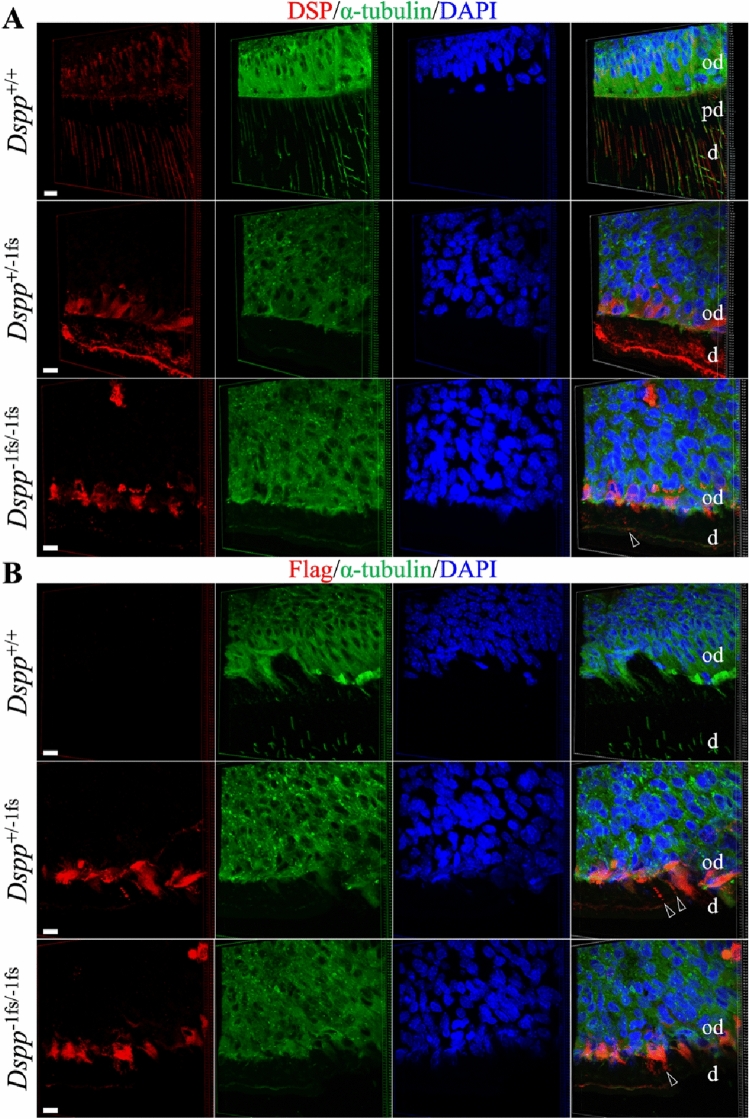
DSPP protein accumulated within the odontoblasts of the *Dspp*^+*/*−1fs^, and *Dspp*^−1fs*/*−1fs^ Mice. Immunohistochemistry is performed on the 14-day-old wild-type, *Dspp*^+*/*−1fs^, and *Dspp*^−1fs*/*−1fs^ mouse maxillary 1st molars. DSP (**A**) or Flag (**B**), α-tubulin (mapping the boundary of cells), and nucleus (DAPI) are shown in red, green, and blue, respectively. (**A**) Three-dimensional reconstruction of the DSP signal show that mild intracellular DSP signal, and moderate extracellular DSP signals follow the course of the odontoblastic processes in the wild-type mice. The predentin area is weak in DSP signal in the wild-type mice. In *Dspp*^+/−1fs^ and *Dspp*^−1fs/−1fs^ mice, elevated intracellular DSP signal is observed, and extracellular DSP scarcely aggregate in the abnormal dentin matrix (arrowhead). (**B**) Three-dimensional reconstruction of Flag signal show strong intracellular accumulation of mutant DSPP and dotted extracellular aggregates of mutant DSPP (arrowheads). Key: *od* odontoblast, *d* dentin, *pd* predentin. Scale bar: 10 μm.

To gain a better understanding of the fate of the frameshifted DSPP protein expressed from the *Dspp*^−1fs^ allele, we performed 3D immunohistochemistry on D14 maxillary molars using an antibody raised against the DSP (N-terminal DSPP domain) (Fig. 7A) and an anti-FLAG antibody used to detect the FLAG peptide translated from a sequence inserted into the *Dspp*^−1fs^ allele (Fig. 7B). D14 was selected to allow for sufficient deposition of dentin (which grew slowly and was very thin) in the *Dspp*^−1fs^ mice. The sections were also stained with α-tubulin to distinguish cellular from extracellular spaces. The predominant wild-type signal using the DSP antibody was in dentinal tubules. In contrast, the FLAG antibody showed strong intracellular signal for the − 1fs DSPP protein, with rare signal in the extracellular matrix that sometimes correlated with cellular (α-tubulin positive) debris. These findings are consistent with the interpretation that virtually all the frameshifted DSPP protein is retained in the cells and that the severe disturbance of dentin formation in *Dspp*^−1fs^ mice is caused by odontoblast pathosis, rather than a loss of DSPP function.

## Discussion

Evolutionary evidence indicates that an intact *Dspp* gene is only required for tooth formation. *Dspp* is truncated in two toothless anteater species^23^, and pseudogenized in edentulous Chinese pangolin, turtles, and birds^24^. In enamelless mammals (sloth, armadillo, and aardvark), the *Dspp* gene remains intact, suggesting that selection pressure for *Dspp* is limited to teeth^24^. Autosomal dominant inherited dentin defects resulting from mutations in *DSPP* are not caused by a loss of DSPP function. The dental phenotype is caused by the aberration in the DSPP protein itself, which can damage cells expressing DSPP and potentially interfere with wild-type DSPP protein secretion in heterozygotes (animals expressing both a normal and mutant *Dspp* allele). Immunohistochemistry in *Dspp*^P19L^^19^ and *Dspp*^−1fs^ D14 molars (Fig. 7) shows that little if any mutant DSPP protein is secreted by odontoblasts. These findings suggest that the direct effect of the mutant protein is cell pathosis that secondarily affects the mineralizing matrix. Throughout tooth development DSPP is strongly expressed by odontoblasts, and transiently expressed by preameloblasts^22^ (Fig. 6). DSPP protein was detected in bone at 1/400th the level observed in dentin^25^. As *Dspp* is expressed outside of tooth formation, other tissues could potentially experience pathosis by expressing a mutant DSPP protein.

Dentin is made by odontoblasts, ectomesenchymal cells of cranial neural crest origin^26^. Odontoblasts form a sheet of polarized columnar cells with their cell bodies forming the periphery of the dental pulp, while their cell processes project through predentin (the layer of unmineralized extracellular matrix between the odontoblast cell bodies and dentin mineral) and into dentin (via dentinal tubules). Some odontoblastic processes extend beyond the dentin-enamel junction (DEJ) and into the inner enamel (enamel spindles)^27^. Odontoblasts deposit primary dentin until the tooth erupts into occlusion, when the pace of dentin production slows, and secondary dentin is deposited. Dentin deposition continues until the dental pulp is essentially obliterated, typically in old age. Odontoblasts can increase the pace of dentin deposition as a reaction to dental caries or injury (including injuries caused by mutated proteins), forming tertiary dentin^28^. Sometimes newly differentiated odontoblasts bolster this response and deposit tubular or atubular dentin along with primary odontoblasts^29^. If the injury is severe enough to cause death of primary odontoblasts, pulp progenitor cells are recruited that differentiate into odontoblast-like cells that lack odontoblastic processes and deposit atubular reparative dentin^30^. Deposition of atubular dentin indicates that the primary odontoblasts have died^28^.

Tertiary dentin is classified as reactionary or reparative^31^. In reactionary dentin primary odontoblasts survive the injury and dentinal tubules are observed, while reparative dentin forms in response to a stronger stimulus that kills the primary odontoblasts and results in dentin lacking tubules. Odontoblasts respond differently to the modified DSPP protein in *Dspp*^P19L^ and *Dspp*^−1fs^ mice. Odontoblast pathosis is not as severe in *Dspp*^P19L^ mice and is characterized by the formation of reactionary dentin. Dentin mineralization is slower than normal and less mineralized, but the dentin contains numerous dentinal tubules, suggesting that many odontoblasts are long-lived and are not replaced by newly differentiated cells from the pulp. The *Dspp*^+/P19L^ dentin phenotype partially resembles that observed in *Dspp*^−/−^ mice^10,32^, suggesting that the *Dspp* 5' mutation might cause dominant dentin malformations, in part, by reducing the secretion of DSPP expressed from the wild-type allele, a concept that is consistent with in vitro findings^12^ and the observation that pig DSP forms covalent dimers^33^.

In *Dspp*^−1fs^ mice the injury to odontoblast is severe and reparative dentin forms, which requires the differentiation of new odontoblast-like cells derived from progenitor/stem cells recruited from the pulp^34^. This interpretation is consistent with the observation of *Dmp1* expression in places on the developing tooth where it has normally been turned off. The cells expressing *Dmp1* are newly differentiated odontoblast-like cells that are associated with the slow formation of reparative dentin (Fig. 6). DMP1 has been shown to stimulate the differentiation of odontoblasts and the expression of *Dspp *in vitro^35^ and in vivo^36^ and is used as a marker for newly formed dentin.

Our findings suggest that *Dspp*^P19L^ and *Dspp*^−1fs^ mice could serve as valuable mouse models for the formation of reactionary and reparative dentin, respectively. The response of odontoblasts to the types of *DSPP* mutations is probably not so clearly defined in patients. In teeth examined histologically from the Brandywine isolate, reactionary dentin tended to form under the cusps and reparative dentin laterally, which was thought to explain how the teeth acquired their bulbous shape^37^. Characterization of multiple mice analogous to other *DSPP* 5′ and 3′ disease-causing mutations will help determine if the distinctive dental malformations observed in *Dspp*^P19L^ and *Dspp*^−1fs^ mice generalize to those resulting from other 5′and 3′ mutations in DSPP, respectively.

A *Dspp*^P19L^ defect affecting protein targeting proves to be less toxic to odontoblasts than the *Dspp*^−1fs^ frameshift mutation, whereas the opposite is true with respect to ameloblasts. Dental enamel malformations are observed in *Dspp*^P19L^, but generally not in *Dspp*^−1fs^ mice. The *Dspp*^P19L^ enamel malformations were not uniform but manifested as patches of hypomineralized and hypoplastic enamel. This suggests that some, but not necessarily all, ameloblasts are damaged during their transient expression of *Dspp*^P19L^. Consequently, the enamel malformations are localized rather than generalized. There can be no doubt however, that the enamel malformations are a direct effect and result in hypoplastic and hypomineralized enamel of reduced hardness and are evident even prior to eruption of the tooth into the oral cavity. Variations in the severity of pathosis caused by expression of a genetically altered protein could be as simple as differences in mRNA degradation or protein translation, random interactions of cellular proteins with the altered protein or differences in ER stress responses.

True enamel defects occur in roughly a third of all cases of non-syndromic inherited dentin defects^38^. Many scientists believed that DGI-associated enamel malformations and rapid attrition were likely to be a secondary effect in all cases because the primary defect was in dentin, a mesodermal structure, and enamel, being an ectodermal structure, was unlikely to be dependent upon proteins typically expressed by mesodermal tissues^9^. Dental enamel often undergoes rapid attrition in patients with OI caused by defects in type I collagen, which is expressed by odontoblasts and not ameloblasts^39^. Other scientists observed teeth from affected persons in the Brandywine isolate that exhibited enamel that was generally or locally hypoplastic or pitted^37,40^ and underwent rapid attrition^41^. They believed that the enamel defects observed in DGI-III patients from the Brandywine isolate were caused directly by the genetic mutation and helped distinguish DGI-II and DGI-III as different disorders^40^.

A *DSPP* 5′mutation (c.49C > T; p.Pro17Ser)^39^ is known to be the cause of the severe tooth defects observed in the “Brandywine Isolate”^42^, which was specifically designated as DGI-III in the Shields classification^6^. At the present time the 19 different *DSPP* mutations that fall into the 5′ group are believed to affect trafficking of the DSPP protein. Direct effects on enamel have been demonstrated in the Brandywine isolate resulted from the *DSPP* p.(Pro17Ser) defect^39,43^. In addition, studies of patients with other 5′ *DSPP* mutations also demonstrated or suggested a direct enamel phenotype: p.(Pro17Leu)^17^, c.52-2A > G^43^, p.(Gln45His)^43^, and c.135 + 1G > A^14^. We are unaware of any reports demonstrating direct enamel malformations in the 29 reported *DSPP* -1 frameshift mutations, about half of which were diagnosed with the relatively mild DD-II phenotype.

In this study we compared dentin and enamel formed in mouse models for the two categories of gain-of-function/dominant negative *DSPP* mutations that are known to cause virtually all cases of DD-II, DGI-II, and DGI-III in humans. We know that preameloblasts and odontoblasts both express *Dspp*. In this study we demonstrate that these cells react differently to the two categories of mutant DSPP proteins. The *Dspp*^P19L^ mice exhibited malformations and decreased nanohardnesses of both dentin and enamel. The *Dspp*^−1fs^ mice exhibited severe dentin defects, analogous to reparative dentin where the primary odontoblasts die and are replaced, while the enamel was of normal hardness and architecture.

We believe these findings are clinically relevant. It has never been easy to apply the Shield’s classification, as the dentin may be thin early (shell teeth), but thick later (pulp obliteration)^42^. We propose that all 5′ *DSPP* mutations be classified clinically as DGI-III and should warn of an increased probability of enamel malformations contributing to the phenotype. A genetic diagnosis is straight-forward in cases of all 5′ *DSPP* mutations, whereas 3′ *DSPP* − 1 frameshift mutations often require special techniques to identify mutations within the extended highly repetitive DPP sequence^13,44^. Dental malformations caused by 3′ *DSPP* − 1 frameshift mutations should be classified as DGI-II for those with a more severe clinical phenotype, and DD-II when the clinical phenotype in the secondary dentition is mild. In all DGI-III cases and many DGI-II cases the dentist should consider intervention with stainless steel crowns to prevent rapid attrition and the loss of vertical dimension^45,46^.

## Methods

### Mice used in study

All animals used in this study were housed in Association for Assessment and Accreditation of Laboratory Animal Care International (AAALAC)-accredited facilities and were treated humanely based on protocols approved by the University of Michigan and Texas A&M Institutional Animal Care and Use Committees and were carried out in compliance with ARRIVE guidelines. Experimental protocols were designed along University and National Institutes of Health (NIH) guidelines for the humane use of animals.

### Generation of the ***Dspp***^−1fs^ mouse model

The *Dspp*^−1fs^ mouse model was designed and produced by the University of Michigan Transgenic Animal Model Core using the CRISPR/Cas9 approach. The targeting strategy, experimental details, breeding scheme, and genotyping protocols were described in the supplementary data (Figs. S1–S3). Along the process of validating the correctly targeted *Dspp*^−1fs^ mouse line, an allele with *Dspp*^−DPP^ was identified, characterized, and preserved as a separate line that carries only the *Dspp*^−DPP^ mutation. Mice were maintained on rodent lab chow and supplemented with DietGel (ClearH_2_O, Westbrook, ME). G7 littermates were collected for characterization. Both mouse models, *Dspp*^−1fs^ (ID 67179) and *Dspp*^−DPP^ (ID 67180) are available at Mutant Mouse Resources & Research Centers (MMRRC), University of North Carolina Facility.

### Morphological assessment

Seven-week-old mice were anesthetized with isoflurane, sacrificed, and perfused with 4% paraformaldehyde (PFA) for 10 min. Their mandibles were denuded of soft tissues, post-fixed by immersion in 4% PFA overnight, and rinsed with phosphate-buffered saline (PBS) three times, for 5 min each. The teeth were cleaned with 1% bleach (sodium hypochlorite), rinsed with PBS, air dried, displayed on the Nikon SMZ1000 dissection microscope, and photographed using a Nikon DXM1200 digital camera.

### Backscattered scanning electronic microscopy (bSEM)

The bSEM procedures were described previously^47^. Seven-week-old and 14-day-old mice were anesthetized using isoflurane and perfused with 4% PFA, and hemi-mandibles were dissected free of soft tissue. The hemi-mandibles were dehydrated using an acetone series (30%, 50%, 70%, 80%, 90%, 100%), embedded in epoxy, cross-sectioned at 1 mm increments along their lengths, and characterized by bSEM at each level. Samples were mounted on metallic stubs using conductive carbon cement and sputter-coated with an Au–Pd film to increase conductivity. An Amray EF 1910 Scanning Electron Microscope operating at an accelerating voltage of 5 kV was used to image the samples. SEM imaging was conducted at the Michigan Center for Materials Characterization (Ann Arbor, MI).

### Nanohardness testing

Hemi-mandibles from *Dspp*^+/+^, *Dspp*^+/−1fs^, *Dspp*^−1fs/−1fs^, *Dspp*^+/P19L^, and *Dspp*^P19L/P19L^ mice were collected at 7 weeks and characterized by nanohardness testing as described previously^48^. In brief, left and right hemimandibles were dissected free of soft tissue, dehydrated with an acetone series, embedded in epoxy, cut transversely at the level of the labial alveolar crest (Level 8) and re-embedded in Castolite AC in 25-mm SeriForm molds (Struers Inc., Westlake, OH). The incisor cross sections were successively polished using a series of waterproof silicon carbide papers and then 1-micron diamond paste. Nanohardness testing was performed using a Hysitron 950 Triboindenter with a nanoDMA transducer and Berkovich probe, and the nanoindentations analyzed using Triboscan 9 software (University of Michigan Center for Materials Characterization).

### Histological analysis

Heads of 3-day-old (D3) and 14-day-old (D14) mice were harvested, fixed in 4% PFA in PBS at 4 °C overnight. The samples were decalcified at 4 °C in 4.13% disodium ethylenediaminetetraacetic acid (EDTA, pH 7.4) with agitation for 4 days (D3 samples) or 12 days (D14 samples). The samples were then dehydrated by an ethanol series, cleared by xylene, embedded in paraffin, and sectioned at 5 μm thickness. Sections were obtained from maxillary and mandibular molars and mandibular incisors and placed on Fisherbrand Tissue Path Superfrost Plus Gold Microscope Slides (Fisher Scientific) for histological analysis. Hematoxylin and Eosin (H&E) staining was performed, and images were taken using a Nikon Eclipse TE300 microscope and photographed using a Nikon DXM1200 digital camera as described previously^49^.

### In situ hybridization

In situ hybridization was performed using RNAscope^®^ 2.5 Assay with RED HD Detection Reagent, following the user manual 322452 and 322360 provided by Advanced Cell Diagnostics (Newark, CA). Specifically, target retrieval was performed in a sealed container that was further placed in boiling water for 30 min; sections were incubated in RNAscope^®^ Protease Plus for 30 min at 40 °C; hybridization was performed for 2 h at 40 °C; signal detection was conducted for 4 min. The following probes were used: (1) Mm-Dspp-O1 (Cat #576161, targeting NM_010080.3, nt 128–1755, skipping nt 1431–1460); (2) Mm-Dmp1 (Cat #441171, targeting NM_016779.2, nt 689–1543); and (3) Mm-Col1a1 (Cat #319371, targeting NM_007742.3, nt 1686–4669). Images were taken as described in “Histological analysis” section.

### Immunohistochemistry

Paraffin sections were heated at 60 °C for 1 h, rehydrated in xylene then an ethanol series. Antigen retrieval was performed in Tris–EDTA buffer (10 mM Tris Base, 1 mM EDTA, 0.05% Tween 20, pH 9.0). The antigen retrieval container was placed in boiling water for 25 min, followed by cooling down at room temperature (RT) for 25 min. Sections were then blocked in 3% bovine serum albumin, 10% normal goat serum, 0.05% Tween 20 in PBS at RT for 2 h, and incubated in an unconjugated rabbit primary antibody in antibody dilution buffer (10% normal goat serum, 0.05% Tween 20 in PBS) at RT for 1 h, then at 4 °C overnight. The unconjugated primary antibodies used in this study were a rabbit polyclonal antibody raised against the DSP protein^50^ diluted 1:2000 and a rabbit polyclonal anti-peptide (DYKDDDDK) antibody (Invitrogen, Waltham, MA; #PA1-984B) diluted 1:1000. A secondary antibody, Goat anti‐rabbit IgG(H + L) Secondary Antibody, Alexa Fluor Plus 555 (Invitrogen, Waltham, MA; #A32732), was used at a concentration of 1:1000 for incubation of 1 h at RT. Then, a mouse monoclonal antibody against alpha-tubulin with an AF488 conjugate (abcam, Cambridge, UK; #ab195887) diluted 1:100 was incubated for 1 h at RT. The section was counterstained in 4′,6-Diamidino-2-phenylindole dihydrochloride (DAPI, 1 μg/mL in distilled water; D9542, Sigma) for 2 min, then mounted in Invitrogen ProLong Gold Antifade Mountant with DAPI (P36935, Invitrogen, Carlsbad, CA). Images were taken using a Nikon A1 confocal combined with an inverted Ti-E microscope at the Imaging Laboratory of the Michigan Diabetes Research Center (Ann Arbor, MI). Z-stack images were taken in a series of 0.4 μm Z-steps for about 35 Z-steps to cover dentin matrix that may not firmly attached to the slides. 3D reconstruction of z-stack images were created using the built-in volume view function.

## Supplementary Information

Supplementary Information.
