# Supplementary material for: Mouse Dspp frameshift model of human dentinogenesis imperfecta
Source: Sci Rep. 2021 Oct 19;11:20653. doi: 10.1038/s41598-021-00219-4 (PMC8526674; doi:10.1038/s41598-021-00219-4)
Supplement: Supplementary file 1 — Supplementary Information. [file 41598_2021_219_MOESM1_ESM.pdf]

Mouse *Dspp* Frameshift Model of Human *Dentinogenesis Imperfecta*

Tian Liang[1], Yuanyuan Hu[1], Hong Zhang[1], Qian Xu[3], Charles E. Smith[1,2], Chuhua Zhang[1], Jung-Wook Kim[4,5], Shih-Kai Wang[6], Thomas L. Saunders[7], Yongbo Lu[3], Jan C.-C. Hu[1], James P. Simmer[1*]

[1]Department of Biologic and Materials Sciences, University of Michigan School of Dentistry, 1011 North University, Ann Arbor, MI 48108.

[2]Facility for Electron Microscopy Research, Department of Anatomy and Cell Biology and Faculty of Dentistry, McGill University, Montreal, Quebec H3A 2B2, Canada.

[3]Department of Biomedical Sciences and Center for Craniofacial Research and Diagnosis, Texas A&M University College of Dentistry, 3302 Gaston Ave., Dallas, TX 75246.

[4]Department of Molecular Genetics, School of Dentistry & Dental Research Institute, Seoul National University, Seoul, Korea.

[5]Department of Pediatric Dentistry, School of Dentistry & Dental Research Institute, Seoul National University, Seoul, Korea.

[6]Department of Dentistry, National Taiwan University School of Dentistry, No.1, Changde St., Jhongjheng District, Taipei City 100, Taiwan.

[7]Department of Internal Medicine, University of Michigan Medical School, 1150 West Medical Center Drive, MSRBII Room 2570 Ann Arbor, MI 41809

Supplemental Data

Supplemental Methods

Fig. S1. *Dspp* Sequence Modifications in the *Dspp*$^{-1fs}$ Allele.

Fig. S2. Coding and Deduced Amino Acid Sequences of the Mouse *Dspp*$^{-1fs}$, *Dspp*$^{+}$ (wild-type), and *Dspp*$^{-DPP}$ Alleles.

Fig. S3. Genotyping *Dspp*$^{+}$, *Dspp*$^{-1fs}$, and *Dspp*$^{-DPP}$ Offspring.

Fig. S4. Dissection Microscopy Photographs Comparing 7-week *Dspp*$^{+/+}$, *Dspp*$^{+/P19L}$, and *Dspp*$^{P19L/P19L}$ Mice.

Fig. S5. bSEM of a 7-Week *Dspp*$^{+/+}$ Mouse Mandibular Incisor Cross-Sectioned at 1 mm Increments.

Fig. S6ab. bSEM of a 7-Week *Dspp*$^{+/P19L}$ Mouse Mandibular Incisor Cross-Sectioned at 1 mm Increments.

Fig. S7ab. bSEM of a 7-Week *Dspp*$^{P19L/P19L}$ Mouse Mandibular Incisor Cross-Sectioned at 1 mm Increments.

Fig. S8ab. bSEM of a 7-Week *Dspp*$^{+/-1fs}$ Mouse Mandibular Incisor Cross-Sectioned at 1 mm Increments.

Fig. S9ab. bSEM of a 7-Week *Dspp*$^{-1fs/-1fs}$ Mouse Mandibular Incisor Cross-Sectioned at 1 mm Increments.

Fig. S10. bSEM of Day 14 *Dspp*$^{+/+}$, *Dspp*$^{+/-1fs}$, *Dspp*$^{-1fs/-1fs}$, *Dspp*$^{+/P19L}$, and *Dspp*$^{P19L/P19L}$ Mouse Mandibular Molars.

Fig. S11 bSEM of D14 *Dspp*$^{+/+}$ Mouse Mandibular Molars.

Fig. S12. bSEM of D14 *Dspp*$^{+/P19L}$ Mouse Mandibular Molars.

Fig. S13. bSEM of D14 *Dspp*$^{P19L/P19L}$ Mouse Mandibular Molars.

Fig. S14ab. bSEM of D14 *Dspp*$^{+/-1fs}$ Mouse Mandibular Molars.

Fig. S15a-d. bSEM of D14 *Dspp*$^{-1fs/-1fs}$ Mouse Mandibular Molars.

Fig. S16. bSEM of a 7-Week *Dspp*$^{+/-1fs}$ and *Dspp*$^{-1fs/-1fs}$ Mouse Mandibular Molars.

Fig. S17. Histology of D14 Mandibular Incisors, D3 and D14 Maxillary Molars of Wild-Type, *Dspp*$^{+/-1fs}$ and *Dspp*$^{-1fs/-1fs}$ Mice.

Table S1. Raw Data for Enamel Hardness Testing.

Table S2. Raw Data for Dentin Hardness Testing.

Table S3. Raw Data for Bone Hardness Testing.

References

Supplemental Methods

Generation of the *DSPP* frameshift mouse models. Mouse *Dspp* coding sequence (Chromosome 5: 104,170,712-104,180,127 forward strand) was analyzed, DNA oligo donor, and guide RNA sequences were designed, and genetically *Dspp* modified mouse lines were developed using CRISPR/Cas9 technology [1-3]. Two single guide RNAs (sgRNA) were designed to target the *Dspp* coding strand using an algorithm[4], C72G1 (5'AAGAGCAGCGACGAATCTAACGG3') and C72G2: (5'TTCGATGACGAGTCCATGCAAGG-3', synthesized, and cloned into the plasmid pX330 (Addgene.org plasmid #42230, a kind gift from Feng Zhang)[5].

Dspp targeting by the C72G1 and C72G2 was tested by co-electroporation of a PGKpuro plasmid[6] with each pX330 plasmid into JM8A3 mouse ES cells[7], and the cells were subjected to transient puromycin selection. Genomic DNA was prepared from the surviving cells, and a DNA fragment spanning the expected Cas9 cut sites was PCR amplified. The PCR amplicon was digested with T7 endonuclease I as described[8]. The presence of indel mutations produced by nonhomologous end joining repair of Cas9 induced double strand chromosome breaks was evident as lower molecular weight DNA fragments following T7 endonuclease I digestion (data not shown). Indel mutations were also evident as superimposed sequences in sequence chromatograms of the PCR products following the expected Cas9 cut sites. Both C72G1 and C72G2 induced indel mutations, as observed following T7 endonuclease I digestion. More of the products from C72G2 appeared to be sensitive to T7 endonuclease I digestion than those from C72G1. This suggested C72G2 was more active to induce indel mutations. Induction of indel mutations by both constructs were seen in the sequence chromatograms. Peaks-on-peaks were more prominent for construct C72G2 than for C72G1, indicating that construct C72G2 was more active to induce indel mutations. A donor oligonucleotide to introduce the desired knockin sequence by homology directed repair of the chromosome break was synthesized.

The circular pX330 plasmid containing the active C72G2 and the DNA donor (5 ng/μL final concentration plasmid and 10 ng/μL oligonucleotide donor) were used for pronuclear microinjection of mouse zygotes. Two separate rounds of microinjection of 250 eggs obtained from mating (C57BL/6J X SJL/J) F1 females with (C57BL/6J X SJL/J) F1 males produced 24 viable offspring. The modifications introduced into *Dspp* to establish a Flag sequence for specific and sensitive antibody detections, generate a -1 frameshift, and add six silent nucleotide modifications to prevent further Cas9 mediated editing of the donor molecule following incorporation into the genome are illustrated in Fig. S1. Sanger sequencing results of the coding regions from the $Dspp^{-1fs}$ (Fig. S2A), $Dspp^{+}$ (wild-type) (Fig. S2B), and $Dspp^{-DPP}$ (Fig. S2C) mice are shown in Fig. S2.

Genotyping was initially conducted with two PCR primer pairs: DSPP-WT-F 5'ATGCAAGGAGATGATCCCAA; DSPP-WT-R 5ATGAGTCATCACTGCTATCGTCTT; (184 bp product) DSPP-Shift-F 5'TACAAAGACGATGACGA CAAGG; DSPP-Shift-R 5'CTGCTGTCTGATTTGCTCTCA (337 bp product) (Fig. S3A).

Breeding the modified *Dspp* into the C57BL/6 background. The *Dspp* coding sequence of the $Dspp^{-1fs}$ and $Dspp^{-DPP}$ founders were confirmed with Sanger sequencing. Then both mice were crossed with C57BL/6N for seven generations to dilute any possible off-target effects of the CRISPR/Cas9 gene editing and to generate offspring for characterization. None of the founders or their offspring had observable changes in behavior or apparent physical defects from neonatal stage to adulthood. The average litter contained 4-6 pups. *Dspp* coding exons and intron borders of the F1 and G2 offspring were characterized by Sanger sequencing and no additional sequence variations were identified. Outbreeding of G2 through G7 mice with C57BL6 mice was performed to achieve a homogenous genetic background.

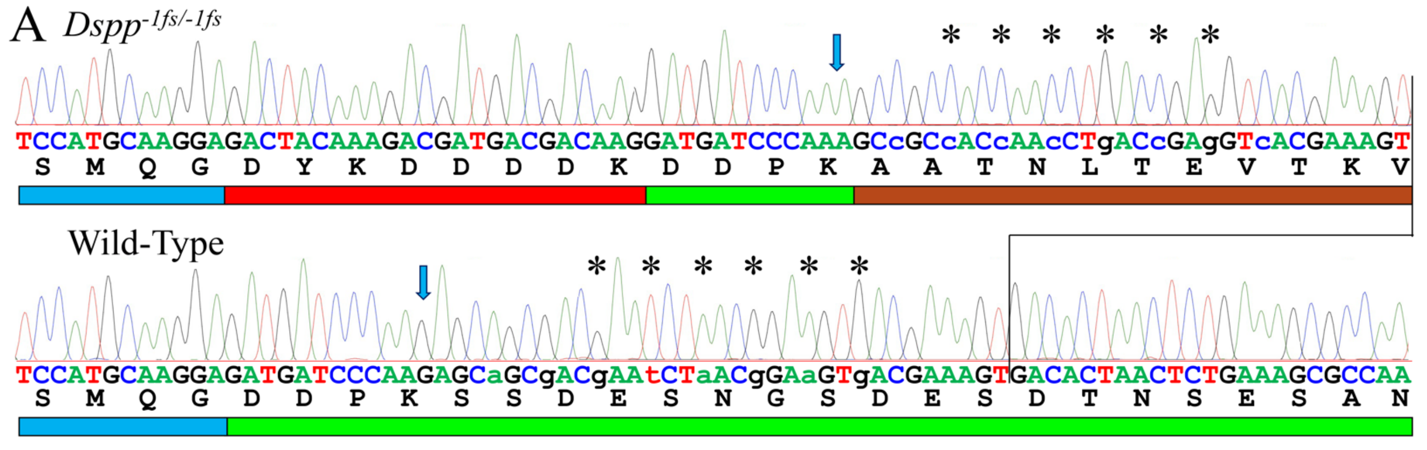

B

Translation of Mouse -1 Frameshift sequence

SMQGDYKDDDDKDDPKAATNLTEVTKVTLTLKAPMRVAAVEMLLTHLMNQVMMTMTVTHMREKTIAVMTHLVM
VTVTVMVMVTARVRTRTNLTAVTMTTAVTVRANQTAVTVVTTAVTAATVVTAVTAVTVVTVVTAATAVTAATA
TVVVTAATAAVVVTAATAVTPVTAVTAAIAVTAVTAVTAAIAVTAVTVVTAVTAATAAVVVTAATAAVVVTAA
TAVTAVTAAIAVTAVTAAAATAAAVATAVTVVTAVTAAAAATAATAVTVVTAVTVVAAVTAATVVPAATAAVV
VTAATAVVVVTAVTVVTVVTAVIAVRAATAVTAVTAATVVTAVTVATAATVVTVATAVTVATVATAVTAVTAA
TVVTAATVVTAVTVATVVTAVTAVTAATVVTAVTVVTAATVVTAVTAVTAVTAATAATAVTAATAATAVTAAT
AATAVTAVTVTARIAVLTAVMVTASLVMATVTATVTATVTVTVTVKAVTVTTQPVMIRSERTHDILCVTSW*

Translation of Mouse Wild-Type DPP sequence

SMQGDDPKSSDESNGSDESDTNSESANESGSRGDASYTSDESSDDDNDSDSHAGEDDSSDDSSGDGDSDSNGD
GDSESEDKDESDSSDHDNSSDSESKSDSSDSSDDSSDSSDSSDSSDSSDSSDSSDSSDSSDSNSSSDSSD
SSGSSDSSDSSDTCDSSDSSDSSDSSDSSDSSDSSDSSDSSDSSDSSSSSDSSDSSSCSDSSDSSDSSDS
SDSSDSSDSSSSDSSSSSNSSDSSDSSDSSSSSDSSDSSDSSDSSDSSGSSDSSDSSASSDSSSSSDSSDSSS
SSDSSDSSDSSDSSDSSESSDSSNSSDSSDSSDSSDSSDSSDSSDSSDSSDSSNSSDSSDSSDSSDSSDSSNS
SDSSDSSDSSDSSDSSDSSDSSDSSDSSDSSDSSDSSDSSDSSDSSDSSDSSDSSDSSDSSDSSDSSDSSNSSDSS
DSDSKDSSSDSSDGDSKSGNGNSDSNSDSNSDSDSDSEGSDSNHSTSDD*

Figure S1. *Dspp* Sequence Modifications in the *Dspp*$^{-1fs}$ Allele. ***A:*** Chromatograms of modified (*Dspp*$^{-1fs}$) and wild-type *Dspp* alleles. Nucleotide and deduced amino acid sequences, and colored bars representing protein structural elements are presented below the chromatograms. ***Key:*** Blue, dentin sialoprotein (DSP) C-terminus; Red, in-frame insertion of a Flag (DYKDDDDK) tag between DSP and DPP (dentin phosphoprotein); Green, DPP sequence; Brown, -1 frameshift sequence. Blue arrow, position of the "G" deleted to cause a -1 frameshift following the first four amino acids of DPP; asterisks mark positions of silent (relative to the shifted reading frame) nucleotide changes, which are also indicated in lower case in the nucleotide sequences. These substitutions were introduced to prevent further Cas9 mediated editing of the donor molecule following its incorporation into the genome. ***B:*** The deduced amino acid sequences of *Dspp*$^{-1fs}$ and wild-type *Dspp* alleles starting from around the DSP-DPP cleavage site. The context of the cleavage site (after SMQG) between the DSP and DPP proteins is interrupted by the inserted Flag tag (DYKDDDDK). Note that -1 frameshifted sequence does not hit an in-frame translation termination codon until after the WT termination codon and makes a longer protein. The highly repetitive DPP sequence is rich in serine (S, often phosphorylated) and aspartic acid (D), whereas the *Dspp*$^{-1fs}$ repetitive sequence is rich in the small hydrophobic amino acids alanine (A) and valine (V).

Figure S2. Coding and Deduced Amino Acid Sequences of the Mouse *Dspp*$^{-1fs}$, *Dspp*$^{+}$ (wild-type), and *Dspp*$^{-DPP}$ Alleles.

A) *Dspp*$^{-1fs}$ coding region. The mouse *Dspp*$^{-1fs}$ coding region starting at the translation initiation codon **atg** and extending beyond the natural translation termination codon (**tag**) to a more 3' termination codon (**tga**) in the -1 reading frame is shown. The introduced Flag coding sequence (5'**GACTACAAAGACGATGACGACAAG**), the deleted nucleotide to shift the reading frame along with the silent nucleotide changes required by the CRISPR design are indicated by bold capital letters (5'agc**C**gc**C**ac**C**aa**C**ct**G**ac**C**ga**G**gt**C**). The alternating green and yellow highlighting distinguishes *Dspp* exons 2-5.

atgaaaatgaagataattatatatatatgcatttgggcaactgcctgggccattccggttccccagttagtaccactggaaagagacattgttgaaaac
tctgtggctgtgcctcttctaacacatccaggaactgcagcacagaatgagttatctatcaacagcaccactagcaacagcaacgactccccagatggc
agtgagataggagagcaggtacttagcgaggatggttacaaaagagatgggaatggctccgagtcaatacatgtaggagggaaggattttcctactcag
cccatttttagtaaacgaacaggggaacactgctgaagaacacaatgacatagaaacatacggtcatgatggggtacatgcgagaggagagaacagcaca
gcaaatggcatcaggagccaggtaggcatcgttgaaaatgcggaggaagcagagagcagtgtccacggacaggctggtcagaatacaaaatctggaggt
gctagtgatgtaagccagaatggagatgcgacccttgtccaggaaaatgagcctccagaagctagcatcaagaatagcaccaaccatgaggctggaata
cacgggagtggggttgctacacatgaaacgacgcctcagagagaagggctggggagtgagaaccaggggactgaggtgacaccaagcatcggggaagat
gcaggtttggatgatactgatgggagtcctagcgggaacggggtagaggaggatgaagatacaggctctggtgatggtgagggtgcagaagcaggagat
ggaagggagagccatgatggcactaagggccagggggccaatctcatgggggaaacactgaccacagaggtcagagttcagttagtactgaagatgat
gattctaaagaacaagaaggcttcccaaatggacacaatggagacaacagcagtgaggaaaacggtgttgaagaaggcgacagtacccaggcaacgcag
gacaaccaaaagctcagccccaaagacacccgagatgcagagggtgggatcatcagccagtcagaagcatgtccttctgggaagagccaagatcaggga
atagaaactgaaggtcccaacaaaggcaacaaaagtattattaccaaagaatctgggaaactcagtggaagtaaagatagcaatggacaccaaggagtg
gagctggacaaaaggaatagcccaaagcaaggggagtctgacaagcctcaaggcactgctgagaaatcagctgcccacagtaacctgggacacagcagg
ataggtagcagcagcaatagtgatgggcatgacagttacgagttcgatgacgagtccatgcaagga**GACTACAAAGACGATGACGACAAG**gatgatccc
aa**-**agc**C**gc**C**ac**C**aa**C**ct**G**ac**C**ga**G**gt**C**acgaaagtgacactaactctgaaagcgccaatgagagtggcagccgtggagatgcttcttacacatctgat
gaatcaagtgatgatgacaatgacagtgactcacatgcgggagaagacgatagcagtgatgactcatctgatacagatgacagtgacagtaatggtgat
ggtgacagtgacagtaatggtgatggtgacagcgagagtgaggacaaggacgaatctgacagcagtgaccatgacaacagcagtgacagtgagagcaaa
tcagacagcagtgacagtagtgacgacagcagtgacagcagcgacagtagtgacagcagtgacagcagtgacagtagtgacagtagtgacagcagcgac
agcagtgacagcagcgacagcaacagtagtagtgacagcagcgacagcagcagtagtagtgacagcagcgacagcagtgacagcagtgacagcagtgac
agcagcgatagcagtgacagcagtgacagcagtgacagcagcgatagcagtgacagcagtgacagtagtgacagcagtgacagcagcgacagcagcagt
agtagtgacagcagcgacagcagcagtagtagtgacagcagcgacagcagtgacagcagtgacagcagcgatagcagtgacagcagtgacagcagcagc
agcgacagcagcagcagtagcaacagcagtgacagtagtgacagcagtgacagcagcagcagcagcgacagcagcaacagcagcgacagcagtgacagt
agtgacagcagtgacagtagtgacagcagtgacagcagcaacagtagtgacagcagcgacagcagcagtagtagtgacagcagcgacagcagcagtagt
agtgacagcagtgacagtagtgacagtagtgacagcagtgagagcagtgagagcagcgacagcagcaacagcagtgacagcagcgacagtagtgacagc
agtgacagtagcgacagcagcgacagtagtgacagtagcgacagcagtgacagtagcaacagtagcgacagcagtgacagcagtgacagcagcgacagt
agtgacagcagcaacagtagtgacagcagtgacagtagcgacagtagtgacagcagtgacagcagtgacagcagcgacagtagtgacagcagtgacagt
agtgacagcagcgacagtagtgacagcagtgacagcagtgacagcagtgacagcagcgacagcagcgacagcagtgacagcagcgacagcagcgacagc
agtgacagcagcgacagcagcaacagcagtgacagcagtgacagtgacagcaaggatagcagttctgacagcagtgatggtgacagcaagtctggtaat
ggcaacagtgacagcaacagtgacagcaacagtgacagtgacagtgacagtgaaggcagtgacagtaaccactcaaccagtgatgat**tag**atcagagag
aacccatgatatcctctgtgtgacctcttgg**tga**

MKMKIIIYICIWATAWAIPVPQLVPLERDIVENSVAVPLLTHPGTAAQNELSINSTTSNSNDSPDGSEIGEQVLSEDGYKRDGNGSESIHVGGKDFPTQ
PILVNEQGNTAEEHNDIETYGHDGVHARGENSTANGIRSQVGIVENAEEAESSVHGQAGQNTKSGGASDVSQNGDATLVQENEPPEASIKNSTNHEAGI
HGSGVATHETTPQREGLGSENQGTEVTPSIGEDAGLDDTDGSPSGNGVEEDEDTGSGDGEGAEAGDGRESHDGTKGQGGQSHGGNTDHRGQSSVSTEDD
DSKEQEGFPNGHNGDNSSEENGVEEGDSTQATQDNQKLSPKDTRDAEGGIISQSEACPSGKSQDQGIETEGPNKGNKSIITKESGKLSGSKDSNGHQGV
ELDKRNSPKQGESDKPQGTAEKSAAHSNLGHSRIGSSSNSDGHDSYEFDDESMQG**DYKDDDDK**DDPK**AATNLTEVTKVTLTLKAPMRVAAVEMLLTHLM**
NQVMMTMTVTHMREKTIAVMTHLIQMTVTVMVMVTVTVMVMVTARVRTRTNLTAVTMTTAVTVRANQTAVTVVTTAVTAATVVTAVTAVTVVTVVTAAT
AVTAATATVVVTAATAAVVVTAATAVTAVTAVTAAIAVTAVTAVTAAIAVTAVTVVTAVTAATAAVVVTAATAAVVVTAATAVTAVTAAIAVTAVTAAA
ATAAAVATAVTVVTAVTAAAAATAATAATAVTVVTAVTVVTAVTAATVVTAATAAVVVTAATAAVVVTAVTVVTVVTAVRAVRAATAATAVTAATVVTA
VTVATAATVVTVATAVTVATVATAVTAVTAATVVTAATVVTAVTVATVVTAVTAVTAATVVTAVTVVTAATVVTAVTAVTAVTAATAATAVTAATAATA
VTAATAATAVTAVTVTARIAVLTAVMVTASLVMATVTATVTATVTVTVTVKAVTVTTQPVMIRSERTHDILCVTSW*

B) *Dspp*$^{+}$ wild-type coding region. The mouse *Dspp*$^{+}$ wild-type coding region starting at the translation initiation codon **atg** and ending at the natural translation termination codon (**tag**) is shown. The alternating green and yellow highlighting distinguishes *Dspp* exons 2-5.

atgaaaatgaagataattatatatatatgcatttgggcaactgcctgggccattccggttccccagttagtaccactggaaagagacattgttgaaaac
tctgtggctgtgcctcttctaacacatccaggaactgcagcacagaatgagttatctatcaacagcaccactagcaacagcaacgactccccagatggc
agtgagataggagagcaggtacttagcgaggatggttacaaaagagatgggaatggctccgagtcaatacatgtaggagggaaggattttcctactcag
cccatttttagtaaacgaacaggggaacactgctgaagaacacaatgacatagaaacatacggtcatgatggggtacatgcgagaggagagaacagcaca
gcaaatggcatcaggagccaggtaggcatcgttgaaaatgcggaggaagcagagagcagtgtccacggacaggctggtcagaatacaaaatctggaggt
gctagtgatgtaagccagaatggagatgcgacccttgtccaggaaaatgagcctccagaagctagcatcaagaatagcaccaaccatgaggctggaata
cacgggagtggggttgctacacatgaaacgacgcctcagagagaagggctggggagtgagaaccaggggactgaggtgacaccaagcatcggggaagat
gcaggtttggatgatactgatgggagtcctagcgggaacggggtagaggaggatgaagatacaggctctggtgatggtgagggtgcagaagcaggagat
ggaagggagagccatgatggcactaagggccagggggccaatctcatgggggaaacactgaccacagaggtcagagttcagttagtactgaagatgat
gattctaaagaacaagaaggcttcccaaatggacacaatggagacaacagcagtgaggaaaacggtgttgaagaaggcgacagtacccaggcaacgcag
gacaaccaaaagctcagccccaaagacacccgagatgcagagggtgggatcatcagccagtcagaagcatgtccttctgggaagagccaagatcaggga
atagaaactgaaggtcccaacaaaggcaacaaaagtattattaccaaagaatctgggaaactcagtggaagtaaagatagcaatggacaccaaggagtg
gagctggacaaaaggaatagcccaaagcaaggggagtctgacaagcctcaaggcactgctgagaaatcagctgcccacagtaacctgggacacagcagg
ataggtagcagcagcaatagtgatgggcatgacagttacgagttcgatgacgagtccatgcaaggagatgatcccaa*g*agcagcgacgaatctaacgga

agtgacgaaagtgacactaactctgaaagcgccaatgagagtggcagccgtggagatgcttcttacacatctgatgaatcaagtgatgatgacaatgac
agtgactcacatgcgggagaagacgatagcagtgatgactcatctgatacagatgacagtgacagtaatggtgatggtgacagtgacagtaatggtgat
ggtgacagcgagagtgaggacaaggacgaatctgacagcagtgaccatgacaacagcagtgacagtgagagcaaatcagacagcagtgacagtagtgac
gacagcagtgacagcagcgacagtagtgacagcagtgacagcagtgacagtagtgacagtagtgacagcagcgacagcagtgacagcagcgacagcaac
agtagtagtgacagcagcgacagcagcagtagtagtgacagcagcgacagcagtgacagcagtgacagcagtgacagcagcgatagcagtgacagcagt
gacagcagtgacagcagcgatagcagtgacagcagtgacagtagtgacagcagtgacagcagcgacagcagcagtagtagtgacagcagcgacagcagc
agtagtagtgacagcagcgacagcagtgacagcagtgacagcagcgatagcagtgacagcagtgacagcagcagcagcgacagcagcagcagtagcaac
agcagtgacagtagtgacagcagtgacagcagcagcagcagcgacagcagcaacagcagcgacagcagtgacagtagtgacagcagtgacagtagtgac
agcagtgacagcagcaacagtagtgacagcagcgacagcagcagtagtagtgacagcagcgacagcagcagtagtagtgacagcagtgacagtagtgac
agtagtgacagcagtgagagcagtgagagcagcgacagcagcaacagcagtgacagcagcgacagtagtgacagcagtgacagtagcgacagcagcgac
agtagtgacagtagcgacagcagtgacagtagcaacagtagcgacagcagtgacagcagtgacagcagcgacagtagtgacagcagcaacagtagtgac
agcagtgacagtagcgacagtagtgacagcagtgacagcagtgacagcagcgacagtagtgacagcagtgacagtagtgacagcagcgacagtagtgac
agcagtgacagcagtgacagcagtgacagcagcgacagcagcgacagcagtgacagcagcgacagcagcgacagcagtgacagcagcgacagcagcaac
agcagtgacagcagtgacagtgacagcaaggatagcagttctgacagcagtgatggtgacagcaagtctggtaatggcaacagtgacagcaacagtgac
agcaacagtgacagtgacagtgacagtgaaggcagtgacagtaaccactcaaccagtgatgat**tag**

MKMKIIIYICIWATAWAIPVPQLVPLERDIVENSVAVPLLTHPGTAAQNELSINSTTSNSNDSPDGSEIGEQVLSEDGYKRDGNGSESIHVGGKDFPTQ
PILVNEQGNTAEEHNDIETYGHDGVHARGENSTANGIRSQVGIVENAEEAESSVHGQAGQNTKSGGASDVSQNGDATLVQENEPPEASIKNSTNHEAGI
HGSGVATHETTPQREGLGSENQGTEVTPSIGEDAGLDDTDGSPSGNGVEEDEDTGSGDGEGAEAGDGRESHDGTKGQGGQSHGGNTDHRGQSSVSTEDD
DSKEQEGFPNGHNGDNSSEENGVEEGDSTQATQDNQKLSPKDTRDAEGGIISQSEACPSGKSQDQGIETEGPNKGNKSIITKESGKLSGSKDSNGHQGV
ELDKRNSPKQGESDKPQGTAEKSAAHSNLGHSRIGSSSNSDGHDSYEFDDESMQGDDPKSSDESNGSDESDTNSESANESGSRGDASYTSDESSDDDND
SDSHAGEDDSSDDSSDTDDSDSNGDGDSDSNGDGDSESEDKDESDSSDHDNSSDSESKSDSSDSSDDSSDSSDSSDSSDSSDSSDSSDSSDSSDSSDSN
SSSDSSDSSSSSDSSDSSDSSDSSDSSDSSDSSDSSDSSDSSDSSDSSDSSDSSSSSDSSDSSSSSDSSDSSDSSDSSDSSDSSDSSSSDSSSSSN
SSDSSDSSDSSSSSDSSNSSDSSDSSDSSDSSDSSNSSDSSDSSSSSDSSDSSSSSDSSDSSDSSESSESSDSSNSSDSSDSSDSSDSSDSSD
SSDSSDSSDSSNSSDSSDSSDSSDSSDSSNSSDSSDSSDSSDSSDSSDSSDSSDSSDSSDSSDSSDSSDSSDSSDSSDSSDSSDSSDSSDSSDSSN
SSDSSDSDSKDSSSDSSDGDSKSGNGNSDSNSDSNSDSDSDSEGSDSNHSTSDD*

C) *Dspp*$^{-DPP}$ coding region. This untargeted coding sequence was isolated during screening for the *Dspp*$^{-1fs}$. The sole modification is the introduction of a single "**T**" nucleotide The inserted T (capital and bold) shifts translation to the -2 reading frame, and immediately terminates translation, thereby eliminating all but the 9 amino acids (DDPKSSDES) at the N-terminus of DPP. This T also introduces a *MseI* restriction enzyme cleavage site (5'-TTAA-3). The alternating green and yellow highlighting distinguishes *Dspp* exons 2-5.

atgaaaatgaagataattatatatatatgcatttgggcaactgcctgggccattccggttccccagttagtaccactggaaagagacattgttgaaaac
tctgtggctgtgcctcttctaacacatccaggaactgcagcacagaatgagttatctatcaacagcaccactagcaacagcaacgactccccagatggc
agtgagataggagagcaggtacttagcgaggatggttacaaaagagatgggaatggctccgagtcaatacatgtaggagggaaggattttcctactcag
cccattttagtaaacgaacaggggaacactgctgaagaacacaatgacatagaaacatacggtcatgatggggtacatgcgagaggagagaacagcaca
gcaaatggcatcaggagccaggtaggcatcgttgaaaatgcggaggaagcagagagcagtgtccacggacaggctggtcagaatacaaaatctggaggt
gctagtgatgtaagccagaatggagatgcgacccttgtccaggaaaatgagcctccagaagctagcatcaagaatagcaccaaccatgaggctggaata
cacgggagtggggttgctacacatgaaacgacgcctcagagagaagggctggggagtgagaaccaggggactgaggtgacaccaagcatcggggaagat
gcaggtttggatgatactgatgggagtcctagcgggaacggggtagaggaggatgaagatacaggctctggtgatggtgagggtgcagaagcaggagat
ggaagggagagccatgatggcactaagggccagggggccaatctcatggggaaacactgaccacagaggtcagagttcagttagtactgaagatgat
gattctaaagaacaagaaggcttcccaaatggacacaatggagacaacagcagtgaggaaaacggtgttgaagaaggcgacagtacccaggcaacgcag
gacaaccaaaagctcagccccaaagacacccgagatgcagagggtggatcatcagccagtcagaagcatgtccttctgggaagagccaagatcaggga
atagaaactgaaggtcccaacaaaggcaacaaaagtattattaccaaagaatctgggaaactcagtggaagtaaagatagcaatggacaccaaggagtg
gagctggacaaaaggaatagcccaaagcaaggggagtctgacaagcctcaaggcactgctgagaaatcagctgcccacagtaacctgggacacagcagg
ataggtagcagcagcaatagtgatgggcatgacagttacgagttcgatgacgagtccatgcaaggagatgatcccaagagcagcgacgaatct**Taa**cgg
aagtgacgaaagtgacactaactctgaaagcgccaatgagagtggcagccgtggagatgcttcttacacatctgatgaatcaagtgatgatgacaatga
cagtgactcacatgcgggagaagacgatagcagtgatgactcatctgatacagatgacagtgacagtaatggtgatggtgacagtgacagtaatggtga
tggtgacagcgagagtgaggacaaggacgaatctgacagcagtgaccatgacaacagcagtgacagtgagagcaaatcagacagcagtgacagtagtga
cgacagcagtgacagcagcgacagtagtgacagcagtgacagcagtgacagtagtgacagtagtgacagcagcgacagcagtgacagcagcgacagcaa
cagtagtagtgacagcagcgacagcagcagtagtagtgacagcagcgacagcagtgacagcagtgacagcagtgacagcagcgatagcagtgacagcag
tgacagcagtgacagcagcgatagcagtgacagcagtgacagtagtgacagcagtgacagcagcgacagcagcagtagtagtgacagcagcgacagcag
cagtagtagtgacagcagcgacagcagtgacagcagtgacagcagcgatagcagtgacagcagtgacagcagcagcagcgacagcagcagcagtagcaa
cagcagtgacagtagtgacagcagtgacagcagcagcagcagcgacagcagcaacagcagcgacagcagtgacagtagtgacagcagtgacagtagtga
cagcagtgacagcagcaacagtagtgacagcagcgacagcagcagtagtagtgacagcagcgacagcagcagtagtagtgacagcagtgacagtagtga
cagtagtgacagcagtgagagcagtgagagcagcgacagcagcaacagcagtgacagcagcgacagtagtgacagcagtgacagtagcgacagcagcga
cagtagtgacagtagcgacagcagtgacagtagcaacagtagcgacagcagtgacagcagtgacagcagcgacagtagtgacagcagcaacagtagtga
cagcagtgacagtagcgacagtagtgacagcagtgacagcagtgacagcagcgacagtagtgacagcagtgacagtagtgacagcagcgacagtagtga
cagcagtgacagcagtgacagcagtgacagcagcgacagcagcgacagcagtgacagcagcgacagcagcgacagcagtgacagcagcgacagcagcaa
cagcagtgacagcagtgacagtgacagcaaggatagcagttctgacagcagtgatggtgacagcaagtctggtaatggcaacagtgacagcaacagtga
cagcaacagtgacagtgacagtgacagtgaaggcagtgacagtaaccactcaaccagtgatgat**tag**

MKMKIIIYICIWATAWAIPVPQLVPLERDIVENSVAVPLLTHPGTAAQNELSINSTTSNSNDSPDGSEIGEQVLSEDGYKRDGNGSESIHVGGKDFPTQ
PILVNEQGNTAEEHNDIETYGHDGVHARGENSTANGIRSQVGIVENAEEAESSVHGQAGQNTKSGGASDVSQNGDATLVQENEPPEASIKNSTNHEAGI
HGSGVATHETTPQREGLGSENQGTEVTPSIGEDAGLDDTDGSPSGNGVEEDEDTGSGDGEGAEAGDGRESHDGTKGQGGQSHGGNTDHRGQSSVSTEDD
DSKEQEGFPNGHNGDNSSEENGVEEGDSTQATQDNQKLSPKDTRDAEGGIISQSEACPSGKSQDQGIETEGPNKGNKSIITKESGKLSGSKDSNGHQGV
ELDKRNSPKQGESDKPQGTAEKSAAHSNLGHSRIGSSSNSDGHDSYEFDDESMQGDDPKSSDES*

Figure S3. Genotyping *Dspp*$^{+}$, *Dspp*$^{-1fs}$, and *Dspp*$^{-DPP}$ Offspring.

A) Genotyping of chimeras and F1 offspring was conducted with two PCR primer pairs: This primer set was used for genotyping viable chimeras using tail DNA, which identified two mice that harbored the perfectly targeted *Dspp*$^{-1fs}$ allele (lanes 2 and 4, bottom). DNA sequencing of the "wild-type 184 bp" band in lane 2 revealed a single T insertion that generated another important modified mouse: *Dspp*$^{-DPP}$, so a single modified mouse was a compound heterozygote with a *Dspp*$^{-1fs}$ and a *Dspp*$^{-DPP}$ allele. PCR was conducted using the following conditions: 95 °C for 2 min, followed by 30 cycles of 95 °C for 30 sec, 62 °C for 30 sec, and 72 °C for 45 sec, and a final extension at 72 °C for 1 min.

Dspp-WT-F 5'ATGCAAGGAGATGATCCCAA; *Dspp*-WT-R-5'ATGAGTCATCACTGCTATCGTCTT; (rev comp: aagacgatagcagtgatgactcat; 184 bp).

atgcaaggagatgatcccaagagcagcgacgaatctaacggaagtgacgaa
agtgacactaactctgaaagcgccaatgagagtggcagccgtggagatgct
tcttacacatctgatgaatcaagtgatgatgacaatgacagtgactcacat
gcgggagaagacgatagcagtgatgactcat

Dspp-Shift-F 5'TACAAAGACGATGACGACAAGG; *Dspp*-Shift-R 5'CTGCTGTCTGATTTGCTCTCA (rev comp: tgagagcaaatcagacagcag; 337 bp product). A dash marks the location of the "G" deleted to cause the -1 frameshift.

tacaaagacgatgacgacaaggatgatcccaa-agc**C**gc**C**ac**C**aa**C**ct**G**ac**C**
ga**G**gt**C**acgaaagtgacactaactctgaaagcgccaatgagagtggcagccg
tggagatgcttcttacacatctgatgaatcaagtgatgatgacaatgacagt
gactcacatgcgggagaagacgatagcagtgatgactcatctgatacagatg
acagtgacagtaatggtgatggtgacagtgacagtaatggtgatggtgacag
cgagagtgaggacaaggacgaatctgacagcagtgaccatgacaacagcagt
gacagtgagagcaaatcagacagcag

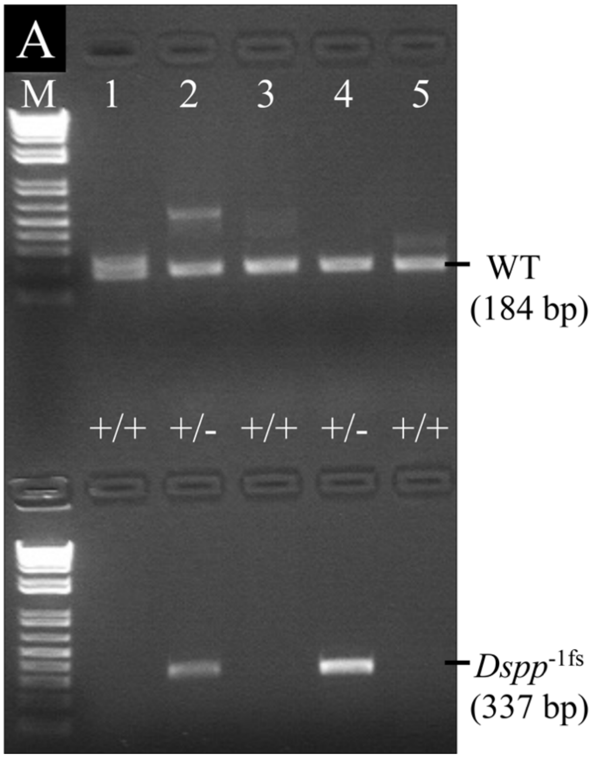

B) Genotyping optimized for screening offspring:

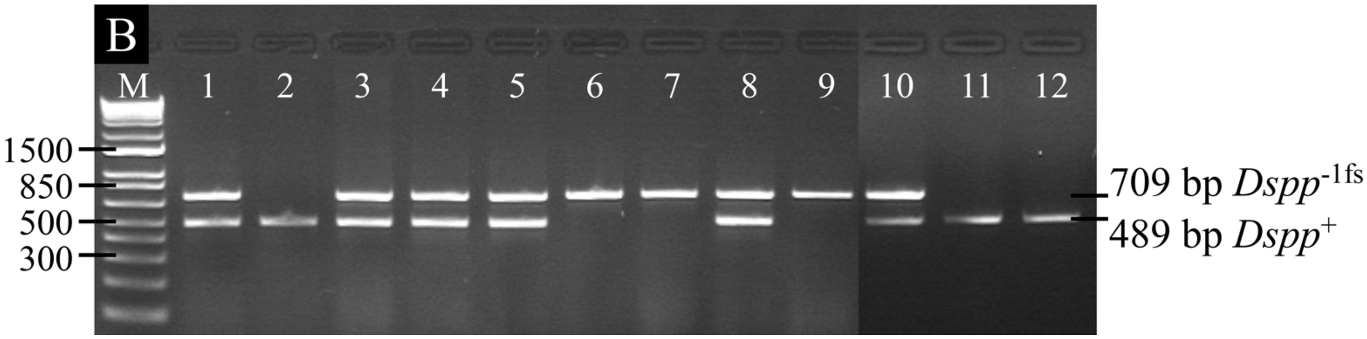

Dspp-Shift-F-DSPP-WT-New-F 5'CCAAACCCTGATACAGAGCA; DSPP-WT-New-R 5'ATGAGTCATCACTGC TATCGTCTT; (rev comp: 5'aagacgatagcagtgatgactcat; 489 bp product). The unhighlighted sequence is from intron 4; highlighted sequence is from exon 5.

CCAAACCCTGATACAGAGCACATTTGCATTCAAGTACTAGATCAGCAGGCGTGCATGAATCACTGCACTGACAGCCTATACTCCTGTTCCTAAGGTCAC
TTCCTGAGACAGTTCTCCTCAGACCATGATGTTTTGTAGCAAATATTCACTAATTATCCATTCTTCTTTATATCGTTCCACAGGGAATAGAAACTGAAG
GTCCCAACAAAGGCAACAAAAGTATTATTACCAAAGAATCTGGGAAACTCAGTGGAAGTAAAGATAGCAATGGACACCAAGGAGTGGAGCTGGACAAAA
GGAATAGCCCAAAGCAAGGGGAGTCTGACAAGCCTCAAGGCACTGCTGAGAAATCAGCTGCCCACAGTAACCTGGGACACAGCAGGATAGGTAGCAGCA
GCAATAGTGATGGGCATGACAGTTACGAGTTCGATGACGAGTCCATGCAAGGAGATGATCCCAAGAGCAGCGACGAATCTAACGGAAGTGACG

Dspp-MU-F-709 5’CCTGAGCCACCTCTCCAATA and DSPP1-MU-R-709 5’CCTTGTCGTCATCGTCTTTGTA (rev comp: 5'tacaaagacgatgacgacaagg; 709 bp product). The unhighlighted sequence is from intron 4; highlighted sequence is from exon 5. Introduced coding sequence for the Flag epitope is in bold.

cctgagccacctctccaatatgtctctgatataggacaatttttaaaaattcacaaacttctgtaaaattagtcagaatgctagaagtcaagctgcataacggtt
ccatgatgtctttgtaagacattttattagtttacattcatcacacagaatgaccagcttcactatgacactttcattattatgcttcaagcccttatgagttag
aaacctggatggcttattagaggatccaaaccctgatacagagcacatttgcattcaagtactagatcagcaggcgtgcatgaatcactgcactgacagcctata
ctcctgttcctaaggtcacttcctgagacagttctcctcagaccatgatgttttgtagcaaatattcactaattatccattcttctttatatcgttccacaggga
atagaaactgaaggtcccaacaaaggcaacaaaagtattattaccaaagaatctgggaaactcagtggaagtaaagatagcaatggacaccaaggagtggagctg
gacaaaaggaatagcccaaagcaaggggagtctgacaagcctcaaggcactgctgagaaatcagctgcccacagtaacctgggacacagcaggataggtagcagc
agcaatagtgatgggcatgacagttacgagttcgatgacgagtccatgcaagga**GACTACAAAGACGATGACGACAAG**g

C) Genotyping of *Dspp*$^{-DPP}$

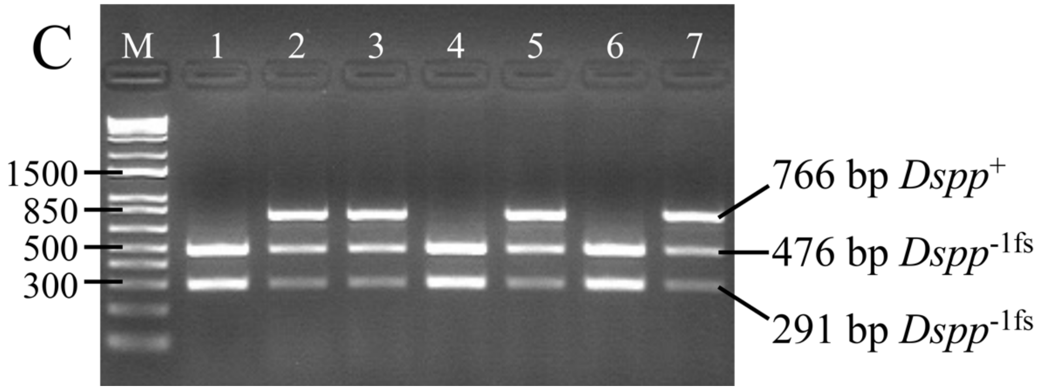

*Dspp*1-2 WT-New-F 5'CCAAACCCTGATACAGAGCA and *Dspp*1-shift-R 5'CTGCTGTCTGATTTGCTCTCA-3' (rev comp: 5'tgagagcaaatcagacagcag; 766 bp product in *Dspp*$^{-1fs}$ and 767 bp in *Dspp*$^{-DPP}$). PCR was conducted using the following conditions: 95 °C for 2 min, followed by 30 cycles of 95 °C for 30 sec, 62 °C for 30 sec, and 72 °C for 45 sec, and a final extension at 72 °C for 1 min. There are no *MseI* (5'-TTAA-3) restriction sites in the amplification product for the *Dspp*$^{+}$ allele and only 1 *MseI* restriction site in the *Dspp*$^{-DPP}$ PCR product where *MseI* (5'-TTAA-3) cleaves after the inserted **T**, generating a 476 and a 291 bp cleavage product.

767 bp *Dspp*$^{-DPP}$ PCR amplification product (The unhighlighted sequence is from intron 4; highlighted sequence is from exon 5.)

ccaaaccctgatacagagcacatttgcattcaagtactagatcagcaggcgtgcatgaatcactgcactgacagcctatactcctgttcctaaggtcac
ttcctgagacagttctcctcagaccatgatgttttgtagcaaatattcactaattatccattcttctttatatcgttccacagggaatagaaactgaag
gtcccaacaaaggcaacaaaagtattattaccaaagaatctgggaaactcagtggaagtaaagatagcaatggacaccaaggagtggagctggacaaaa
ggaatagcccaaagcaaggggagtctgacaagcctcaaggcactgctgagaaatcagctgcccacagtaacctgggacacagcaggataggtagcagca
gcaatagtgatgggcatgacagttacgagttcgatgacgagtccatgcaaggagatgatcccaagagcagcgacgaatc***T****taac*ggaagtgacgaaagt
gacactaactctgaaagcgccaatgagagtggcagccgtggagatgcttcttacacatctgatgaatcaagtgatgatgacaatgacagtgactcacat
gcgggagaagacgatagcagtgatgactcatctgatacagatgacagtgacagtaatggtgatggtgacagtgacagtaatggtgatggtgacagcgag
agtgaggacaaggacgaatctgacagcagtgaccatgacaacagcagtgacagtgagagcaaatcagacagcag

476 bp *MseI* cleavage product (The unhighlighted sequence is from intron 4; highlighted sequence is from exon 5.)

ccaaaccctgatacagagcacatttgcattcaagtactagatcagcaggcgtgcatgaatcactgcactgacagcctatactcctgttcctaaggtcac
ttcctgagacagttctcctcagaccatgatgttttgtagcaaatattcactaattatccattcttctttatatcgttccacagggaatagaaactgaag
gtcccaacaaaggcaacaaaagtattattaccaaagaatctgggaaactcagtggaagtaaagatagcaatggacaccaaggagtggagctggacaaaa
ggaatagcccaaagcaaggggagtctgacaagcctcaaggcactgctgagaaatcagctgcccacagtaacctgggacacagcaggataggtagcagca
gcaatagtgatgggcatgacagttacgagttcgatgacgagtccatgcaaggagatgatcccaagagcagcgacgaatcT

291 bp *MseI* cleavage product (The highlighted sequence is from exon 5.)

taacggaagtgacgaaagtgacactaactctgaaagcgccaatgagagtggcagccgtggagatgcttcttacacatctgatgaatcaagtgatgatga
caatgacagtgactcacatgcgggagaagacgatagcagtgatgactcatctgatacagatgacagtgacagtaatggtgatggtgacagtgacagtaa
tggtgatggtgacagcgagagtgaggacaaggacgaatctgacagcagtgaccatgacaacagcagtgacagtgagagcaaatcagacagcag

D) Original gels used in Figure S3A-S3C. Genotyping *Dspp*$^{+}$, *Dspp*$^{-1fs}$, and *Dspp*$^{-DPP}$ Offspring.

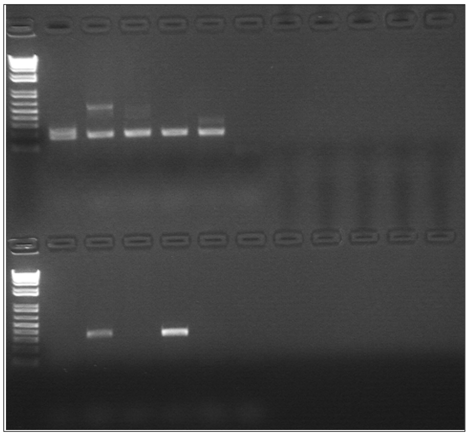

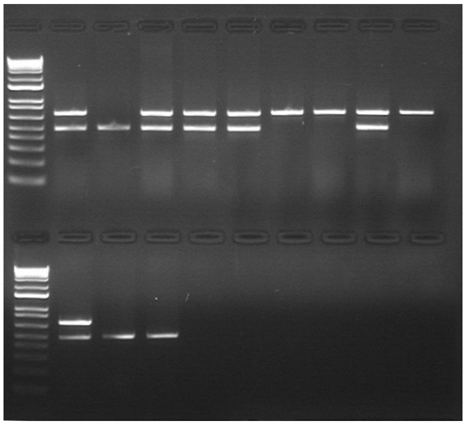

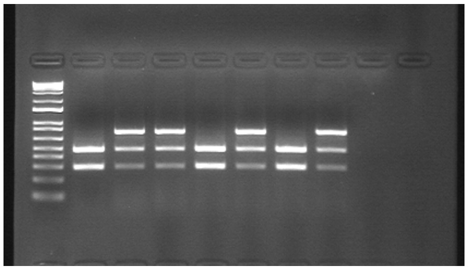

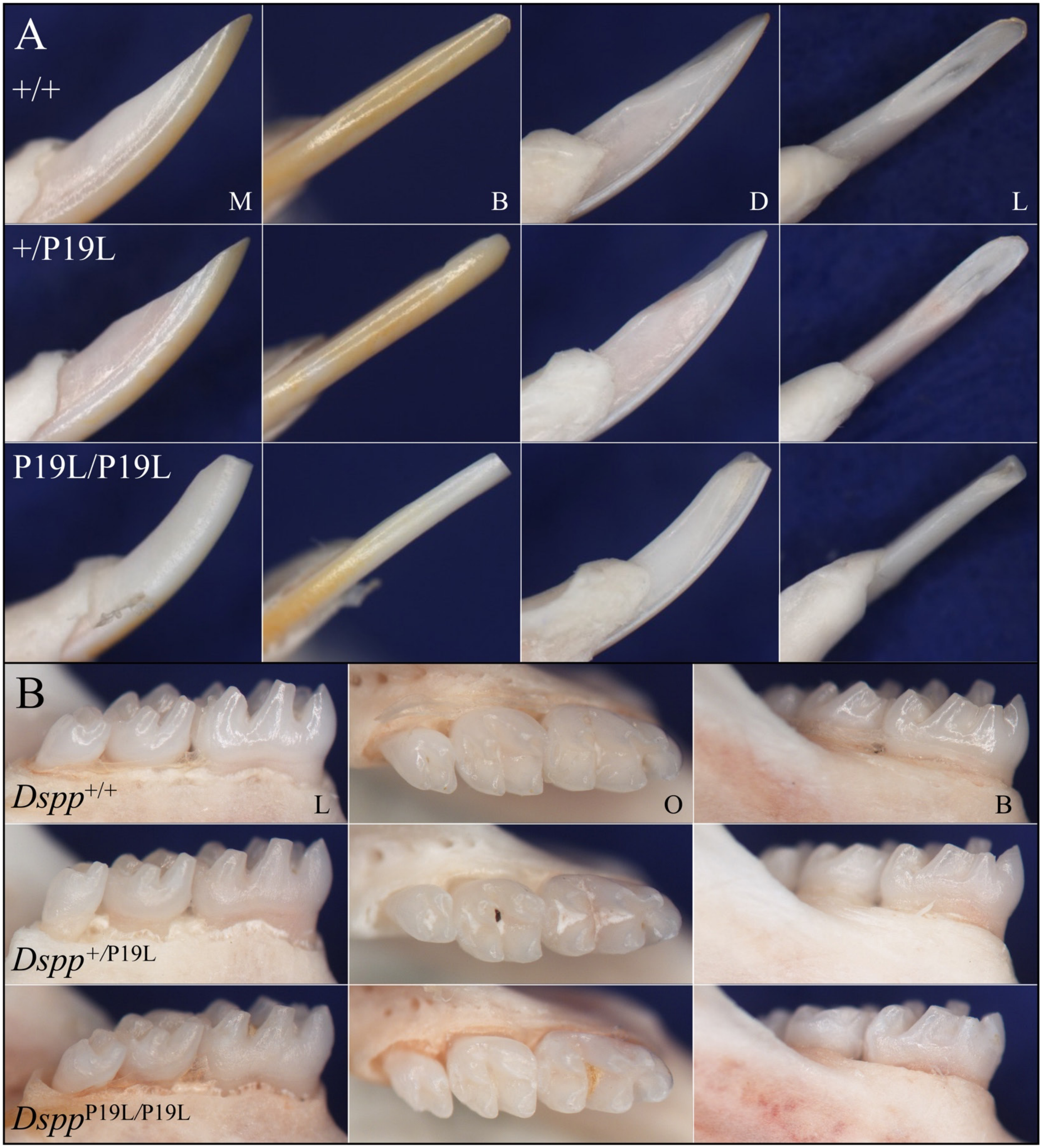

Figure S4. Dissection Microscopy Photographs Comparing 7-week *Dspp*$^{+/+}$, *Dspp*$^{+/P19L}$, and *Dspp*$^{P19L/P19L}$ Mice. ***A:*** Close up photographs of the erupted mandibular incisor tip. M, mesial; B, labial; D, distal; L, lingual. Hyper eruption of the *Dspp*$^{+/-1fs}$, and *Dspp*$^{-1fs/-1fs}$ incisors or diminished alveolar bone causes their longer apparent length outside the bone. ***B:*** Lingual (L), Occlusal (O), and buccal (B) views of the mandibular molars. Overall enamel crown morphology is normal in the *Dspp*$^{+/P19L}$ and *Dspp*$^{P19L/P19L}$ mouse incisors, but they are more translucent and lack the surface luster of the *Dspp*$^{+/+}$. The root dentin is not translucent, as was observed in the *Dspp*$^{+/-1fs}$, and *Dspp*$^{-1fs/-1fs}$ molars (see Figure 1).

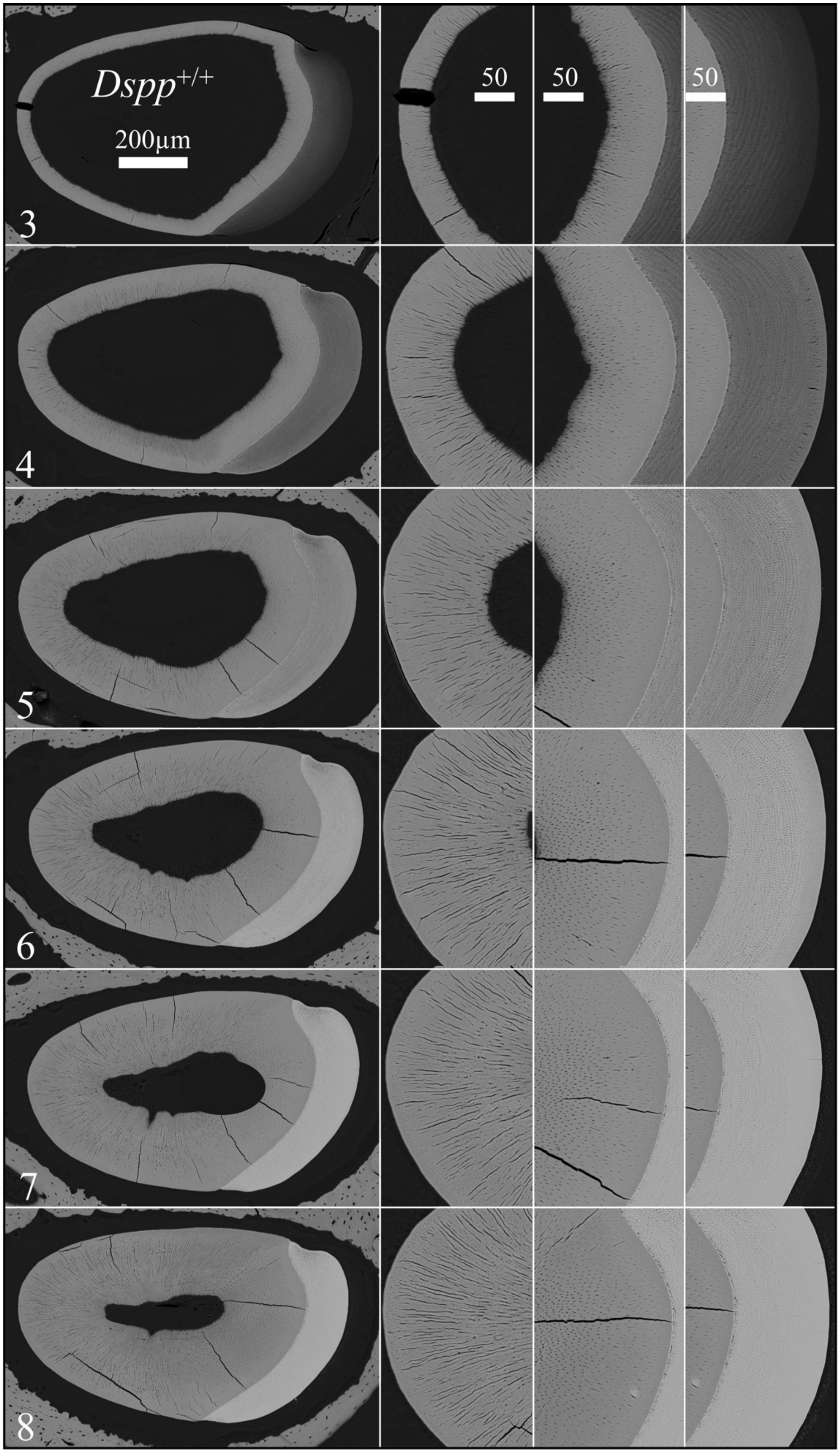

Figure S5. bSEM of a 7-Week *Dspp*$^{+/+}$ Mouse Mandibular Incisor Cross-Sectioned at 1 mm Increments.

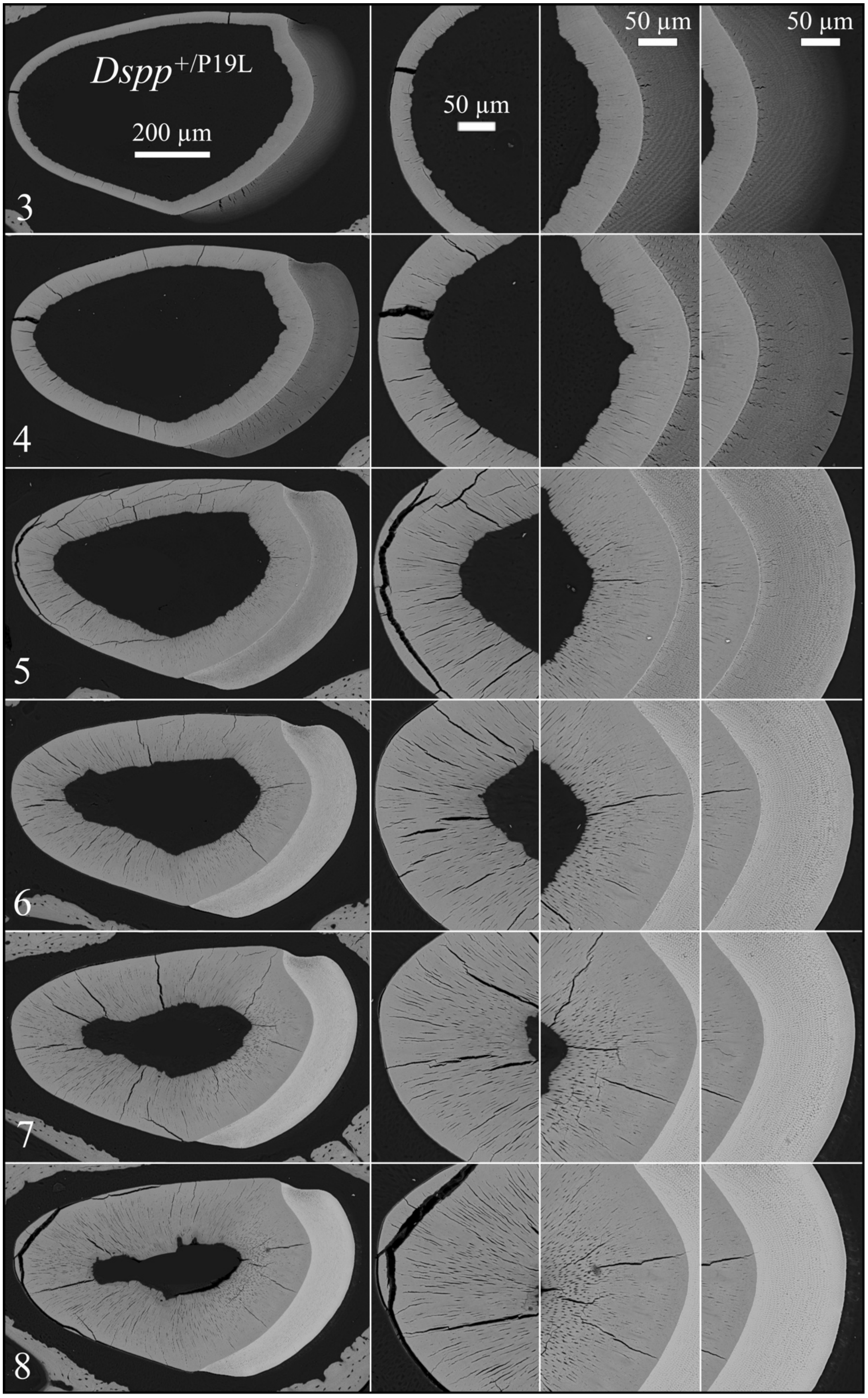

Figure S6a. bSEM of a 7-Week *Dspp*+/P19L Mouse Mandibular Incisor Cross-Sectioned at 1 mm Increments. Note the presence of dentinal tubules and the enlarged pulp chamber (relative to the wild-type), suggesting slower dentin deposition. The enamel layer appears to be normal in thickness and contour but is grayer and the rod outline are prominent relative to the wild-type, suggesting hypomineralization of the enamel.

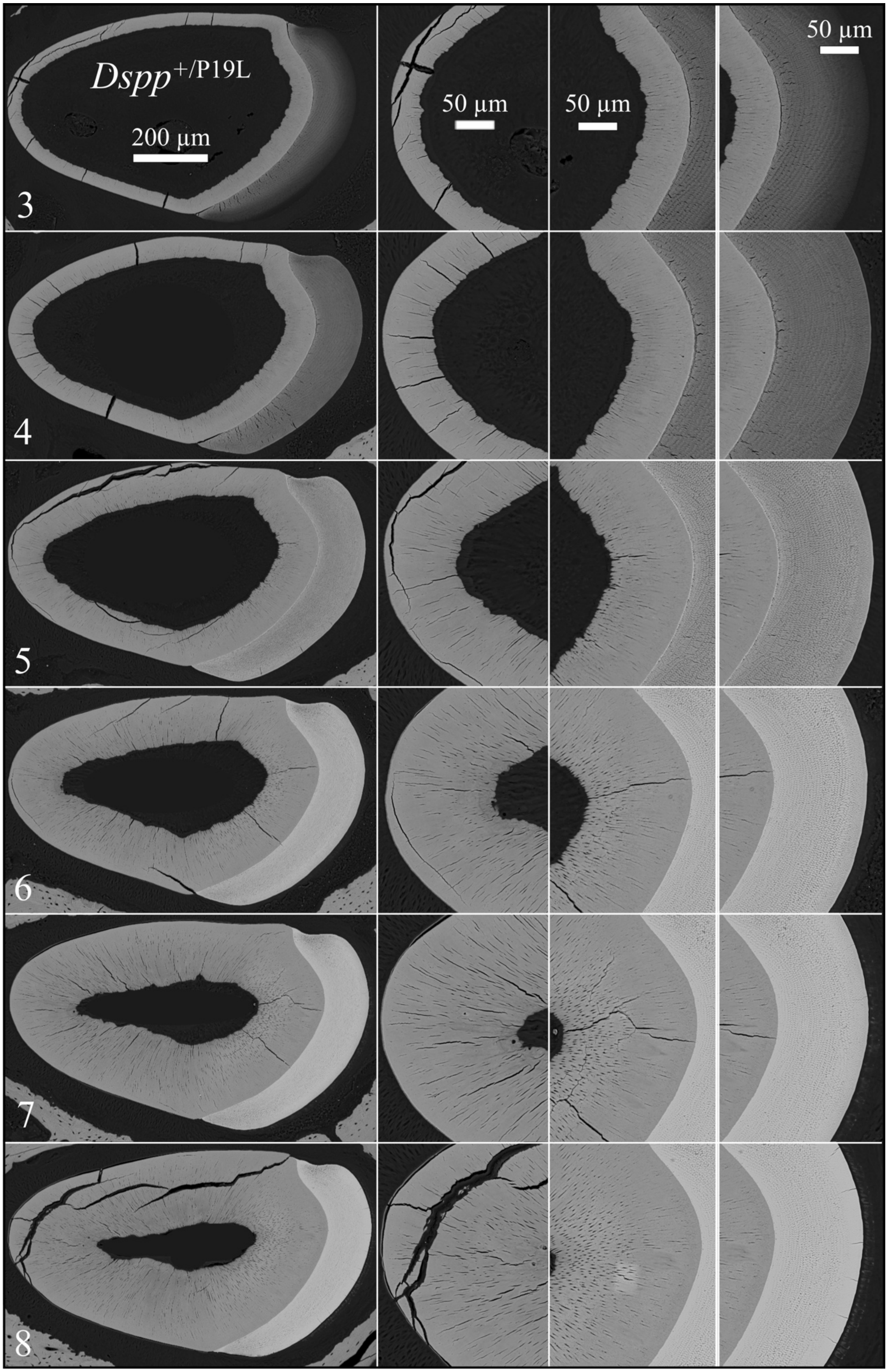

Figure S6b. bSEM of a 7-Week *Dspp*$^{+/P19L}$ Mouse Mandibular Incisor Cross-Sectioned at 1 mm Increments. Note the presence of dentinal tubules and the enlarged pulp chamber (relative to the wild-type), suggesting slower dentin deposition. The enamel layer appears to be normal in thickness and contour but is grayer and the rod outline are prominent relative to the wild-type, suggesting hypomineralization of the enamel.

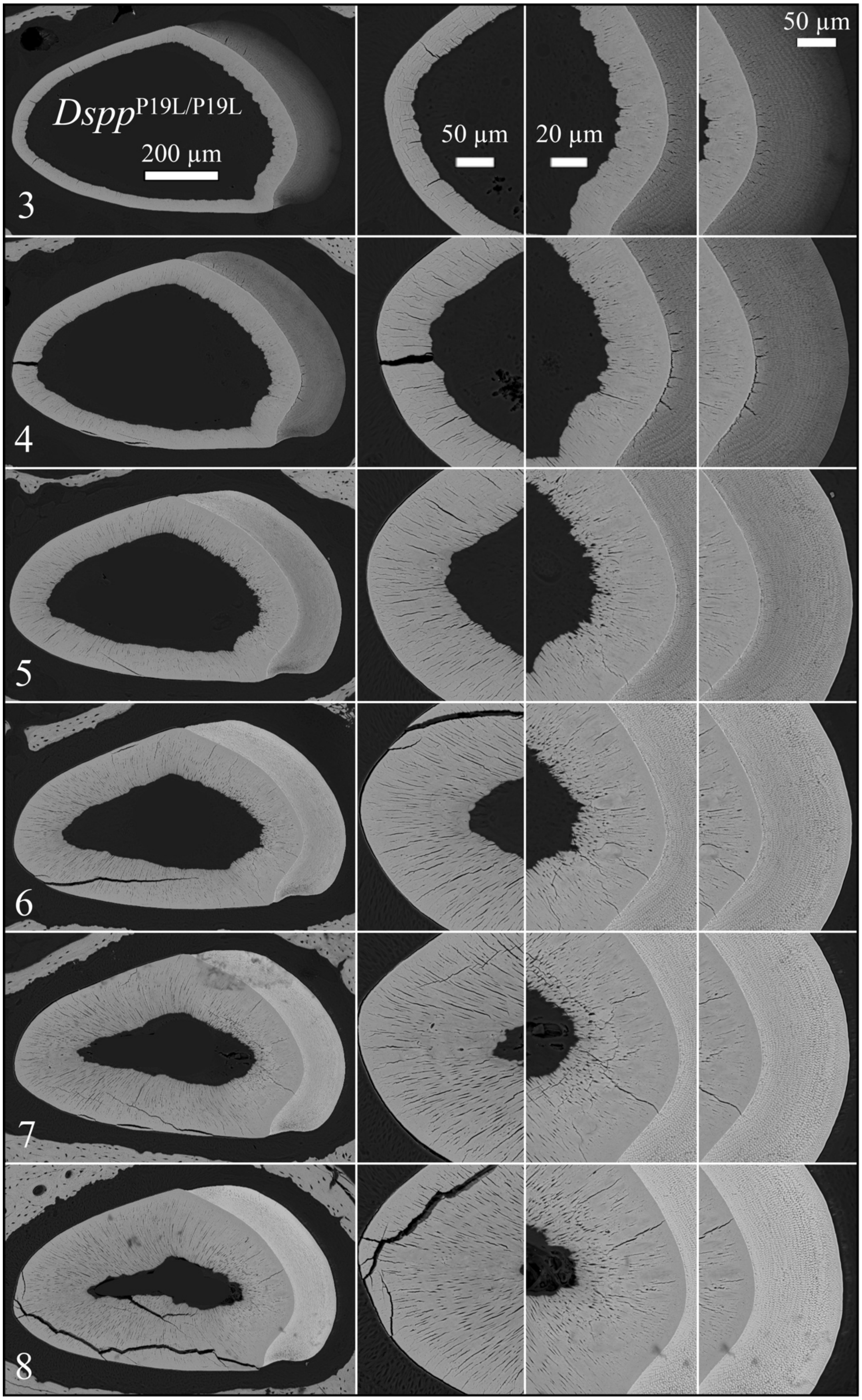

Figure S7a. bSEM of a 7-Week *Dspp*$^{P19L/P19L}$ Mouse Mandibular Incisor Cross-Sectioned at 1 mm Increments. Note the presence of dentinal tubules and the enlarged pulp chamber (relative to the wild-type), suggesting slower dentin deposition. The enamel layer appears to be normal in thickness and contour but is grayer and the rod outline are prominent relative to the wild-type, suggesting hypomineralization of the enamel.

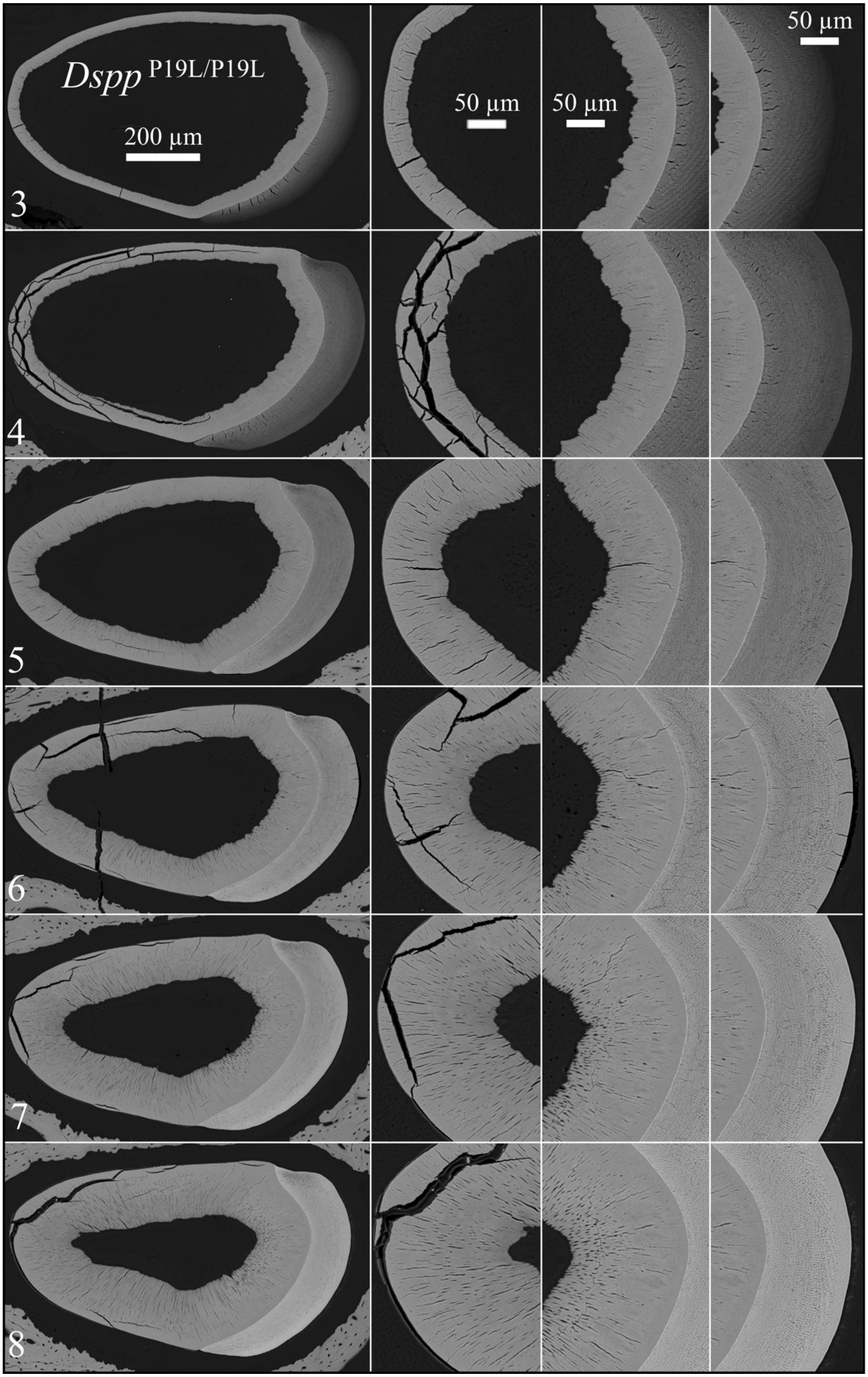

Figure S7b. bSEM of a 7-Week *Dspp*$^{P19L/P19L}$ Mouse Mandibular Incisor Cross-Sectioned at 1 mm Increments. Note the presence of dentinal tubules and the enlarged pulp chamber (relative to the wild-type), suggesting slower dentin deposition. The enamel layer appears to be normal in thickness and contour but is grayer and the rod outline are prominent relative to the wild-type, suggesting hypomineralization of the enamel.

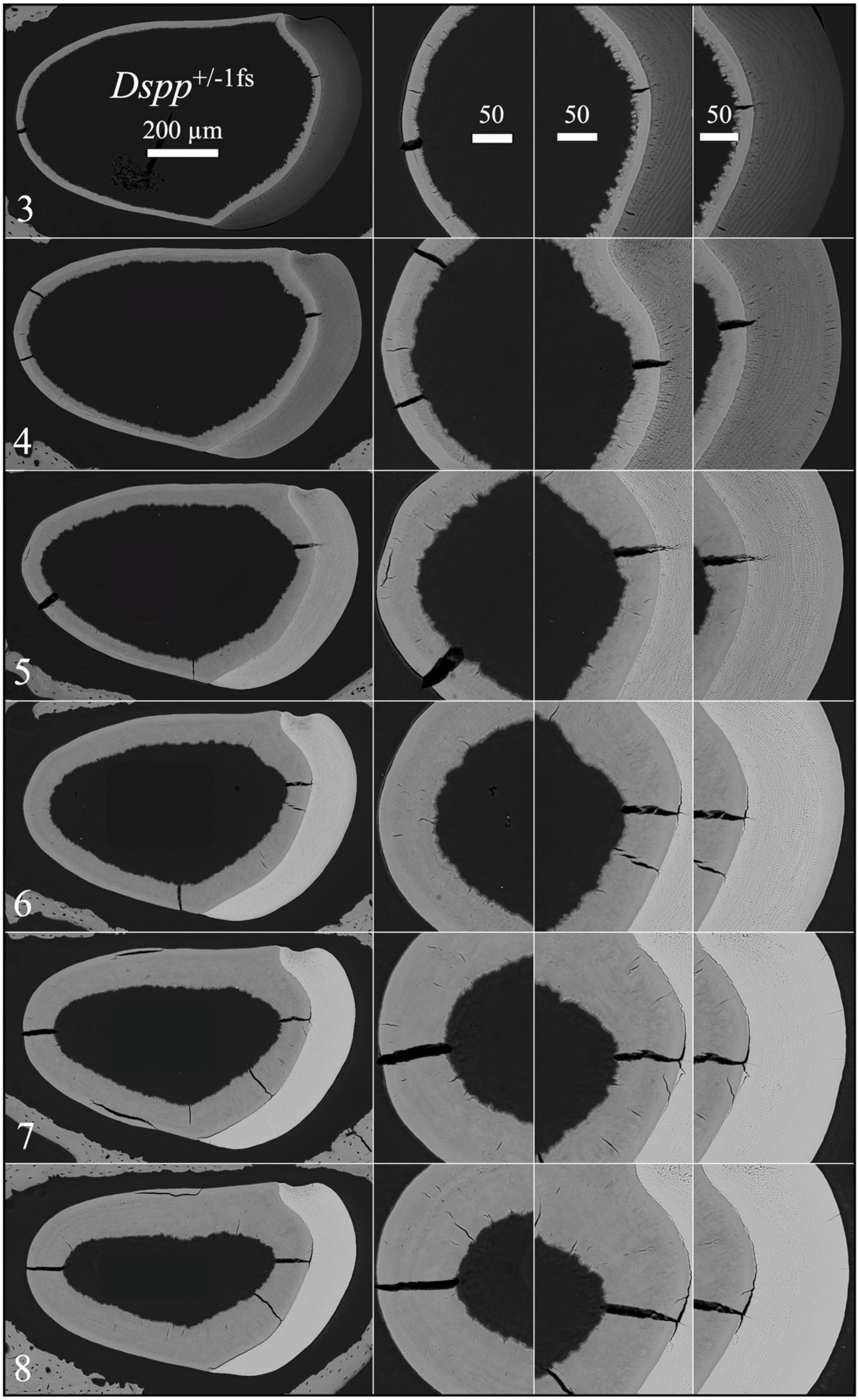

Figure S8a. bSEM of a 7-Week *Dspp*$^{+/-1fs}$ Mouse Mandibular Incisor Cross-Sectioned at 1 mm Increments. Note the absence of dentinal tubules and the enlarged pulp chamber, suggesting very slow dentin deposition by cells that have replaced the original odontoblasts. The enamel appears to be normal in contour, thickness and gray scale (degree of mineralization).

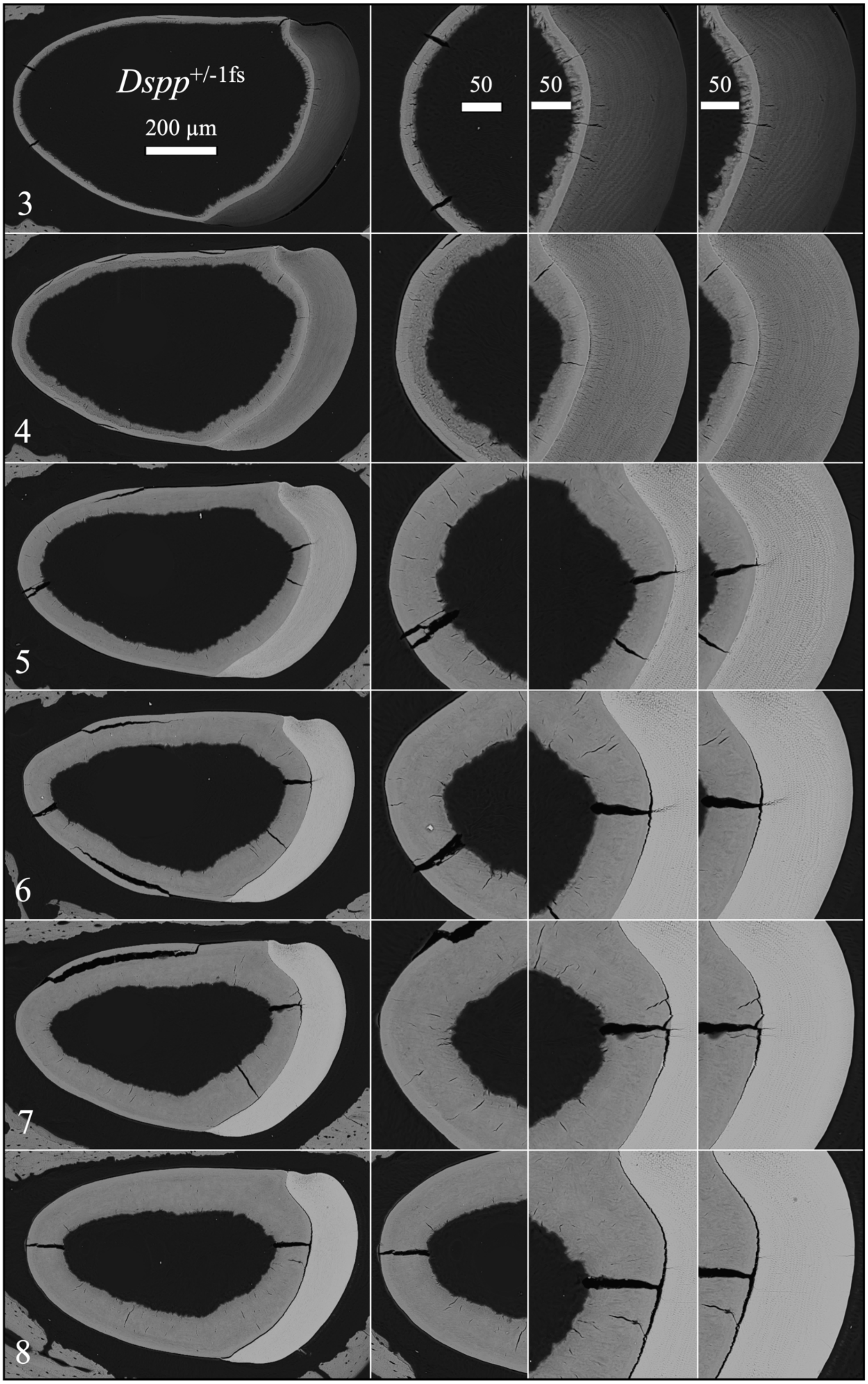

Figure S8b. bSEM of a 7-Week *Dspp*$^{+/-1fs}$ Mouse (568) Mandibular Incisor Cross-Sectioned at 1 mm Increments. Note the absence of dentinal tubules and the enlarged pulp chamber, suggesting very slow dentin deposition by cells that have replaced the original odontoblasts. The enamel appears to be normal in contour, thickness and gray scale (degree of mineralization).

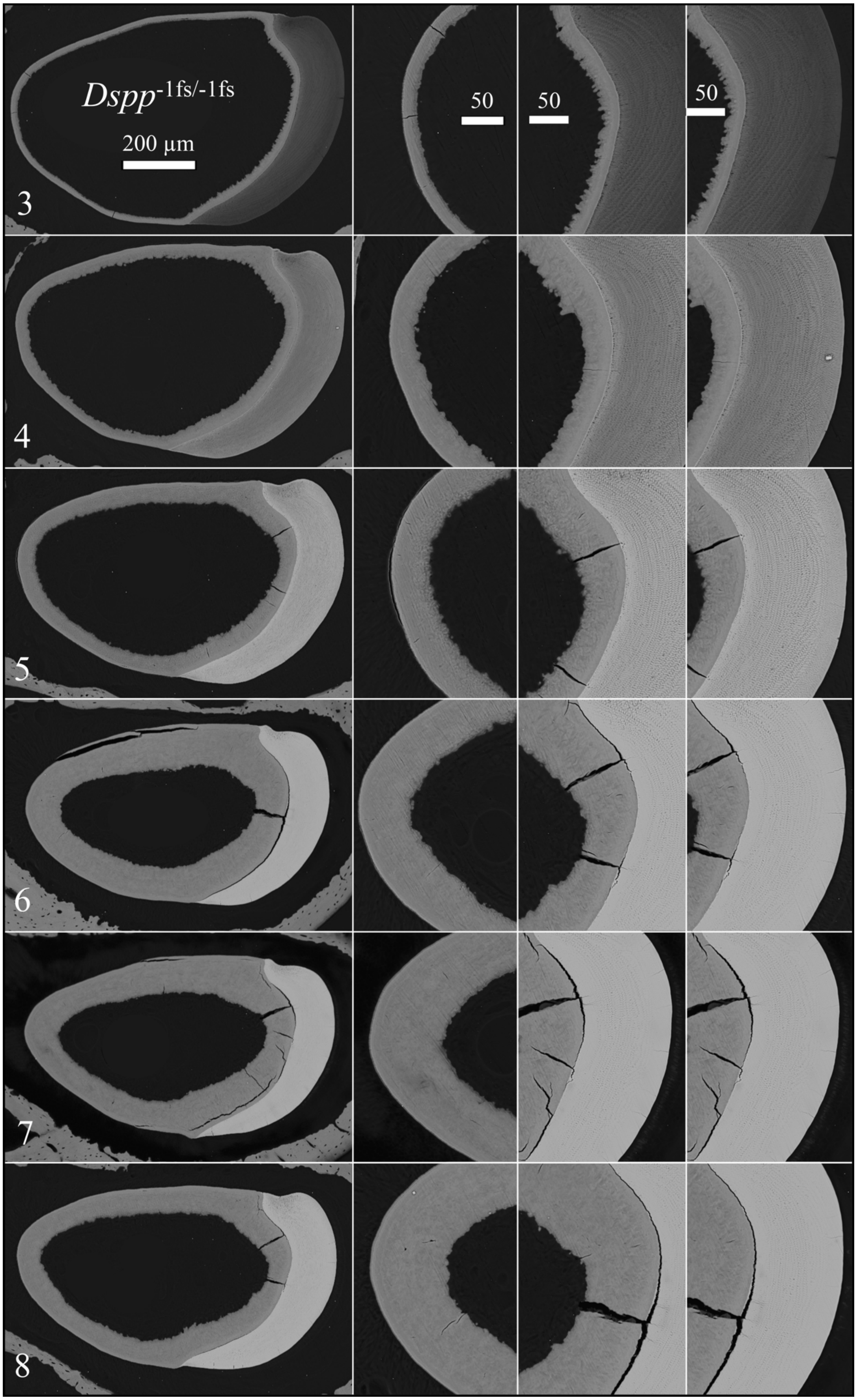

Figure S9a. bSEM of a 7-Week *Dspp*$^{-1fs/-1fs}$ Mouse (570) Mandibular Incisor Cross-Sectioned at 1 mm Increments. Note the absence of dentinal tubules and the enlarged pulp chamber, suggesting very slow dentin deposition by cells that have replaced the original odontoblasts. The enamel appears to be normal in contour, thickness and gray scale (degree of mineralization).

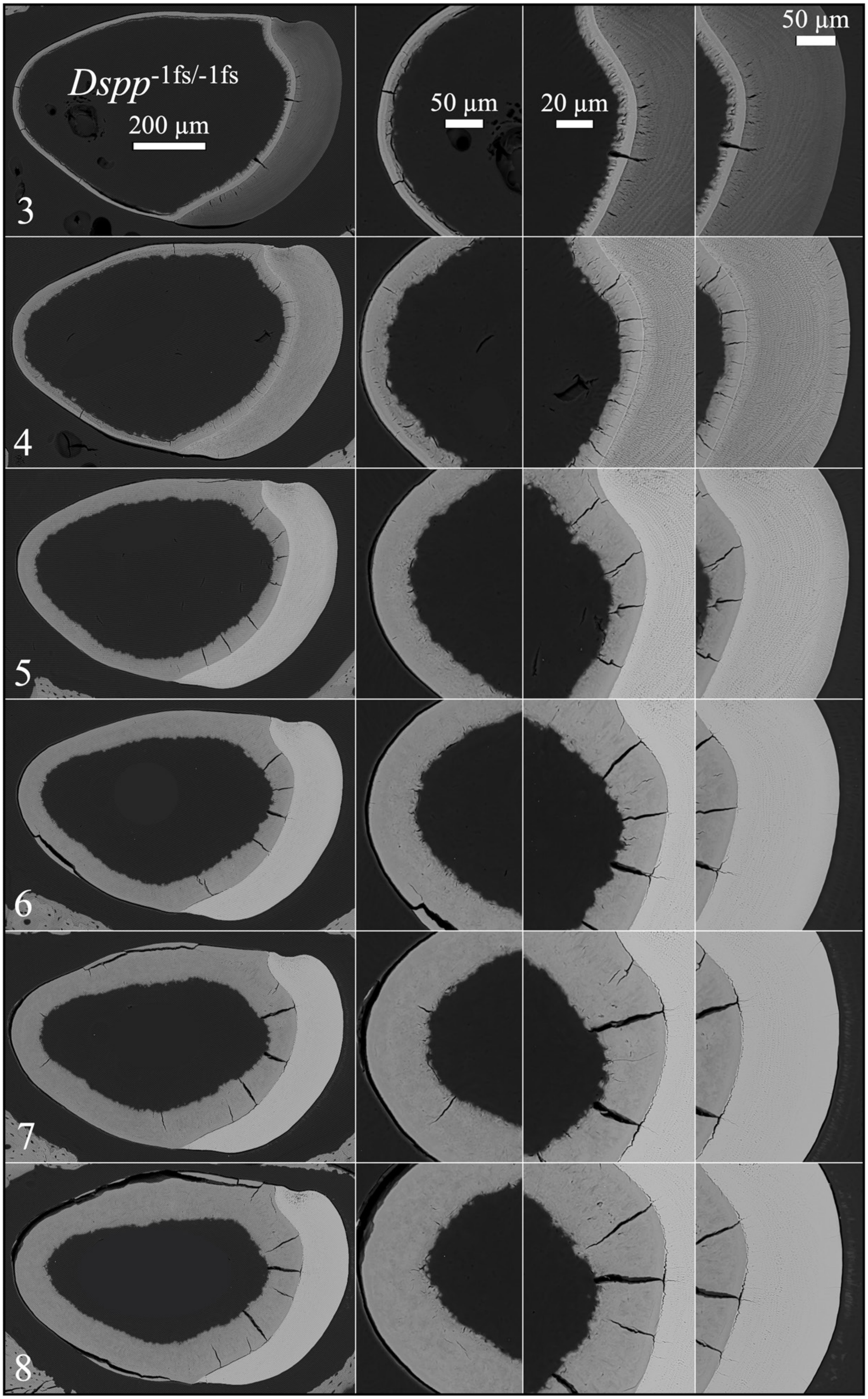

Figure S9b. bSEM of a 7-Week *Dspp*$^{-1fs/-1fs}$ Mouse (571) Mandibular Incisor Cross-Sectioned at 1 mm Increments. Note the absence of dentinal tubules and the enlarged pulp chamber, suggesting very slow dentin deposition by cells that have replaced the original odontoblasts. The enamel appears to be normal in contour, thickness and gray scale (degree of mineralization).

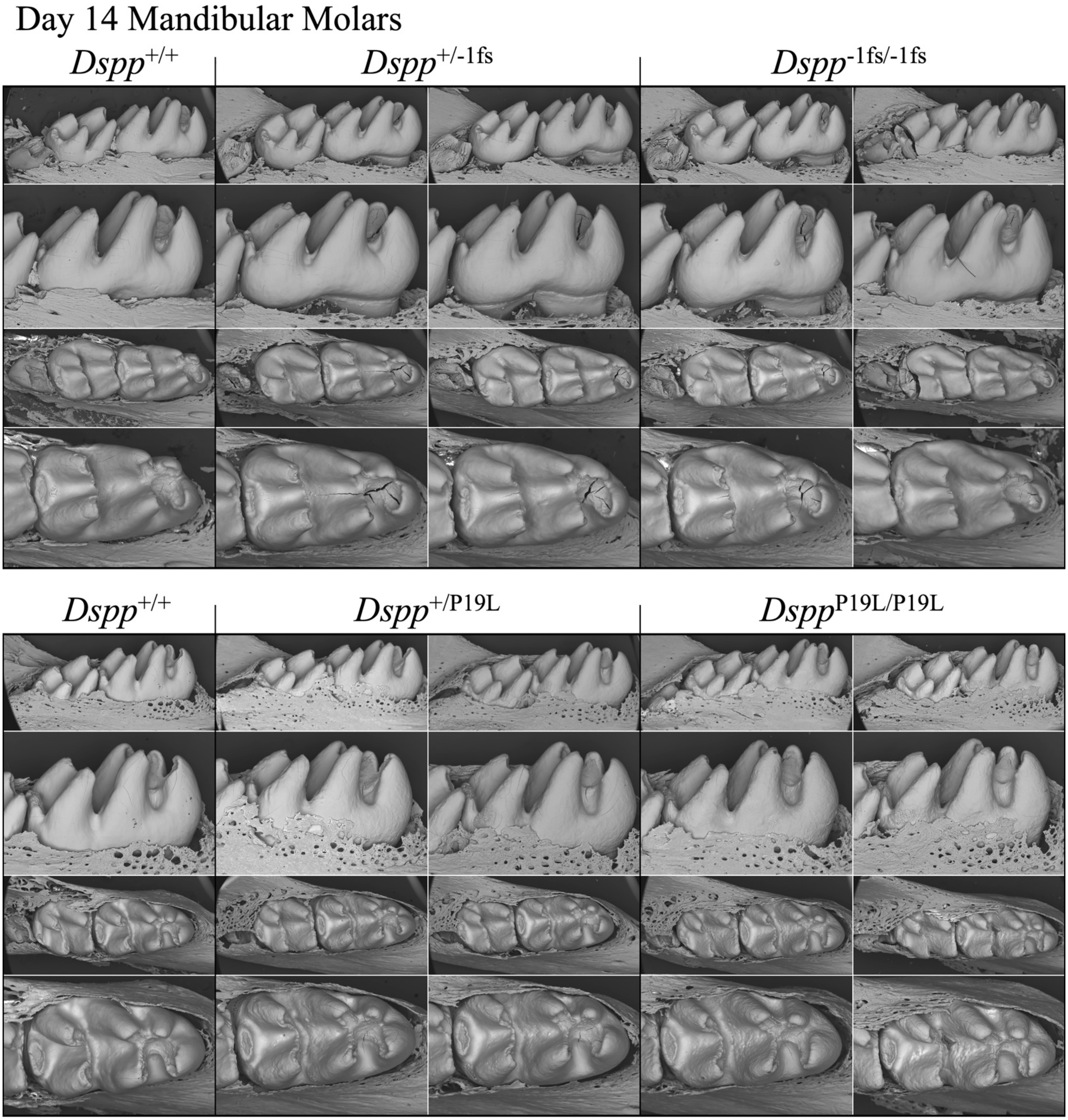

Figure S10. bSEM of Day 14 *Dspp*$^{+/+}$, *Dspp*$^{+/-1fs}$, *Dspp*$^{-1fs/-1fs}$, *Dspp*$^{+/P19L}$, and *Dspp*$^{P19L/P19L}$ Mouse Mandibular Molars. The surface texture of the enamel is coarse in the *Dspp*$^{+/P19L}$, and *Dspp*$^{P19L/P19L}$ mice.

D14 Molars *Dspp*$^{+/+}$

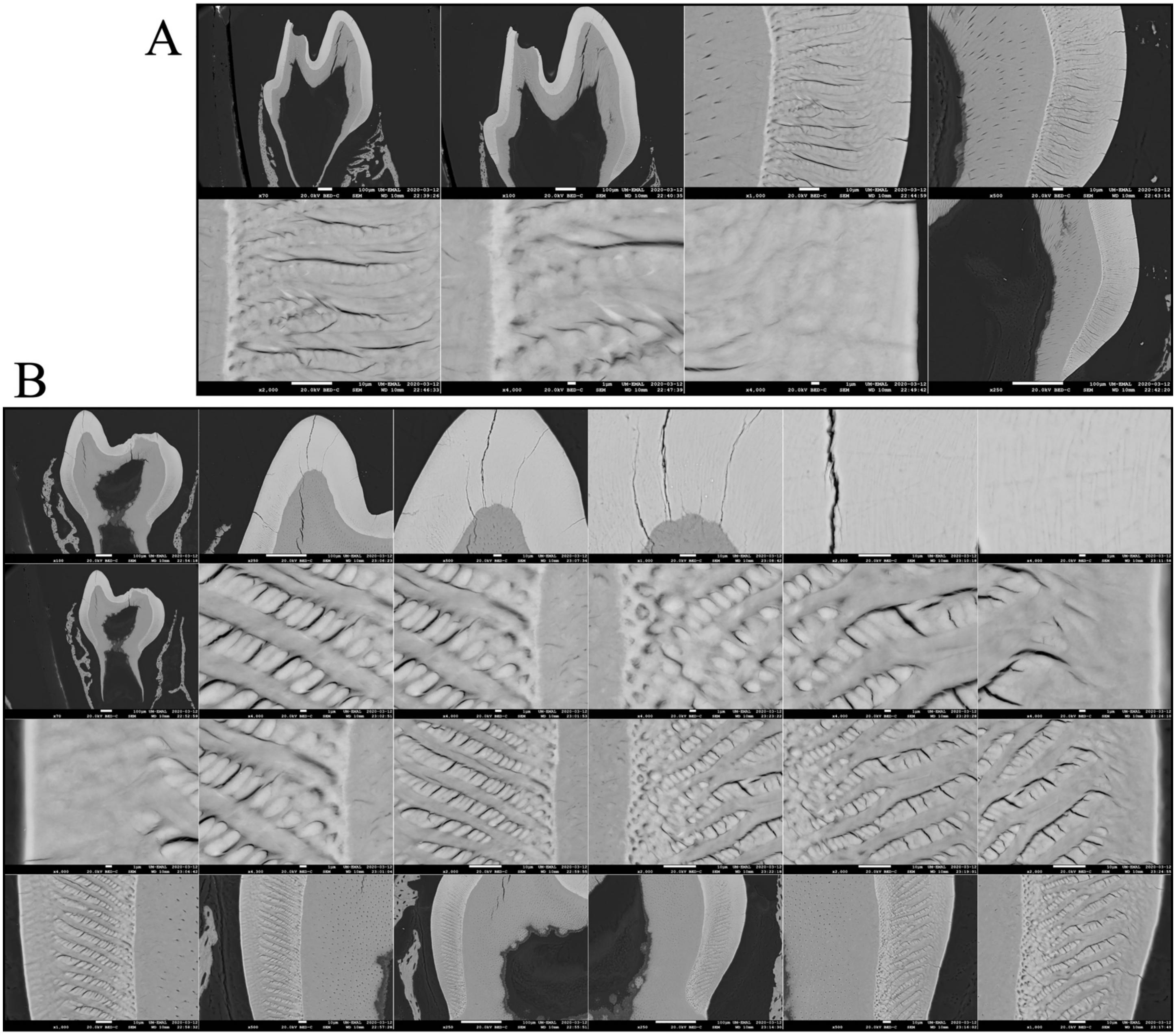

Figure S11. bSEM of D14 *Dspp*$^{+/+}$ Mouse Mandibular Molars. Different D14 *Dspp*$^{+/+}$ molars are shown in A and B.

D14 Molars *Dspp*$^{+/P19L}$

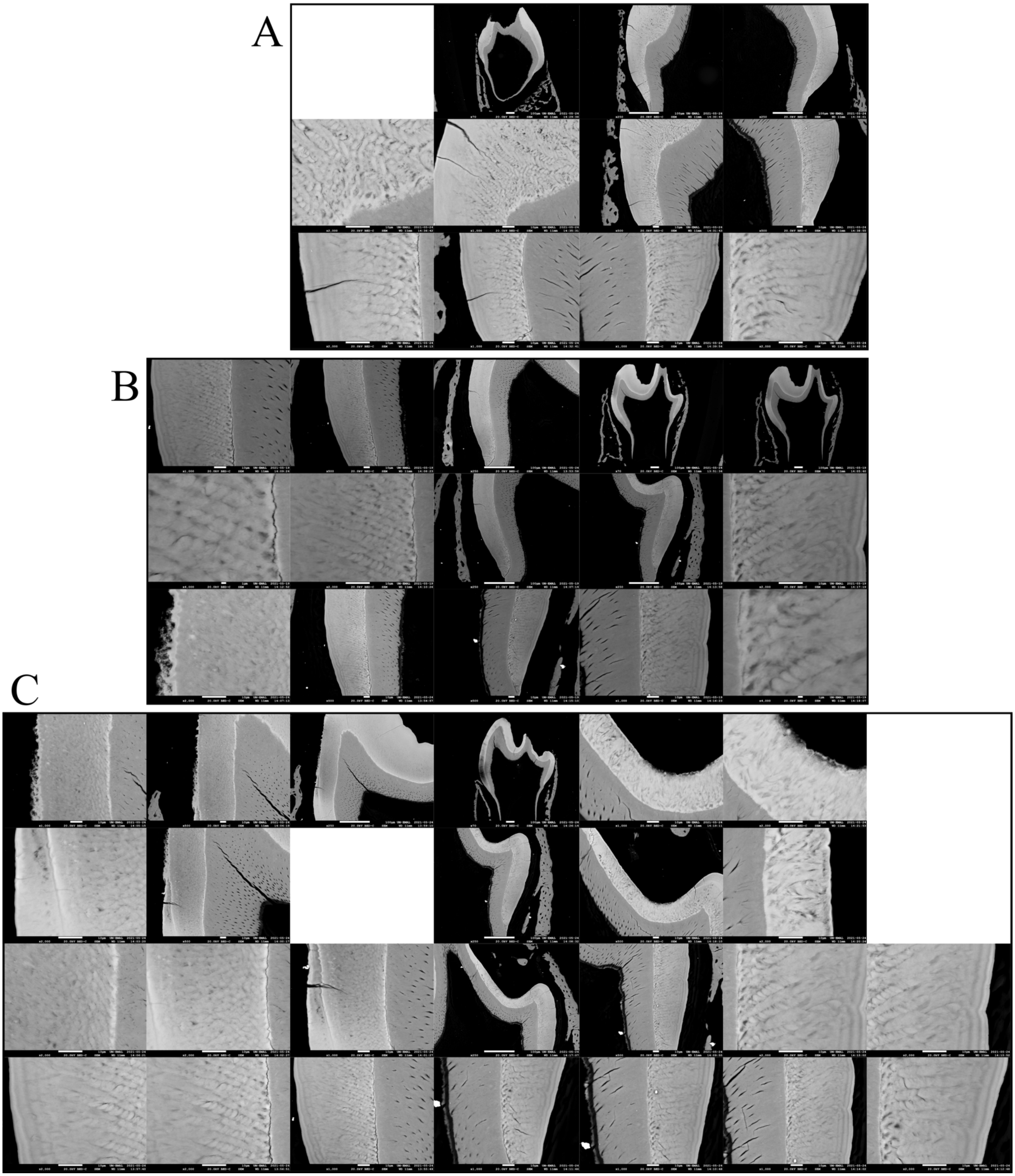

Figure S12. bSEM of D14 *Dspp*$^{+/P19L}$ Mouse Mandibular Molars. Three different D14 *Dspp*$^{+/P19L}$ molars are shown. Note the well-defined dentinal tubules and the lack of enamel rod definition, particularly near the enamel surface.

D14 Molars *Dspp*$^{P19L/P19L}$

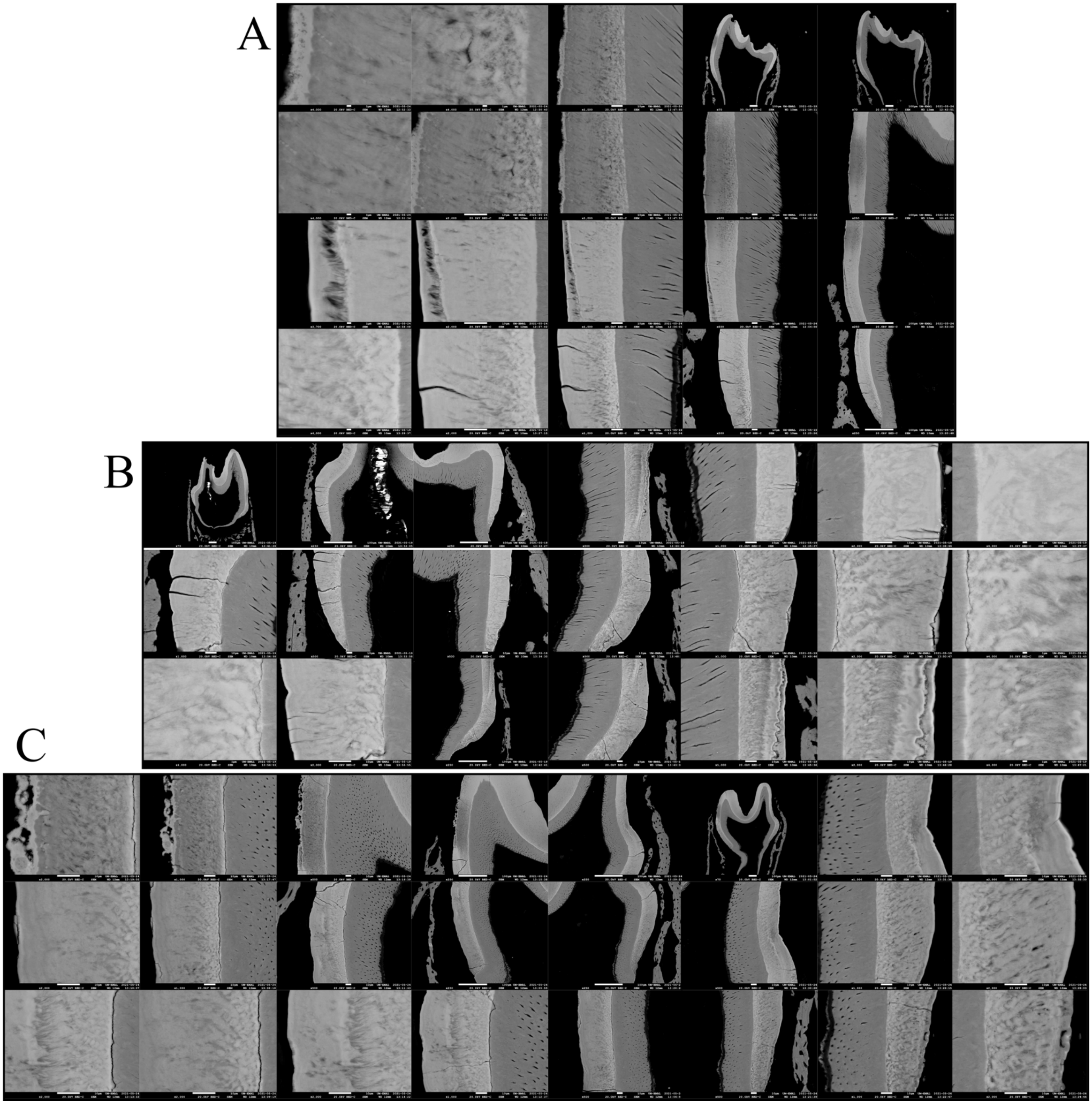

Figure S13. bSEM of D14 *Dspp*$^{P19L/P19L}$ Mouse Mandibular Molars. Note the well-defined dentinal tubules and the lack of enamel rod definition, particularly near the enamel surface.

Dspp$^{+/-1fs}$ D14 Molars

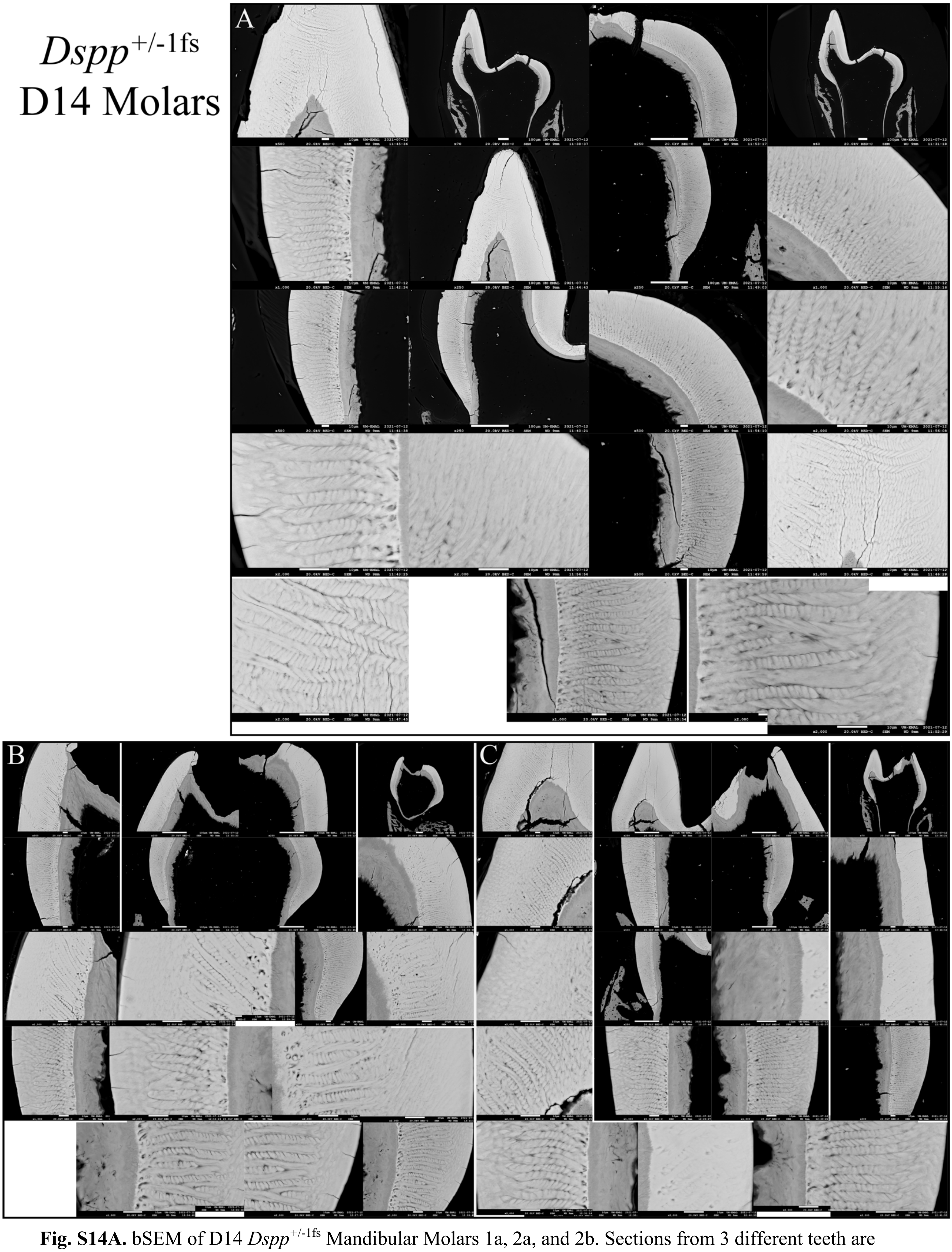

Fig. S14A. bSEM of D14 *Dspp*$^{+/-1fs}$ Mandibular Molars 1a, 2a, and 2b. Sections from 3 different teeth are shown. Note the dentin layer lack dentinal tubules, is much thinner than the enamel layer, and shows vacancies presumably from cellular debris. Note the well-defined rod and interrod architecture in the enamel.

Dspp$^{+/-1fs}$ D14 Molars

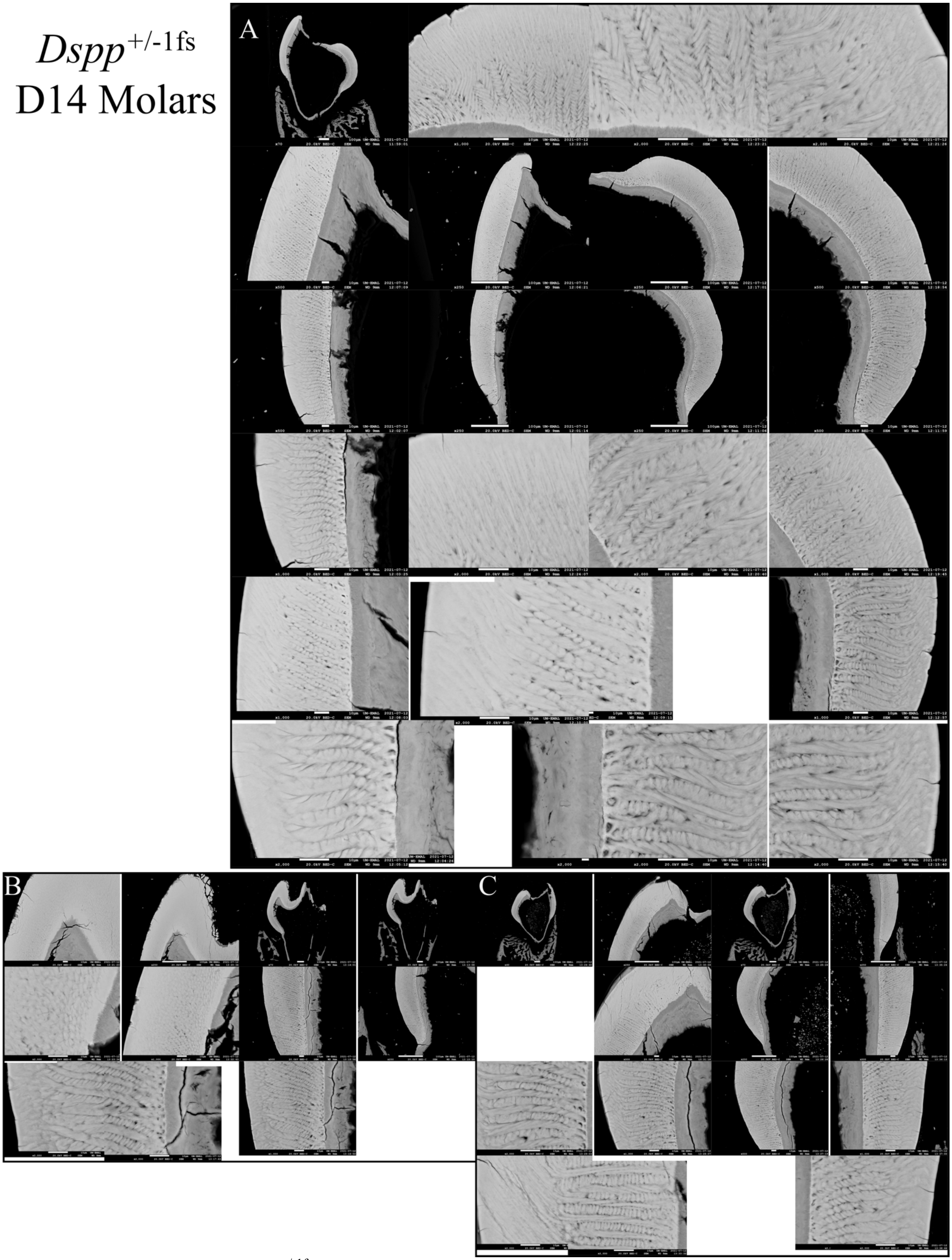

Fig. S14B. bSEM of D14 *Dspp*$^{+/-1fs}$ Mandibular Molars 1b, 3a, and 3b. Sections from 3 different teeth are shown. Note the dentin layer lack dentinal tubules, is much thinner than the enamel layer, and shows vacancies presumably from cellular debris. Note the well-defined rod and interrod architecture in the enamel.

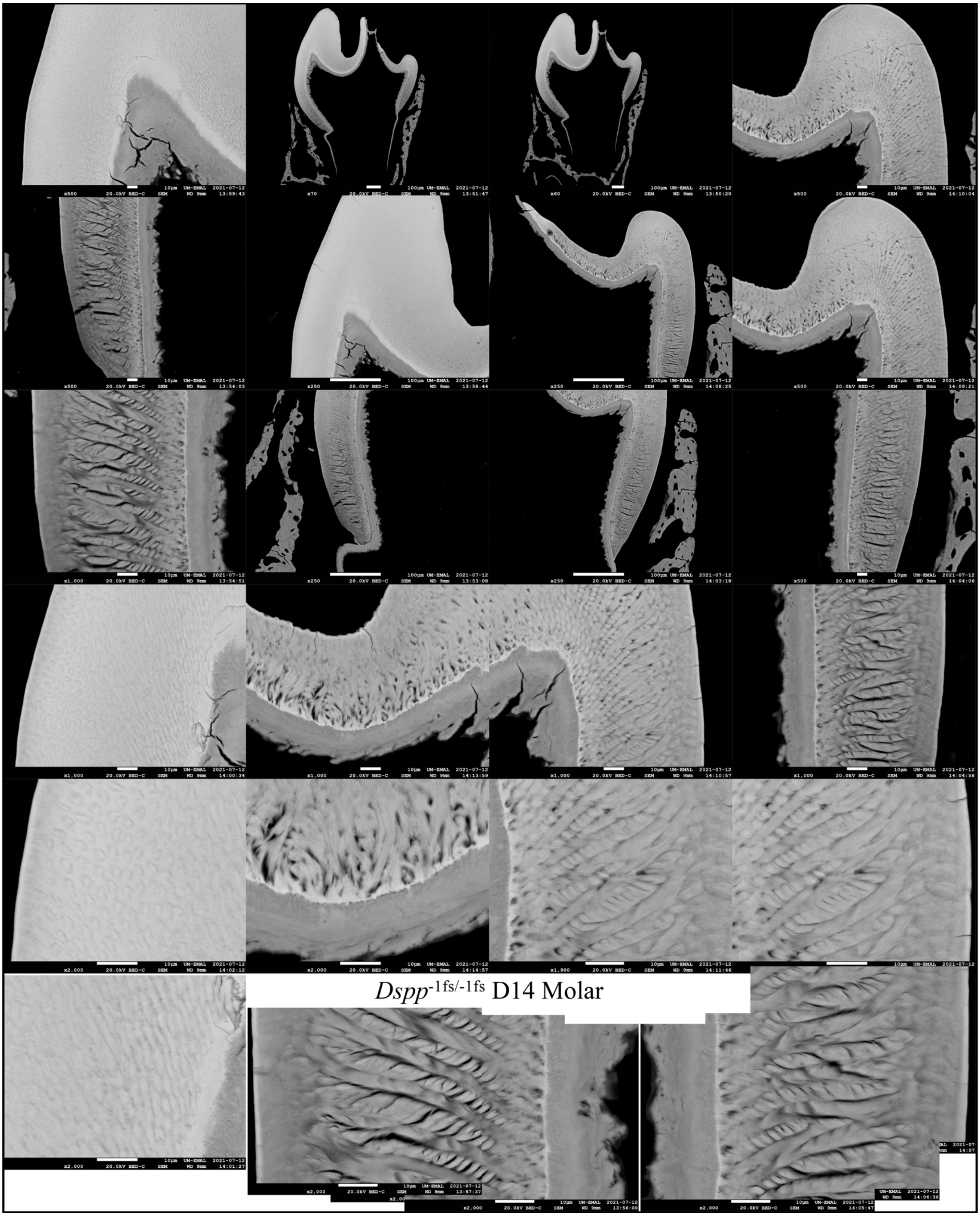

Fig. S15A. bSEM of D14 *Dspp*$^{-1fs/-1fs}$ Mandibular Molar 1a. Sections from a single tooth are shown. Note the dentin layer lack dentinal tubules, is much thinner than the enamel layer, and shows vacancies presumably from cellular debris. Lines in dentin run parallel to the DEJ. Note the well-defined rod and interrod architecture in the enamel. In this tooth the more cervical enamel is grayer, suggesting hypomineralization.

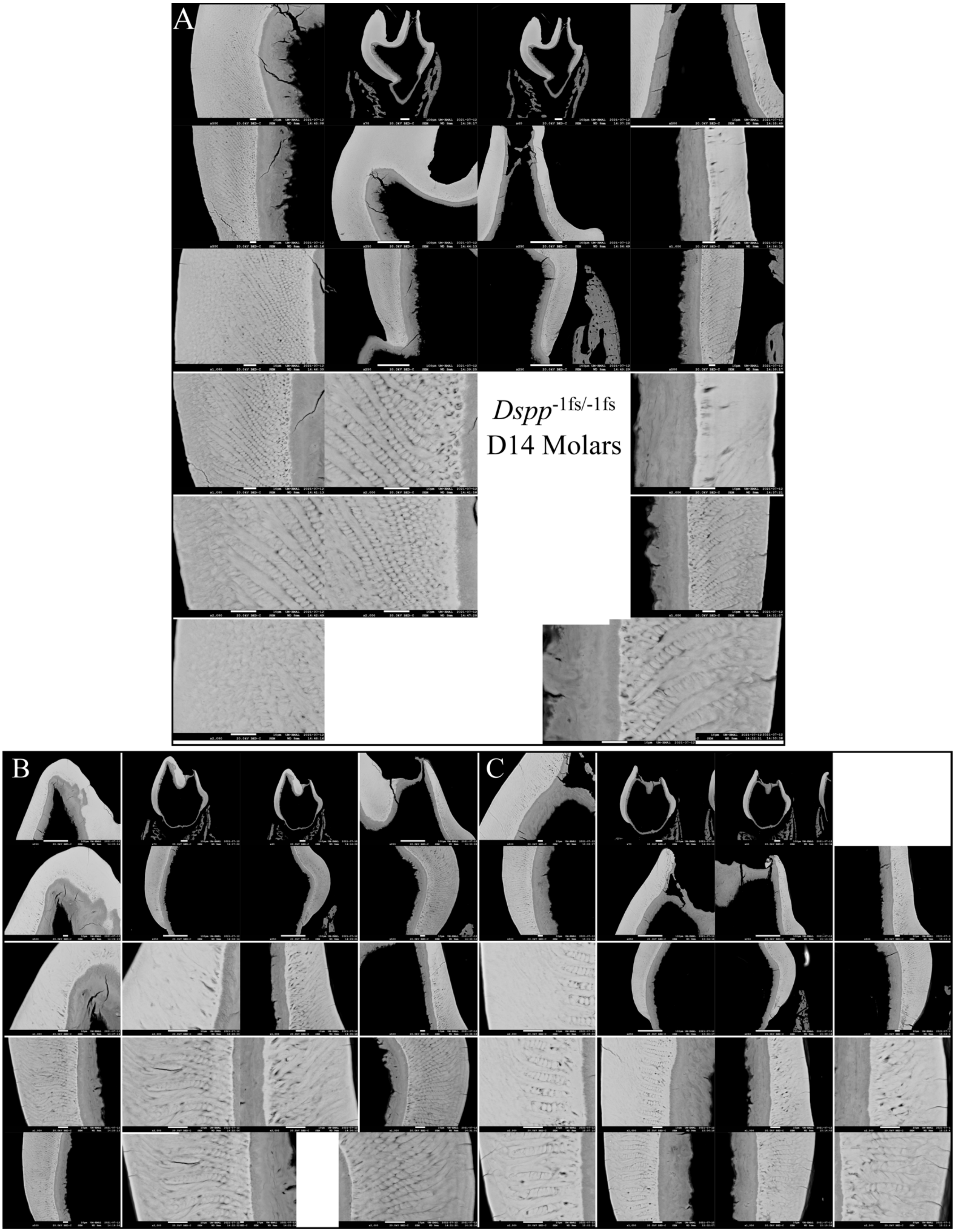

Fig. S15B. bSEM of D14 *Dspp*$^{-1fs/-1fs}$ Mandibular Molars 1b, 2a, and 2b bSEM. Sections from three teeth are shown. Note the lack of dentinal tubules in the dentin layer, which is much thinner than the enamel layer, and shows vacancies presumably from cellular debris. Lines in dentin run parallel to the DEJ. Note the well-defined rod and interrod architecture in the enamel.

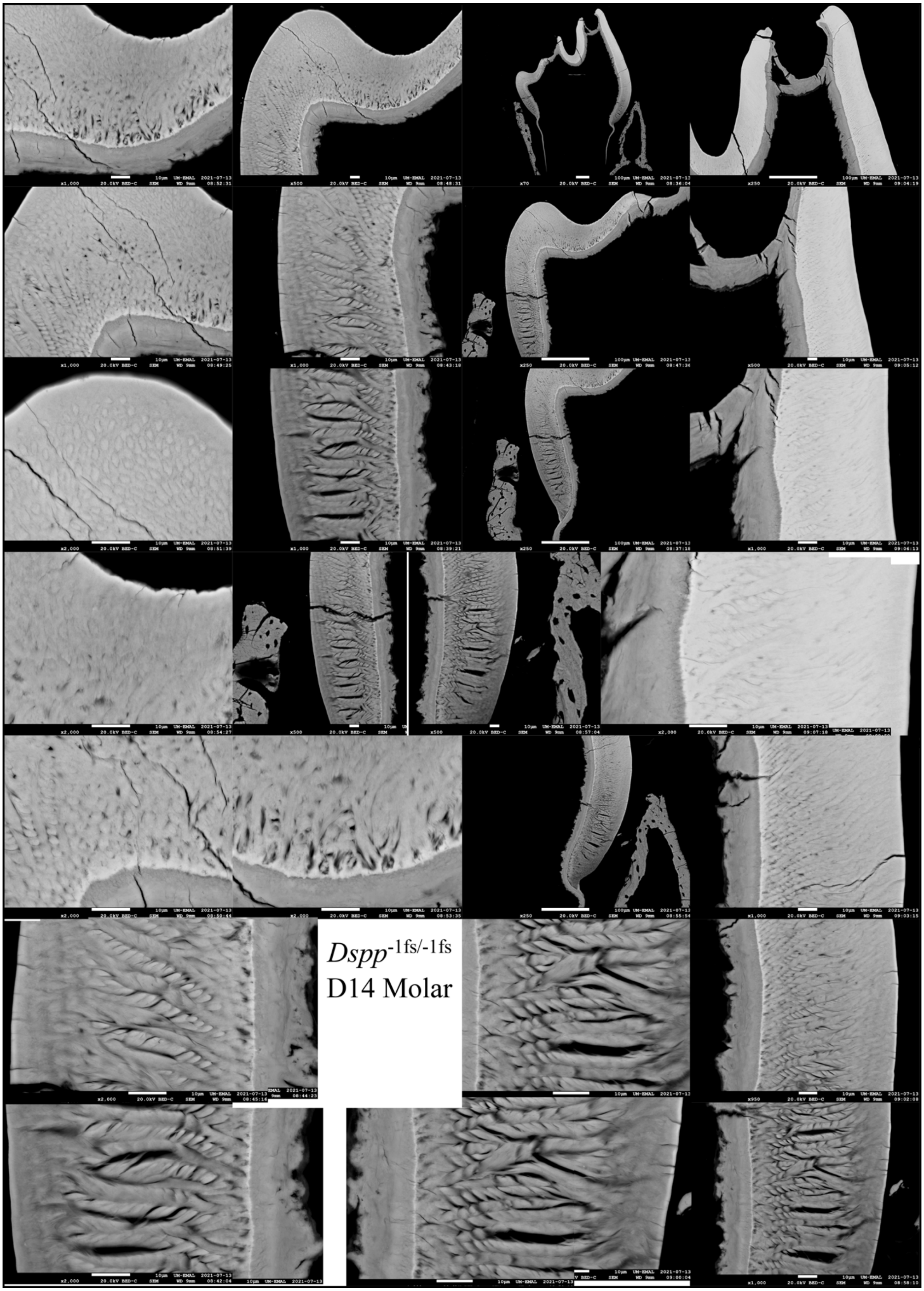

Fig. S15C. bSEM of $Dspp^{-1fs/-1fs}$ D14 Molar 3a. Sections from a single tooth are shown. Note the dentin layer lacks dentinal tubules, is much thinner than the enamel layer, and shows vacancies presumably from cellular debris. Lines in dentin run parallel to the DEJ. Note the well-defined rod and interrod architecture in the enamel. In this tooth the more cervical enamel is grayer, suggesting hypomineralization.

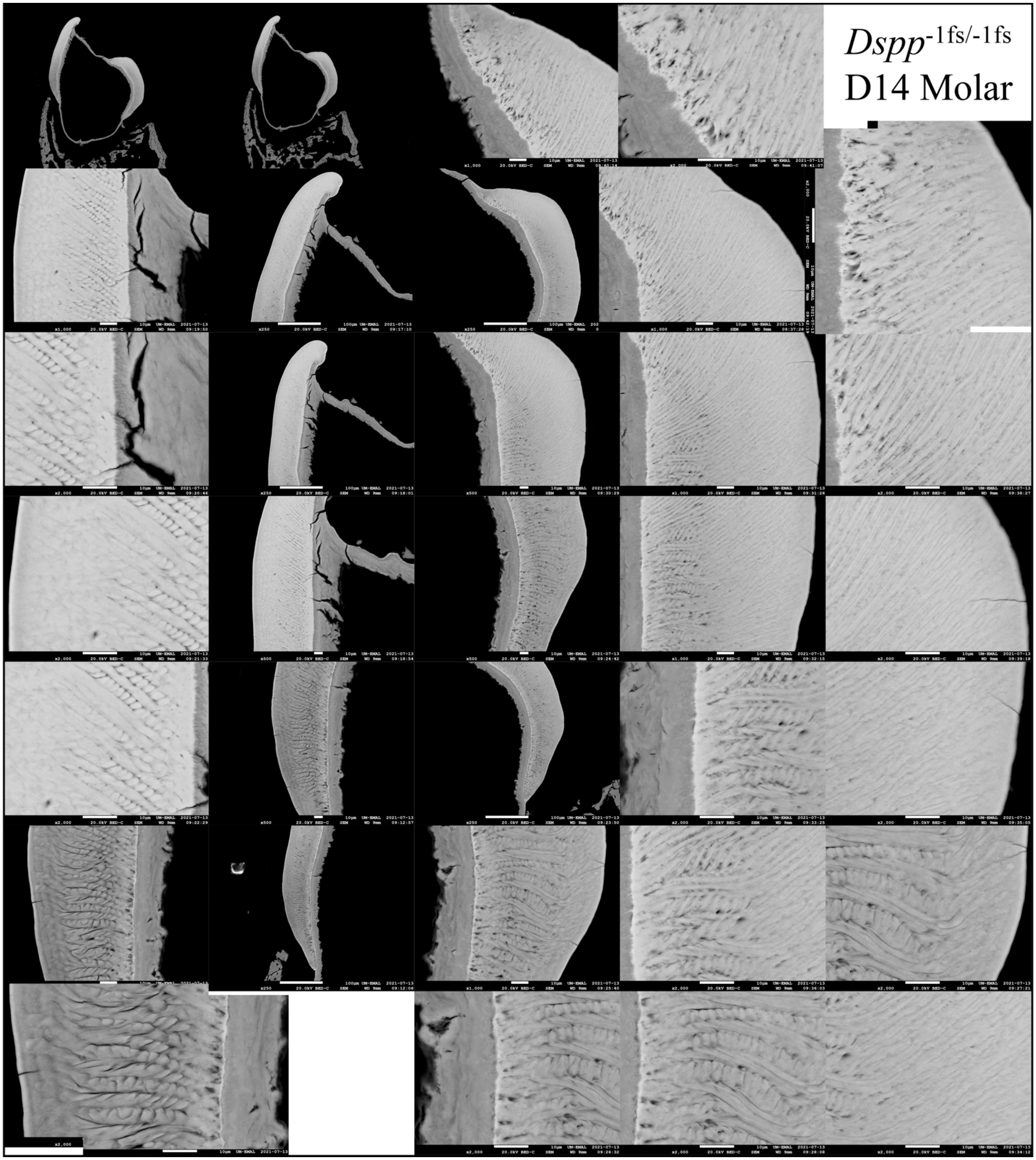

Fig. S15D. bSEM of *Dspp*$^{-1fs/-1fs}$ D14 Molar 3b. Sections from a single tooth are shown. Note the dentin layer lacks dentinal tubules, is much thinner than the enamel layer, and shows vacancies presumably from cellular debris. Lines in dentin run parallel to the DEJ. Note the well-defined rod and interrod architecture in the enamel. In this tooth the more cervical enamel is grayer, suggesting hypomineralization.

A *Dspp*$^{+/-1fs}$ 7-week Mandibular Molars

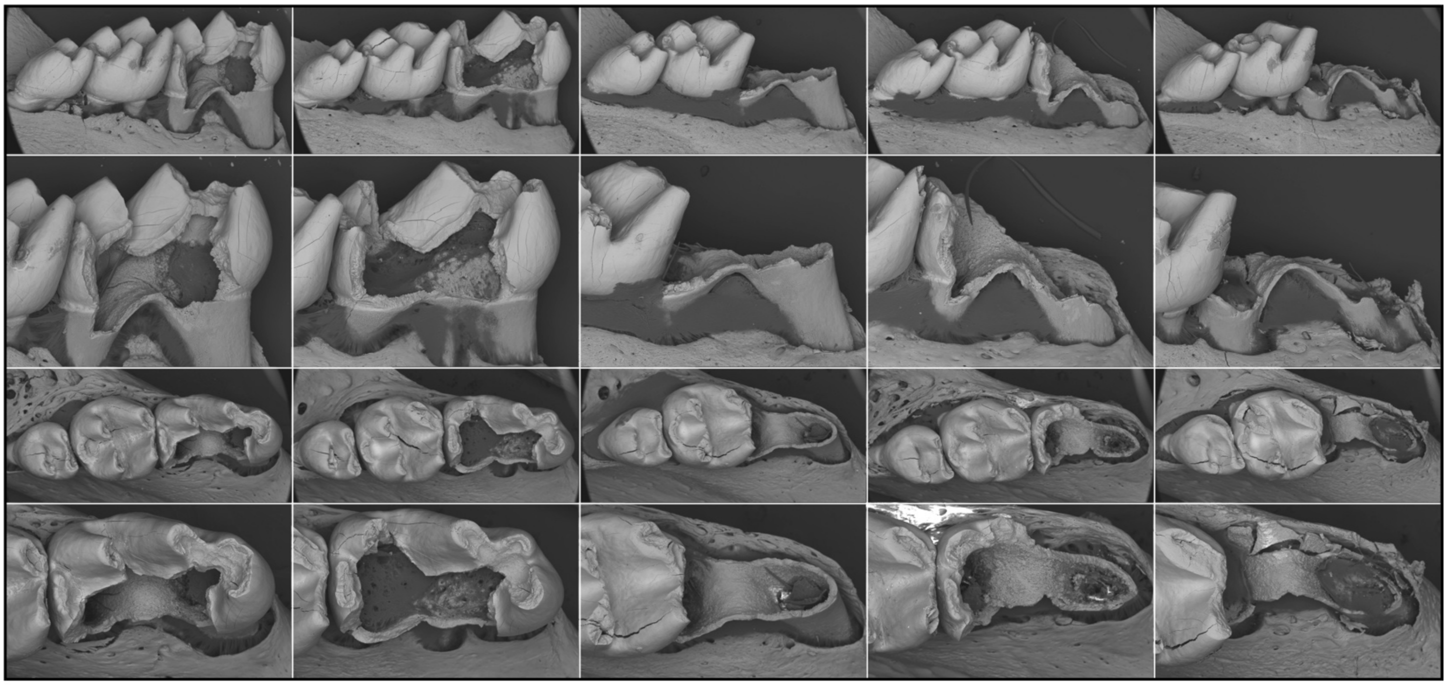

B *Dspp*$^{-1fs/-1fs}$ 7-week Mandibular Molars

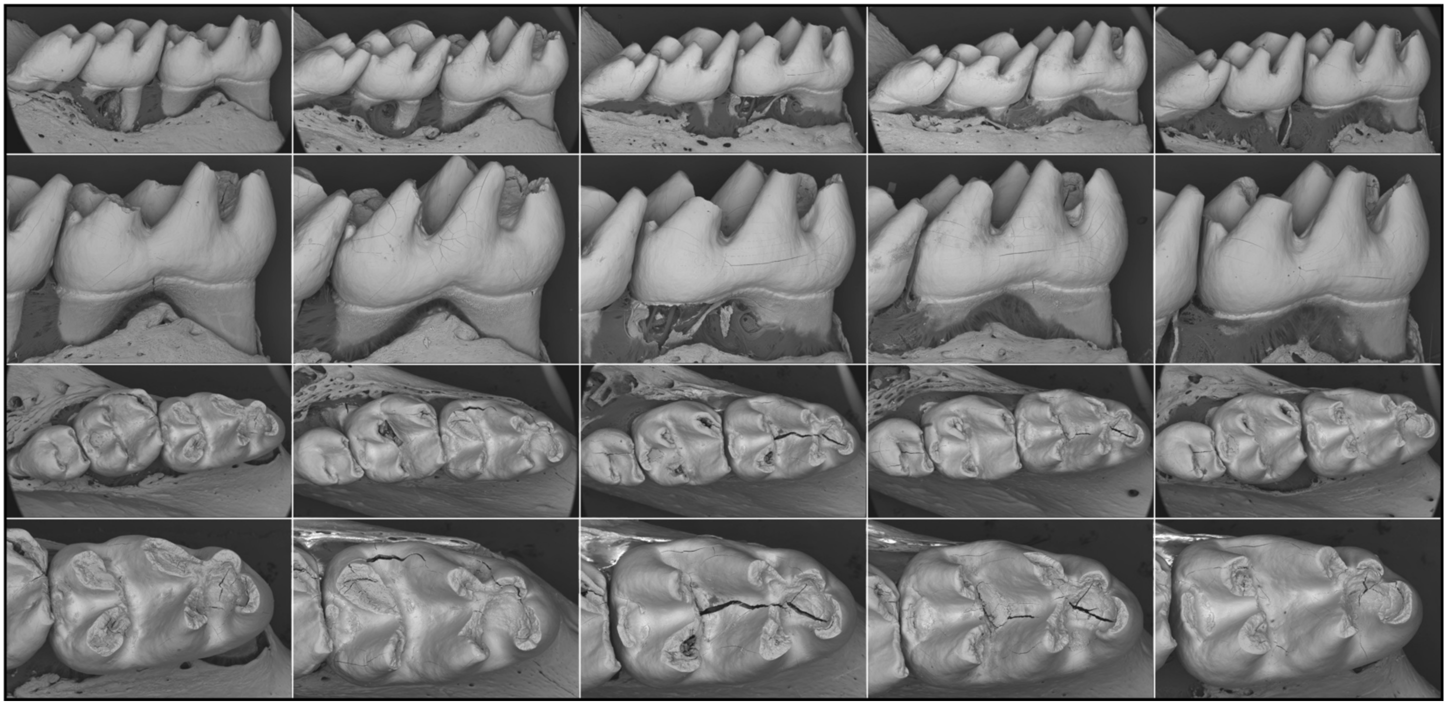

Figure S16. bSEM of a 7-Week *Dspp*$^{+/-1fs}$ and *Dspp*$^{-1fs/-1fs}$ Mouse Mandibular Molars. Note the crown and root failure of *Dspp*$^{-1fs/-1fs}$ molars at 7-weeks. Mouse molars only have about 2 weeks to form before they erupt. Because of this, when the molars erupt at D14 the very slow rate of reparative dentin formation forces them to function with a very thin dentin thickness, causing the crowns to fail.

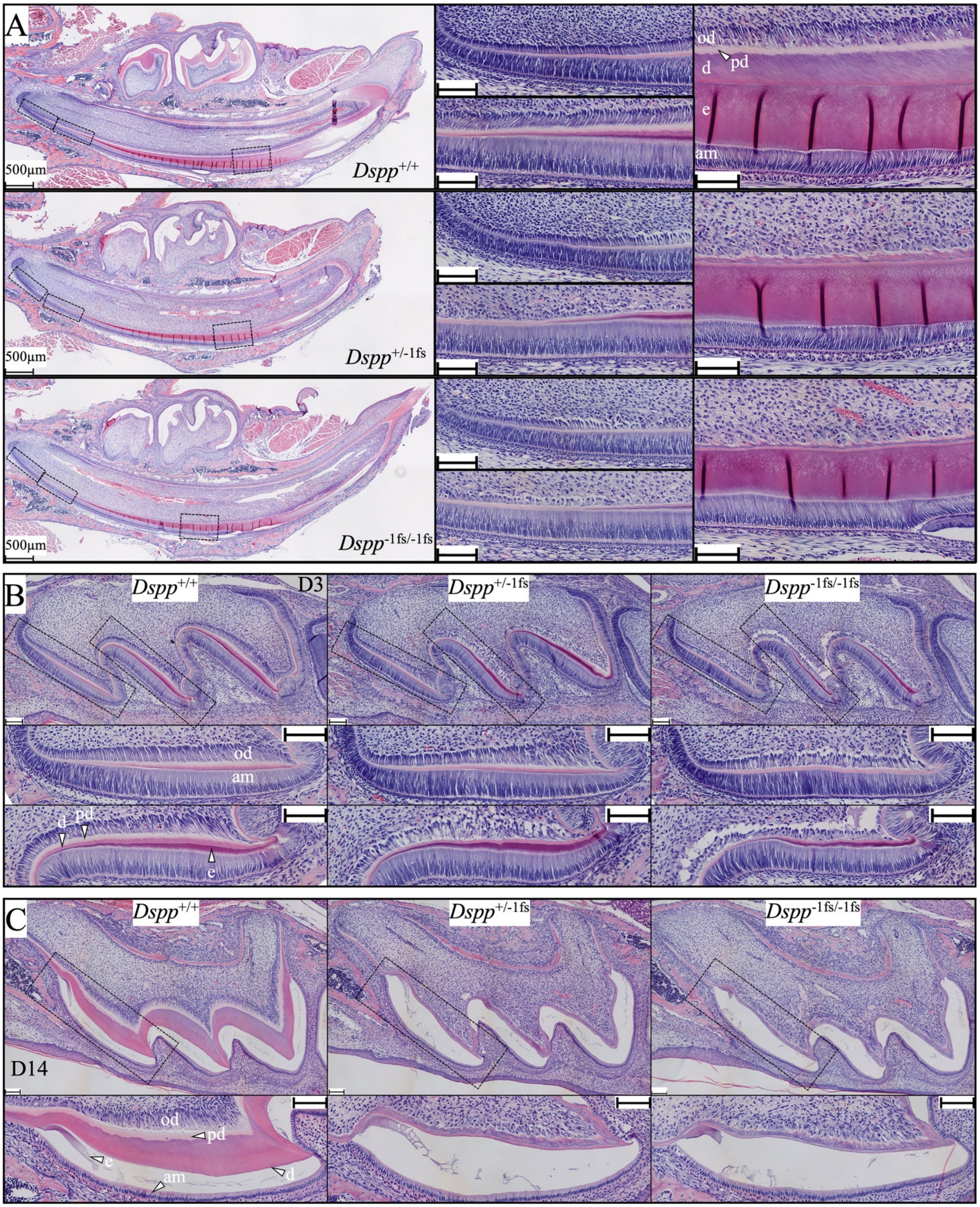

Figure S17. Histology of D14 Mandibular Incisors, D3 and D14 Maxillary Molars of Wild-Type, *Dspp*$^{+/-1fs}$ and *Dspp*$^{-1fs/-1fs}$ Mice. ***A:*** *left:* D14 mandibular incisor; *center:* higher magnification views of early dentin and enamel formation corresponding to the locations the two boxes in the low magnification panel; *right:* higher magnification views of later dentin and enamel formation after the enamel layer has reached full-thickness. ***B:*** H&E staining of D3 maxillary 1st molars. Boxes mark the positions of the magnified views below. The *Dspp*$^{+/-1fs}$ and *Dspp*$^{-1fs/-1fs}$ mice showed abnormally thin dentin. Pathosis in the odontoblast layer is evident in the *Dspp*$^{+/-1fs}$ and *Dspp*$^{-1fs/-1fs}$ mice. The morphology of ameloblasts is normal. ***C:*** D14 maxillary first molars severe decreases in predentin and dentin deposition. The morphology of ameloblasts is normal. ***Key:*** am, ameloblasts; d, dentin; e, enamel; o, odontoblasts; p, predentin. Scale bars: 100 µm.

Enamel		H(GPa)			H(GPa)			H(GPa)			H(GPa)			H(GPa)			H(GPa)
WT-1	F1	3.645	+/fs1	F1	3.556	fs/fs1	F1	3.914	WT-1	F1	3.645	+/N	F1	1.620	N/N	F1	3.086
WT-2	F1	3.396	+/fs2	F1	3.597	fs/fs2	F1	2.298	WT-2	F1	3.396	+/N	F1	3.755	N/N	F1	2.730
WT-3	F1	2.808	+/fs3	F1	3.510	fs/fs3	F1	3.412	WT-3	F1	2.808	+/N	F1	3.752	N/N	F1	2.407
WT-4	F1	3.604	+/fs4	F1	3.791	fs/fs4	F1	3.570	WT-4	F1	3.604						
WT-5	F1	3.532	+/fs5	F1	3.072	fs/fs5	F1	3.854	WT-5	F1	3.532						
WT-6	F1	3.285	+/fs6	F1	4.055	fs/fs6	F1	3.735	WT-6	F1	3.285						
WT-1	G1	3.412	+/fs1	G1	3.740	fs/fs1	G1	4.010	WT-1	G1	3.412	+/N	G1	1.562	N/N	G1	3.381
WT-2	G1	3.411	+/fs2	G1	3.224	fs/fs2	G1	3.370	WT-2	G1	3.411	+/N	G1	3.396	N/N	G1	2.532
WT-3	G1	3.567	+/fs3	G1	3.740	fs/fs3	G1	2.452	WT-3	G1	3.567	+/N	G1	3.375	N/N	G1	2.194
WT-4	G1	3.609	+/fs4	G1	3.466	fs/fs4	G1	3.411	WT-4	G1	3.609						
WT-5	G1	3.420	+/fs5	G1	2.087	fs/fs5	G1	3.903	WT-5	G1	3.420						
WT-6	G1	3.874	+/fs6	G1	3.093	fs/fs6	G1	3.949	WT-6	G1	3.874						
WT-1	F2	3.668	+/fs1	F2	3.470	fs/fs1	F2	3.757	WT-1	F2	3.668	+/N	F2	0.863	N/N	F2	2.470
WT-2	F2	3.675	+/fs2	F2	3.770	fs/fs2	F2	3.430	WT-2	F2	3.675	+/N	F2	3.339	N/N	F2	2.482
WT-3	F2	3.780	+/fs3	F2	4.155	fs/fs3	F2	3.614	WT-3	F2	3.780	+/N	F2	3.503	N/N	F2	2.195
WT-4	F2	3.781	+/fs4	F2	3.124	fs/fs4	F2	2.993	WT-4	F2	3.781						
WT-5	F2	3.869	+/fs5	F2	3.901	fs/fs5	F2	3.567	WT-5	F2	3.869						
WT-6	F2	3.602	+/fs6	F2	3.240	fs/fs6	F2	3.539	WT-6	F2	3.602						
WT-1	G2	3.546	+/fs1	G2	3.740	fs/fs1	G2	3.668	WT-1	G2	3.546	+/N	G2	1.009	N/N	G2	3.341
WT-2	G2	3.445	+/fs2	G2	3.597	fs/fs2	G2	3.659	WT-2	G2	3.445	+/N	G2	2.827	N/N	G2	2.456
WT-3	G2	3.296	+/fs3	G2	3.204	fs/fs3	G2	3.655	WT-3	G2	3.296	+/N	G2	3.378	N/N	G2	1.938
WT-4	G2	4.004	+/fs4	G2	3.402	fs/fs4	G2	3.576	WT-4	G2	4.004						
WT-5	G2	3.731	+/fs5	G2	2.908	fs/fs5	G2	3.930	WT-5	G2	3.731						
WT-6	G2	3.488	+/fs6	G2	3.362	fs/fs6	G2	3.665	WT-6	G2	3.488						
WT-1	F3	4.033	+/fs1	F3	4.409	fs/fs1	F3	4.393	WT-1	F3	4.033	+/N	F3	1.192	N/N	F3	4.817
WT-2	F3	4.644	+/fs2	F3	4.471	fs/fs2	F3	4.499	WT-2	F3	4.644	+/N	F3	4.046	N/N	F3	2.865
WT-3	F3	3.734	+/fs3	F3	4.786	fs/fs3	F3	3.985	WT-3	F3	3.734	+/N	F3	4.469	N/N	F3	2.339
WT-4	F3	4.675	+/fs4	F3	4.280	fs/fs4	F3	4.313	WT-4	F3	4.675						
WT-5	F3	4.541	+/fs5	F3	4.669	fs/fs5	F3	4.006	WT-5	F3	4.541						
WT-6	F3	4.689	+/fs6	F3	4.573	fs/fs6	F3	4.518	WT-6	F3	4.689						
WT-1	G3	4.587	+/fs1	G3	4.344	fs/fs1	G3	4.105	WT-1	G3	4.587	+/N	G3	1.384	N/N	G3	4.227
WT-2	G3	4.123	+/fs2	G3	4.012	fs/fs2	G3	4.312	WT-2	G3	4.123	+/N	G3	4.030	N/N	G3	4.099
WT-3	G3	3.994	+/fs3	G3	4.035	fs/fs3	G3	4.126	WT-3	G3	3.994	+/N	G3	4.268	N/N	G3	3.439
WT-4	G3	4.196	+/fs4	G3	4.194	fs/fs4	G3	4.174	WT-4	G3	4.196						
WT-5	G3	4.727	+/fs5	G3	4.597	fs/fs5	G3	4.917	WT-5	G3	4.727						
WT-6	G3	4.028	+/fs6	G3	4.019	fs/fs6	G3	3.854	WT-6	G3	4.028						

Table S1. Raw Data for Enamel Hardness Testing. The -1fs designation is here abbreviated as "fs" and the N-terminal P19L designation is abbreviated as "N". The background colors correspond to those in the plots of Figure 3.

Dentin		H(GPa)			H(GPa)			H(GPa)			H(GPa)			H(GPa)			H(GPa)
WT-1	B1	1.496	+/fs1	B1	0.887	fs/fs1	B1	0.904	WT-1	B1	1.496	+/N	B1	1.471	N/N	B1	1.244
WT-2	B1	1.332	+/fs2	B1	1.003	fs/fs2	B1	0.829	WT-2	B1	1.332	+/N	B1	1.291	N/N	B1	1.151
WT-3	B1	1.067	+/fs3	B1	0.893	fs/fs3	B1	0.852	WT-3	B1	1.067	+/N	B1	1.079	N/N	B1	1.253
WT-4	B1	1.388	+/fs4	B1	1.061	fs/fs4	B1	0.831	WT-4	B1	1.388						
WT-5	B1	1.438	+/fs5	B1	0.918	fs/fs5	B1	0.953	WT-5	B1	1.438						
WT-6	B1	1.547	+/fs6	B1	0.891	fs/fs6	B1	0.786	WT-6	B1	1.547						
WT-1	C1	1.453	+/fs1	C1	0.731	fs/fs1	C1	0.854	WT-1	C1	1.453	+/N	C1	1.292	N/N	C1	1.424
WT-2	C1	1.177	+/fs2	C1	0.699	fs/fs2	C1	0.938	WT-2	C1	1.177	+/N	C1	1.295	N/N	C1	1.215
WT-3	C1	1.255	+/fs3	C1	0.851	fs/fs3	C1	0.950	WT-3	C1	1.255	+/N	C1	1.350	N/N	C1	1.165
WT-4	C1	1.363	+/fs4	C1	0.782	fs/fs4	C1	0.928	WT-4	C1	1.363						
WT-5	C1	1.346	+/fs5	C1	1.065	fs/fs5	C1	0.962	WT-5	C1	1.346						
WT-6	C1	1.382	+/fs6	C1	0.693	fs/fs6	C1	0.846	WT-6	C1	1.382						
WT-1	D1	1.410	+/fs1	D1	0.823	fs/fs1	D1	0.854	WT-1	D1	1.410	+/N	D1	1.222	N/N	D1	1.052
WT-2	D1	1.329	+/fs2	D1	1.024	fs/fs2	D1	0.805	WT-2	D1	1.329	+/N	D1	1.203	N/N	D1	1.169
WT-3	D1	1.417	+/fs3	D1	1.006	fs/fs3	D1	0.887	WT-3	D1	1.417	+/N	D1	1.205	N/N	D1	1.229
WT-4	D1	1.336	+/fs4	D1	0.710	fs/fs4	D1	0.764	WT-4	D1	1.336						
WT-5	D1	1.429	+/fs5	D1	1.150	fs/fs5	D1	0.940	WT-5	D1	1.429						
WT-6	D1	1.507	+/fs6	D1	1.167	fs/fs6	D1	0.912	WT-6	D1	1.507						
WT-1	E1	1.382	+/fs1	E1	1.177	fs/fs1	E1	0.708	WT-1	E1	1.382	+/N	E1	1.140	N/N	E1	1.158
WT-2	E1	1.288	+/fs2	E1	1.167	fs/fs2	E1	0.948	WT-2	E1	1.287	+/N	E1	1.266	N/N	E1	1.116
WT-3	E1	1.331	+/fs3	E1	1.052	fs/fs3	E1	1.118	WT-3	E1	1.331	+/N	E1	1.071	N/N	E1	1.431
WT-4	E1	1.327	+/fs4	E1	1.019	fs/fs4	E1	0.985	WT-4	E1	1.327						
WT-5	E1	1.404	+/fs5	E1	1.011	fs/fs5	E1	0.929	WT-5	E1	1.404						
WT-6	E1	1.475	+/fs6	E1	0.896	fs/fs6	E1	0.918	WT-6	E1	1.475						
WT-1	B2	1.421	+/fs1	B2	0.731	fs/fs1	B2	0.749	WT-1	B2	1.421	+/N	B2	1.079	N/N	B2	0.785
WT-2	B2	1.296	+/fs2	B2	0.699	fs/fs2	B2	0.790	WT-2	B2	1.296	+/N	B2	1.089	N/N	B2	0.973
WT-3	B2	1.244	+/fs3	B2	0.851	fs/fs3	B2	0.870	WT-3	B2	1.244	+/N	B2	1.115	N/N	B2	0.982
WT-4	B2	1.296	+/fs4	B2	0.782	fs/fs4	B2	0.736	WT-4	B2	1.296						
WT-5	B2	1.279	+/fs5	B2	1.065	fs/fs5	B2	0.678	WT-5	B2	1.279						
WT-6	B2	1.323	+/fs6	B2	0.693	fs/fs6	B2	0.711	WT-6	B2	1.323						
WT-1	C2	1.315	+/fs1	C2	0.901	fs/fs1	C2	0.749	WT-1	C2	1.315	+/N	C2	0.944	N/N	C2	0.905
WT-2	C2	1.174	+/fs2	C2	1.109	fs/fs2	C2	0.790	WT-2	C2	1.174	+/N	C2	1.032	N/N	C2	0.881
WT-3	C2	0.987	+/fs3	C2	0.837	fs/fs3	C2	0.870	WT-3	C2	0.987	+/N	C2	1.147	N/N	C2	1.172
WT-4	C2	1.337	+/fs4	C2	0.761	fs/fs4	C2	0.736	WT-4	C2	1.337						
WT-5	C2	1.245	+/fs5	C2	0.477	fs/fs5	C2	0.678	WT-5	C2	1.245						
WT-6	C2	1.349	+/fs6	C2	0.863	fs/fs6	C2	0.711	WT-6	C2	1.347						
WT-1	D2	1.449	+/fs1	D2	0.891	fs/fs1	D2	0.802	WT-1	D2	1.449	+/N	D2	1.069	N/N	D2	1.160
WT-2	D2	1.375	+/fs2	D2	0.730	fs/fs2	D2	0.686	WT-2	D2	1.375	+/N	D2	1.307	N/N	D2	1.071
WT-3	D2	1.375	+/fs3	D2	0.946	fs/fs3	D2	1.028	WT-3	D2	1.375	+/N	D2	1.148	N/N	D2	1.249
WT-4	D2	1.410	+/fs4	D2	0.672	fs/fs4	D2	0.716	WT-4	D2	1.410						
WT-5	D2	1.370	+/fs5	D2	0.756	fs/fs5	D2	0.748	WT-5	D2	1.370						
WT-6	D2	1.173	+/fs6	D2	0.873	fs/fs6	D2	0.709	WT-6	D2	1.173						
WT-1	E2	1.502	+/fs1	E2	0.848	fs/fs1	E2	0.768	WT-1	E2	1.502	+/N	E2	1.137	N/N	E2	1.175
WT-2	E2	1.517	+/fs2	E2	0.615	fs/fs2	E2	0.826	WT-2	E2	1.517	+/N	E2	1.284	N/N	E2	1.153
WT-3	E2	1.527	+/fs3	E2	0.893	fs/fs3	E2	0.752	WT-3	E2	1.527	+/N	E2	1.280	N/N	E2	1.021
WT-4	E2	1.512	+/fs4	E2	0.601	fs/fs4	E2	0.746	WT-4	E2	1.512						
WT-5	E2	1.572	+/fs5	E2	0.867	fs/fs5	E2	0.856	WT-5	E2	1.572						
WT-6	E2	1.453	+/fs6	E2	0.696	fs/fs6	E2	0.666	WT-6	E2	1.453						

Table S2. Raw Data for Dentin Hardness Testing (part 1). The -1fs designation is here abbreviated as "fs" and the N-terminal P19L designation is abbreviated as "N". The background colors correspond to those in the plots of Figure 3.

Dentin		H(GPa)			H(GPa)			H(GPa)			H(GPa)			H(GPa)			H(GPa)
WT-1	B3	1.076	+/fs1	B3	0.823	fs/fs1	B3	0.935	WT-1	B3	1.076	+/N	B3	0.787	N/N	B3	0.727
WT-2	B3	0.922	+/fs2	B3	1.024	fs/fs2	B3	0.874	WT-2	B3	0.922	+/N	B3	0.896	N/N	B3	0.772
WT-3	B3	1.031	+/fs3	B3	1.006	fs/fs3	B3	0.912	WT-3	B3	1.031	+/N	B3	0.999	N/N	B3	0.758
WT-4	B3	0.852	+/fs4	B3	0.710	fs/fs4	B3	0.887	WT-4	B3	0.852						
WT-5	B3	1.089	+/fs5	B3	1.150	fs/fs5	B3	0.876	WT-5	B3	1.089						
WT-6	B3	1.040	+/fs6	B3	1.167	fs/fs6	B3	0.752	WT-6	B3	1.040						
WT-1	C3	0.885	+/fs1	C3	0.901	fs/fs1	C3	0.829	WT-1	C3	0.885	+/N	C3	0.812	N/N	C3	1.051
WT-2	C3	0.974	+/fs2	C3	1.109	fs/fs2	C3	0.710	WT-2	C3	0.974	+/N	C3	0.889	N/N	C3	0.091
WT-3	C3	0.896	+/fs3	C3	0.837	fs/fs3	C3	0.821	WT-3	C3	0.896	+/N	C3	0.873	N/N	C3	0.905
WT-4	C3	0.925	+/fs4	C3	0.761	fs/fs4	C3	0.801	WT-4	C3	0.925						
WT-5	C3	0.892	+/fs5	C3	0.477	fs/fs5	C3	0.756	WT-5	C3	0.892						
WT-6	C3	0.939	+/fs6	C3	0.863	fs/fs6	C3	0.846	WT-6	C3	0.939						
WT-1	D3	1.247	+/fs1	D3	0.685	fs/fs1	D3	0.836	WT-1	D3	1.247	+/N	D3	1.034	N/N	D3	1.062
WT-2	D3	1.028	+/fs2	D3	0.935	fs/fs2	D3	0.826	WT-2	D3	1.028	+/N	D3	0.690	N/N	D3	0.784
WT-3	D3	0.862	+/fs3	D3	0.566	fs/fs3	D3	0.668	WT-3	D3	0.862	+/N	D3	0.876	N/N	D3	1.234
WT-4	D3	0.960	+/fs4	D3	0.642	fs/fs4	D3	0.689	WT-4	D3	0.960						
WT-5	D3	1.045	+/fs5	D3	0.647	fs/fs5	D3	0.802	WT-5	D3	1.045						
WT-6	D3	1.100	+/fs6	D3	0.674	fs/fs6	D3	0.823	WT-6	D3	1.100						
WT-1	E3	1.115	+/fs1	E3	0.641	fs/fs1	E3	0.721	WT-1	E3	1.115	+/N	E3	0.895	N/N	E3	0.947
WT-2	E3	0.940	+/fs2	E3	0.780	fs/fs2	E3	0.817	WT-2	E3	0.940	+/N	E3	0.801	N/N	E3	0.980
WT-3	E3	0.989	+/fs3	E3	0.865	fs/fs3	E3	0.903	WT-3	E3	0.989	+/N	E3	0.847	N/N	E3	0.947
WT-4	E3	1.041	+/fs4	E3	0.758	fs/fs4	E3	0.797	WT-4	E3	1.041						
WT-5	E3	0.814	+/fs5	E3	0.752	fs/fs5	E3	0.777	WT-5	E3	0.814						
WT-6	E3	1.044	+/fs6	E3	0.623	fs/fs6	E3	0.810	WT-6	E3	1.044						

Table S2 (continued). Raw Data for Dentin Hardness Testing. The -1fs designation is here abbreviated as "fs" and the N-terminal P19L designation is abbreviated as "N". The background colors correspond to those in the plots of Figure 3.

Bone		H(GPa)			H(GPa)			H(GPa)			H(GPa)			H(GPa)			H(GPa)
WT1	A1	1.222	+/fs1	A1	1.155	fs/fs1	A1	1.278	WT1	A1	1.222	+/N	A1	1.509	N/N	A1	1.045
WT2	A1	1.171	+/fs2	A1	1.021	fs/fs2	A1	1.173	WT2	A1	1.171	+/N	A1	1.289	N/N	A1	1.454
WT3	A1	1.201	+/fs3	A1	1.296	fs/fs3	A1	1.550	WT3	A1	1.201	+/N	A1	1.421	N/N	A1	1.434
WT4	A1	1.463	+/fs4	A1	1.445	fs/fs4	A1	1.381	WT4	A1	1.463						
WT5	A1	1.504	+/fs5	A1	1.250	fs/fs5	A1	1.554	WT5	A1	1.504						
WT6	A1	1.336	+/fs6	A1	1.514	fs/fs6	A1	1.223	WT6	A1	1.336						
WT1	A2	1.317	+/fs1	A2	0.893	fs/fs1	A2	1.292	WT1	A2	1.317	+/N	A2	1.151	N/N	A2	1.046
WT2	A2	0.996	+/fs2	A2	1.180	fs/fs2	A2	1.255	WT2	A2	0.996	+/N	A2	1.261	N/N	A2	1.078
WT3	A2	1.044	+/fs3	A2	0.774	fs/fs3	A2	0.999	WT3	A2	1.044	+/N	A2	1.249	N/N	A2	1.192
WT4	A2	1.160	+/fs4	A2	1.062	fs/fs4	A2	1.113	WT4	A2	1.160						
WT5	A2	1.127	+/fs5	A2	1.199	fs/fs5	A2	1.072	WT5	A2	1.127						
WT6	A2	1.058	+/fs6	A2	0.937	fs/fs6	A2	1.166	WT6	A2	1.058						
WT1	A3	1.002	+/fs1	A3	0.962	fs/fs1	A3	1.116	WT1	A3	1.002	+/N	A3	0.793	N/N	A3	0.812
WT2	A3	0.993	+/fs2	A3	0.918	fs/fs2	A3	1.126	WT2	A3	0.993	+/N	A3	0.871	N/N	A3	1.032
WT3	A3	1.057	+/fs3	A3	1.005	fs/fs3	A3	1.079	WT3	A3	1.057	+/N	A3	1.041	N/N	A3	0.699
WT4	A3	1.041	+/fs4	A3	1.014	fs/fs4	A3	1.228	WT4	A3	1.041						
WT5	A3	0.819	+/fs5	A3	0.783	fs/fs5	A3	0.987	WT5	A3	0.819						
WT6	A3	1.071	+/fs6	A3	0.739	fs/fs6	A3	1.066	WT6	A3	1.071						

Table S3. Raw Data for Bone Hardness Testing. The -1fs designation is here abbreviated as "fs" and the N-terminal P19L designation is abbreviated as "N". The background colors correspond to those in the plots of Figure 3.

References in Supplemental Data

1. Cong, L. *et al.* Multiplex genome engineering using CRISPR/Cas systems. *Science.* **339,** 819-823 (2013).

2. Jinek, M. *et al.* RNA-programmed genome editing in human cells. *Elife.* **2:e00471.,** 10.7554/eLife.00471. (2013).

3. Mali, P., Esvelt, K. M. & Church, G. M. Cas9 as a versatile tool for engineering biology. *Nat Methods.* **10,** 957-963 (2013).

4. Hsu, P. D. *et al.* DNA targeting specificity of RNA-guided Cas9 nucleases. *Nat Biotechnol* **31,** 827-832 (2013).

5. Ran, F. A. *et al.* Genome engineering using the CRISPR-Cas9 system. *Nat Protoc* **8,** 2281-2308 (2013).

6. McBurney, M. W., Fournier, S., Jardine, K. & Sutherland, L. Intragenic regions of the murine Pgk-1 locus enhance integration of transfected DNAs into genomes of embryonal carcinoma cells. *Somat Cell Mol Genet* **20,** 515-528 (1994).

7. Pettitt, S. J. *et al.* Agouti C57BL/6N embryonic stem cells for mouse genetic resources. *Nat Methods* **6,** 493-495 (2009).

8. Sakurai, T., Watanabe, S., Kamiyoshi, A., Sato, M. & Shindo, T. A single blastocyst assay optimized for detecting CRISPR/Cas9 system-induced indel mutations in mice. *BMC Biotechnol.* **14,** 69 (2014).
